# Two Measures of Dependence

**DOI:** 10.3390/e21080778

**Published:** 2019-08-08

**Authors:** Amos Lapidoth, Christoph Pfister

**Affiliations:** Signal and Information Processing Laboratory, ETH Zurich, 8092 Zurich, Switzerland

**Keywords:** data processing, dependence measure, relative α-entropy, Rényi divergence, Rényi entropy

## Abstract

Two families of dependence measures between random variables are introduced. They are based on the Rényi divergence of order α and the relative α-entropy, respectively, and both dependence measures reduce to Shannon’s mutual information when their order α is one. The first measure shares many properties with the mutual information, including the data-processing inequality, and can be related to the optimal error exponents in composite hypothesis testing. The second measure does not satisfy the data-processing inequality, but appears naturally in the context of distributed task encoding.

## 1. Introduction

The solutions to many information-theoretic problems can be expressed using Shannon’s information measures such as entropy, relative entropy, and mutual information. Other problems require Rényi’s information measures, which generalize Shannon’s. In this paper, we analyze two Rényi measures of dependence, $J_{\alpha }\left(X;Y\right)$ and $K_{\alpha }\left(X;Y\right)$, between random variables *X* and *Y* taking values in the finite sets X and Y, with α∈[0,∞] being a parameter. (Our notation is similar to the one used for the mutual information: technically, $J_{\alpha }\left(\cdot \right)$ and $K_{\alpha }\left(\cdot \right)$ are functions not of *X* and *Y*, but of their joint probability mass function (PMF) $P_{XY}$.) For α∈[0,∞], we define $J_{\alpha }\left(X;Y\right)$ and $K_{\alpha }\left(X;Y\right)$ as

(1) $$\begin{array}{cc} J_{\alpha }\left(X;Y\right) & ≜\min_{\left(Q_{X},\;Q_{Y}\right)\in P\left(X\right)\times P\left(Y\right)}D_{\alpha }\left(P_{XY}∥Q_{X}Q_{Y}\right), \end{array}$$

(2) $$\begin{array}{cc} K_{\alpha }\left(X;Y\right) & ≜\min_{\left(Q_{X},\;Q_{Y}\right)\in P\left(X\right)\times P\left(Y\right)}\Delta_{\alpha }\left(P_{XY}∥Q_{X}Q_{Y}\right), \end{array}$$

where P(X) and P(Y) denote the set of all PMFs over X and Y, respectively; $D_{\alpha }\left(P∥Q\right)$ denotes the Rényi divergence of order α (see (50) ahead); and $\Delta_{\alpha }\left(P∥Q\right)$ denotes the relative α-entropy (see (55) ahead). As shown in Proposition 7, $J_{\alpha }\left(X;Y\right)$ and $K_{\alpha }\left(X;Y\right)$ are in fact closely related.

The measures $J_{\alpha }\left(X;Y\right)$ and $K_{\alpha }\left(X;Y\right)$ have the following operational meanings (see Section 3): $J_{\alpha }\left(X;Y\right)$ is related to the optimal error exponents in testing whether the observed independent and identically distributed (IID) samples were generated according to the joint PMF $P_{XY}$ or an unknown product PMF; and $K_{\alpha }\left(X;Y\right)$ appears as a penalty term in the sum-rate constraint of distributed task encoding.

**Figure 1:**
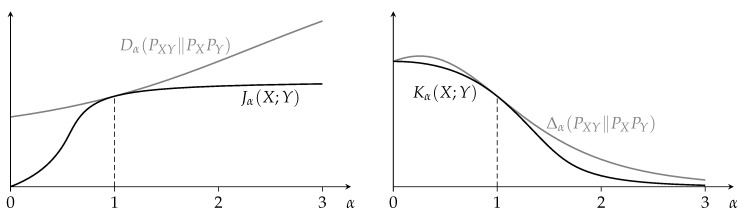
(**Left**) $J_{\alpha }\left(X;Y\right)$ and $D_{\alpha }\left(P_{XY}∥P_{X}P_{Y}\right)$ versus α. (**Right**) $K_{\alpha }\left(X;Y\right)$ and $\Delta_{\alpha }\left(P_{XY}∥P_{X}P_{Y}\right)$ versus α. In both plots, *X* is Bernoulli with Pr(X=1)=0.2, and *Y* is equal to *X*.

The measures $J_{\alpha }\left(X;Y\right)$ and $K_{\alpha }\left(X;Y\right)$ share many properties with Shannon’s mutual information [1], and both are equal to the mutual information when α is one. Except for some special cases, we have no closed-form expressions for $J_{\alpha }\left(X;Y\right)$ or $K_{\alpha }\left(X;Y\right)$. As illustrated in Figure 1, unless α is one, the minimum in the definitions of $J_{\alpha }\left(X;Y\right)$ and $K_{\alpha }\left(X;Y\right)$ is typically not achieved by $Q_{X}=P_{X}$ and $Q_{Y}=P_{Y}$. (When α is one, then the minimum is always achieved by $Q_{X}=P_{X}$ and $Q_{Y}=P_{Y}$; this follows from Proposition 8 and the fact that $D_{1}\left(P_{XY}∥Q_{X}Q_{Y}\right)=\Delta_{1}\left(P_{XY}∥Q_{X}Q_{Y}\right)=D\left(P_{XY}∥Q_{X}Q_{Y}\right)$.)

The rest of this paper is organized as follows. In Section 2, we review other generalizations of the mutual information. In Section 3, we discuss the operational meanings of $J_{\alpha }\left(X;Y\right)$ and $K_{\alpha }\left(X;Y\right)$. In Section 4, we recall the required Rényi information measures and prove some preparatory results. In Section 5, we state the properties of $J_{\alpha }\left(X;Y\right)$ and $K_{\alpha }\left(X;Y\right)$. In Section 6, we prove these properties.

## 2. Related Work

The measure $J_{\alpha }\left(X;Y\right)$ was discovered independently from the authors of the present paper by Tomamichel and Hayashi [2] (Equation (58)), who, for the case when $\alpha >\frac{1}{2}$, derived some of its properties in [2] (Appendix A-C).

Other Rényi-based measures of dependence appeared in the past. Notable are those by Sibson [3], Arimoto [4], and Csiszár [5], respectively denoted by $I_{\alpha }^{s}\left(\cdot \right)$, $I_{\alpha }^{a}\left(\cdot \right)$, and $I_{\alpha }^{c}\left(\cdot \right)$:

(3) $$\begin{array}{cc} I_{\alpha }^{s}\left(X;Y\right) & ≜\frac{\alpha }{\alpha -1}\log\sum_{y}{\left[\sum_{x}P\left(x\right)\;P{\left(y|x\right)}^{\alpha }\right]}^{\frac{1}{\alpha }} \end{array}$$

(4) $$\begin{array}{c} =\min_{Q_{Y}}D_{\alpha }\left(P_{XY}∥P_{X}Q_{Y}\right), \end{array}$$

(5) $$\begin{array}{cc} I_{\alpha }^{a}\left(X;Y\right) & ≜H_{\alpha }\left(X\right)-H_{\alpha }\left(X|Y\right) \end{array}$$

(6) $$\begin{array}{c} =\frac{\alpha }{\alpha -1}\log\sum_{y}{\left[\sum_{x}\frac{P{\left(x\right)}^{\alpha }}{\sum_{x^{\prime }\in X}P{\left(x^{\prime }\right)}^{\alpha }}\;P{\left(y|x\right)}^{\alpha }\right]}^{\frac{1}{\alpha }}, \end{array}$$

(7) $$\begin{array}{cc} I_{\alpha }^{c}\left(X;Y\right) & ≜\min_{Q_{Y}}\sum_{x}P\left(x\right)\;D_{\alpha }\left(P_{Y|X=x}∥Q_{Y}\right), \end{array}$$

where, throughout the paper, log(·) denotes the base-2 logarithm; $D_{\alpha }\left(P∥Q\right)$ denotes the Rényi divergence of order α (see (50) ahead); $H_{\alpha }\left(X\right)$ denotes the Rényi entropy of order α (see (45) ahead); and $H_{\alpha }\left(X|Y\right)$ denotes the Arimoto–Rényi conditional entropy [4,6,7], which is defined for positive α other than one as

(8) $$\begin{array}{c} H_{\alpha }\left(X|Y\right)≜\frac{\alpha }{1-\alpha }\log\sum_{y}{\left[\sum_{x}P{\left(x,y\right)}^{\alpha }\right]}^{\frac{1}{\alpha }}. \end{array}$$

(Equation (4) follows from Proposition 9 ahead, and (6) follows from (45) and (8).) An overview of $I_{\alpha }^{s}\left(\cdot \right)$, $I_{\alpha }^{a}\left(\cdot \right)$, and $I_{\alpha }^{c}\left(\cdot \right)$ is provided in [8]. Another Rényi-based measure of dependence can be found in [9] (Equation (19)):

(9) $$\begin{array}{c} I_{\alpha }^{t}\left(X;Y\right)≜D_{\alpha }\left(P_{XY}∥P_{X}P_{Y}\right). \end{array}$$

The relation between $I_{\alpha }^{c}\left(X;Y\right)$, $J_{\alpha }\left(X;Y\right)$, and $I_{\alpha }^{s}\left(X;Y\right)$ for α>1 was established recently:

**Proposition** **1** ([10] (Theorem IV.1)). *For every PMF $P_{XY}$ and every α>1,*

(10) $$\begin{array}{cc} I_{\alpha }^{c}\left(X;Y\right) & \leq J_{\alpha }\left(X;Y\right) \end{array}$$

(11) $$\begin{array}{c} \leq I_{\alpha }^{s}\left(X;Y\right). \end{array}$$

**Proof.**  This is proved in [10] for a measure-theoretic setting. Here, we specialize the proof to finite alphabets. We first prove (10):

(12) $$\begin{array}{cc} J_{\alpha }\left(X;Y\right) & =\min_{Q_{Y}}\min_{Q_{X}}D_{\alpha }\left(P_{XY}∥Q_{X}Q_{Y}\right) \end{array}$$

(13) $$\begin{array}{c} =\min_{Q_{Y}}\frac{\alpha }{\alpha -1}\log\sum_{x}{\left[\sum_{y}P{\left(x,y\right)}^{\alpha }\;Q_{Y}{\left(y\right)}^{1-\alpha }\right]}^{\frac{1}{\alpha }} \end{array}$$

(14) $$\begin{array}{c} =\min_{Q_{Y}}\frac{\alpha }{\alpha -1}\log\sum_{x}P\left(x\right){\left[\sum_{y}P{\left(y|x\right)}^{\alpha }\;Q_{Y}{\left(y\right)}^{1-\alpha }\right]}^{\frac{1}{\alpha }} \end{array}$$

(15) $$\begin{array}{c} \geq \min_{Q_{Y}}\frac{\alpha }{\alpha -1}\sum_{x}P\left(x\right)\log{\left[\sum_{y}P{\left(y|x\right)}^{\alpha }\;Q_{Y}{\left(y\right)}^{1-\alpha }\right]}^{\frac{1}{\alpha }} \end{array}$$

(16) $$\begin{array}{c} =\min_{Q_{Y}}\sum_{x}P\left(x\right)\frac{1}{\alpha -1}\log\sum_{y}P{\left(y|x\right)}^{\alpha }\;Q_{Y}{\left(y\right)}^{1-\alpha } \end{array}$$

(17) $$\begin{array}{c} =I_{\alpha }^{c}\left(X;Y\right), \end{array}$$

where (12) follows from the definition of $J_{\alpha }\left(X;Y\right)$ in (1); (13) follows from Proposition 9 ahead with the roles of $Q_{X}$ and $Q_{Y}$ swapped; (15) follows from Jensen’s inequality because log(·) is concave and because $\frac{\alpha }{\alpha -1}>0$; and (17) follows from the definition of $I_{\alpha }^{c}\left(X;Y\right)$ in (7).

We next prove (11):

(18) $$\begin{array}{cc} J_{\alpha }\left(X;Y\right) & =\min_{Q_{X},Q_{Y}}D_{\alpha }\left(P_{XY}∥Q_{X}Q_{Y}\right) \end{array}$$

(19) $$\begin{array}{c} \leq \min_{Q_{Y}}D_{\alpha }\left(P_{XY}∥P_{X}Q_{Y}\right) \end{array}$$

(20) $$\begin{array}{c} =I_{\alpha }^{s}\left(X;Y\right), \end{array}$$

where (18) follows from the definition of $J_{\alpha }\left(X;Y\right)$ in (1), and (20) follows from (4). □

Many of the above Rényi information measures coincide when they are maximized over $P_{X}$ with $P_{Y|X}$ held fixed: for every conditional PMF $P_{Y|X}$ and every positive α other than one,

(21) $$\begin{array}{cc} \max_{P_{X}}I_{\alpha }^{a}\left(P_{X}P_{Y|X}\right) & =\max_{P_{X}}I_{\alpha }^{s}\left(P_{X}P_{Y|X}\right) \end{array}$$

(22) $$\begin{array}{c} =\max_{P_{X}}I_{\alpha }^{c}\left(P_{X}P_{Y|X}\right), \end{array}$$

where $P_{X}P_{Y|X}$ denotes the joint PMF of *X* and *Y*; (21) follows from [4] (Lemma 1); and (22) follows from [5] (Proposition 1). It was recently established that, for α>1, this is also true for $J_{\alpha }\left(X;Y\right)$:

**Proposition** **2** ([10] (Theorem V.1)). *For every conditional PMF $P_{Y|X}$ and every α>1,*

(23) $$\begin{array}{c} \max_{P_{X}}J_{\alpha }\left(P_{X}P_{Y|X}\right)=\max_{P_{X}}I_{\alpha }^{s}\left(P_{X}P_{Y|X}\right). \end{array}$$

**Proof.**  By Proposition 1, we have for all α>1

(24) $$\begin{array}{cc} \max_{P_{X}}I_{\alpha }^{c}\left(P_{X}P_{Y|X}\right) & \leq \max_{P_{X}}J_{\alpha }\left(P_{X}P_{Y|X}\right) \end{array}$$

(25) $$\begin{array}{c} \leq \max_{P_{X}}I_{\alpha }^{s}\left(P_{X}P_{Y|X}\right). \end{array}$$

By (22), the left-hand side (LHS) of (24) is equal to the right-hand side (RHS) of (25), so (24) and (25) both hold with equality. □

Dependence measures can also be based on the *f*-divergence $D_{f}\left(P∥Q\right)$ [11,12,13]. Every convex function f:(0,∞)→R satisfying f(1)=0 induces a dependence measure, namely

(26) $$\begin{array}{cc} I_{f}\left(X;Y\right) & ≜D_{f}\left(P_{XY}∥P_{X}P_{Y}\right) \end{array}$$

(27) $$\begin{array}{c} =\sum_{x,y}P\left(x\right)\;P\left(y\right)\;f\left(\frac{P(x,y)}{P(x)\;P(y)}\right), \end{array}$$

where (27) follows from the definition of the *f*-divergence. (For f(t)=tlogt, $I_{f}\left(X;Y\right)$ is the mutual information.) Such dependence measures are used for example in [14], and a construction equivalent to (27) is studied in [15].

## 3. Operational Meanings

In this section, we discuss the operational meaning of $J_{\alpha }\left(X;Y\right)$ in hypothesis testing (Section 3.1) and of $K_{\alpha }\left(X;Y\right)$ in distributed task encoding (Section 3.2).

### 3.1. Testing Against Independence and $J_{\alpha }\left(X;Y\right)$

Consider the hypothesis testing problem of guessing whether an observed sequence of pairs was drawn IID from some given joint PMF $P_{XY}$ or IID from some unknown product distribution. Thus, based on a sequence of pairs of random variables ${\left\{\left(X_{i},Y_{i}\right)\right\}}_{i=1}^{n}$, two hypotheses have to be distinguished:

0) Under the null hypothesis, $\left(X_{1},Y_{1}\right),\ldots ,\left(X_{n},Y_{n}\right)$ are IID according to $P_{XY}$.
1) Under the alternative hypothesis, $\left(X_{1},Y_{1}\right),\ldots ,\left(X_{n},Y_{n}\right)$ are IID according to some unknown PMF of the form $Q_{XY}=Q_{X}Q_{Y}$, where $Q_{X}$ and $Q_{Y}$ are arbitrary PMFs over X and Y, respectively.

Associated with every deterministic test $T_{n}:X^{n}\times Y^{n}\to \left\{0,1\right\}$ and pair $\left(Q_{X},Q_{Y}\right)$ are the type-I error probability $P_{XY}^{\times n}\left[T_{n}\left(X^{n},Y^{n}\right)=1\right]$ and the type-II error probability ${\left(Q_{X}Q_{Y}\right)}^{\times n}\left[T_{n}\left(X^{n},Y^{n}\right)=0\right]$, where $R_{XY}^{\times n}\left[A\right]$ denotes the probability of an event A when ${\left\{\left(X_{i},Y_{i}\right)\right\}}_{i=1}^{n}$ are IID according to $R_{XY}$. We seek sequences of tests whose worst-case type-II error probability decays exponentially faster than $2^{-nE_{Q}}$. To be more specific, for a fixed $E_{Q}\in R$, denote by $T(E_{Q})$ the set of all sequences of deterministic tests ${\left\{T_{n}\right\}}_{n=1}^{\infty }$ for which

(28) $$\begin{array}{c} \underset{n\to \infty }{lim inf}\min_{Q_{X},\;Q_{Y}}-\frac{1}{n}\log\left({\left(Q_{X}Q_{Y}\right)}^{\times n}\left[T_{n}\left(X^{n},Y^{n}\right)=0\right]\right)>E_{Q}, \end{array}$$

where log(·) denotes the base-2 logarithm. Note that (28) implies—but is not equivalent to—that for *n* sufficiently large, ${\left(Q_{X}Q_{Y}\right)}^{\times n}\left[T_{n}\left(X^{n},Y^{n}\right)=0\right]\leq 2^{-n\;E_{Q}}$ for all $\left(Q_{X},Q_{Y}\right)\in P\left(X\right)\times P\left(Y\right)$. For a fixed $E_{Q}\in R$, the optimal type-I error exponent that can be asymptotically achieved under the constraint (28) is given by

(29) $$\begin{array}{c} E_{P}\left(E_{Q}\right)≜\underset{{\left\{T_{n}\right\}}_{n=1}^{\infty }\in T\left(E_{Q}\right)}{\sup}\underset{n\to \infty }{lim inf}-\frac{1}{n}\log\left(P_{XY}^{\times n}\left[T_{n}\left(X^{n},Y^{n}\right)=1\right]\right). \end{array}$$

The measure $J_{\alpha }\left(X;Y\right)$ appears as follows: In [2] (first part of (57)), it is shown that for $E_{Q}$ sufficiently close to I(X;Y),

(30) $$\begin{array}{c} E_{P}\left(E_{Q}\right)=\underset{\alpha \in (\frac{1}{2},1]}{\sup}\frac{1-\alpha }{\alpha }\;\left(J_{\alpha }\left(X;Y\right)-E_{Q}\right), \end{array}$$

and in [16] (Theorem 3), it is shown that for all $E_{Q}\in R$,

(31) $$\begin{array}{c} E_{P}^{**}\left(E_{Q}\right)=\underset{\alpha \in (0,1]}{\sup}\frac{1-\alpha }{\alpha }\;\left(J_{\alpha }\left(X;Y\right)-E_{Q}\right), \end{array}$$

where $E_{P}^{**}\left(\cdot \right)$ denotes the Fenchel biconjugate of $E_{P}\left(\cdot \right)$. In general, the Fenchel biconjugation cannot be omitted because sometimes [16] (Equation (11) and Example 14)

(32) $$\begin{array}{c} E_{P}\left(E_{Q}\right)\neq E_{P}^{**}\left(E_{Q}\right). \end{array}$$

For large values of $E_{Q}$, the optimal type-I error tends to one as *n* tends to infinity. In this case, the type-I strong-converse exponent [17,18], which is defined for a sequence of tests ${\left\{T_{n}\right\}}_{n=1}^{\infty }$ as

(33) $$\begin{array}{c} S\;C_{P}≜\underset{n\to \infty }{lim sup}-\frac{1}{n}\log\left(1-P_{XY}^{\times n}\left[T_{n}\left(X^{n},Y^{n}\right)=1\right]\right), \end{array}$$

measures how fast the type-I error tends to one as *n* tends to infinity (smaller values correspond to lower error probabilities). For a fixed $E_{Q}\in R$, the optimal type-I strong-converse exponent that can be asymptotically achieved under the constraint (28) is given by

(34) $$\begin{array}{c} S\;C_{P}\left(E_{Q}\right)≜\underset{{\left\{T_{n}\right\}}_{n=1}^{\infty }\in T\left(E_{Q}\right)}{\inf}\underset{n\to \infty }{lim sup}-\frac{1}{n}\log\left(1-P_{XY}^{\times n}\left[T_{n}\left(X^{n},Y^{n}\right)=1\right]\right). \end{array}$$

In [2] (second part of (57)), it is shown that for $E_{Q}$ sufficiently close to I(X;Y),

(35) $$\begin{array}{c} S\;C_{P}\left(E_{Q}\right)=\underset{\alpha >1}{\sup}\frac{1-\alpha }{\alpha }\;\left(J_{\alpha }\left(X;Y\right)-E_{Q}\right). \end{array}$$

Here, the same $\frac{1-\alpha }{\alpha }\;\left(J_{\alpha }\left(X;Y\right)-E_{Q}\right)$ expression appears as in (30) and (31), but with a different set of α’s to optimize over.

### 3.2. Distributed Task Encoding and $K_{\alpha }\left(X;Y\right)$

The task-encoding problem studied in [19] can be extended to a distributed setting as follows [20]: A source ${\left\{\left(X_{i},Y_{i}\right)\right\}}_{i=1}^{\infty }$ emits pairs of random variables $\left(X_{i},Y_{i}\right)$ taking values in a finite alphabet X×Y. For a fixed rate pair $\left(R_{X},R_{Y}\right)\in R_{\geq 0}^{2}$ and a positive integer *n*, the sequences ${\left\{X_{i}\right\}}_{i=1}^{n}$ and ${\left\{Y_{i}\right\}}_{i=1}^{n}$ are described separately using $\left\lfloor 2^{nR_{X}}\right\rfloor$ and $\left\lfloor 2^{nR_{Y}}\right\rfloor$ labels, respectively. The decoder produces a list comprising all the pairs $\left(x^{n},y^{n}\right)$ whose description matches the given labels, and the goal is to minimize the ρ-th moment of the list size as *n* tends to infinity (for some ρ>0).

For a fixed ρ>0, a rate pair $\left(R_{X},R_{Y}\right)\in R_{\geq 0}^{2}$ is called achievable if there exists a sequence of encoders ${\left\{\left(f_{n},g_{n}\right)\right\}}_{n=1}^{\infty }$,

(36) $$\begin{array}{cc} f_{n}:X^{n} & \to \{1,\ldots ,\left\lfloor 2^{nR_{X}}\right\rfloor \}, \end{array}$$

(37) $$\begin{array}{cc} g_{n}:Y^{n} & \to \{1,\ldots ,\left\lfloor 2^{nR_{Y}}\right\rfloor \}, \end{array}$$

such that the ρ-th moment of the list size tends to one as *n* tends to infinity, i.e.,

(38) $$\begin{array}{c} \lim_{n\to \infty }E\left[{\left|L\left(X^{n},Y^{n}\right)\right|}^{\rho }\right]=1, \end{array}$$

where

(39) $$\begin{array}{c} L\left(x^{n},y^{n}\right)≜\left\{\left({\tilde{x}}^{n},{\tilde{y}}^{n}\right)\in X^{n}\times Y^{n}:f_{n}\left({\tilde{x}}^{n}\right)=f_{n}\left(x^{n}\right)\;\wedge \;g_{n}\left({\tilde{y}}^{n}\right)=g_{n}\left(y^{n}\right)\right\}. \end{array}$$

For a memoryless source and a fixed ρ>0, rate pairs in the interior of the region R(ρ) defined next are achievable, while those outside R(ρ) are not achievable [20] (Theorem 1). The region R(ρ) is defined as the set of all rate pairs $\left(R_{X},R_{Y}\right)$ satisfying the following inequalities simultaneously:

(40) $$\begin{array}{cc} R_{X} & \geq H_{\frac{1}{1+\rho }}\left(X\right), \end{array}$$

(41) $$\begin{array}{cc} R_{Y} & \geq H_{\frac{1}{1+\rho }}\left(Y\right), \end{array}$$

(42) $$\begin{array}{cc} R_{X}+R_{Y} & \geq H_{\frac{1}{1+\rho }}\left(X,Y\right)+K_{\frac{1}{1+\rho }}\left(X;Y\right), \end{array}$$

where $H_{\alpha }\left(X\right)$ denotes the Rényi entropy of order α (see (45) ahead).

To better understand the role of $K_{\alpha }\left(X;Y\right)$, suppose that the sequences ${\left\{X_{i}\right\}}_{i=1}^{n}$ and ${\left\{Y_{i}\right\}}_{i=1}^{n}$ were allowed to be described jointly using $\left\lfloor 2^{nR_{X}}\right\rfloor \cdot \left\lfloor 2^{nR_{Y}}\right\rfloor \approx 2^{n(R_{X}+R_{Y})}$ labels. Then, by [19] (Theorem I.2), all rate pairs $\left(R_{X},R_{Y}\right)\in R_{\geq 0}^{2}$ satisfying the following inequality with strict inequality would be achievable, while those not satisfying the inequality would not:

(43) $$\begin{array}{c} R_{X}+R_{Y}\geq H_{\frac{1}{1+\rho }}\left(X,Y\right). \end{array}$$

Comparing (42) and (43), we see that the measure $K_{\alpha }\left(X;Y\right)$ appears as a penalty term on the sum-rate constraint incurred by requiring that the sequences be described separately as opposed to jointly.

## 4. Preliminaries

Throughout the paper, log(·) denotes the base-2 logarithm, X and Y are finite sets, $P_{XY}$ denotes a joint PMF over X×Y, $Q_{X}$ denotes a PMF over X, and $Q_{Y}$ denotes a PMF over Y. We use *P* and *Q* as generic PMFs over a finite set X. We denote by supp(P)≜{x∈X:P(x)>0} the support of *P*, and by P(X) the set of all PMFs over X. When clear from the context, we often omit sets and subscripts: for example, we write $\min_{Q_{X},\;Q_{Y}}$ for $\min_{\left(Q_{X},\;Q_{Y}\right)\in P\left(X\right)\times P\left(Y\right)}$, $\sum_{x}$ for $\sum_{x\in X}$, P(x) for $P_{X}\left(x\right)$, and P(y|x) for $P_{Y|X}\left(y|x\right)$. Whenever a conditional probability P(y|x) is undefined because P(x)=0, we define P(y|x)≜1/|Y|. We denote by 𝟙{condition} the indicator function that is one if the condition is satisfied and zero otherwise. In the definitions below, we use the following conventions:

(44) $$\begin{array}{c} \frac{0}{0}=0,\;\frac{p}{0}=\infty \;\forall \;p>0,\;0\log0=0,\;\beta \log0=-\infty \;\forall \;\beta >0. \end{array}$$

The Rényi entropy of order α [21] is defined for positive α other than one as

(45) $$\begin{array}{c} H_{\alpha }\left(X\right)≜\frac{1}{1-\alpha }\log\sum_{x}P{\left(x\right)}^{\alpha }. \end{array}$$

For α being zero, one, or infinity, we define by continuous extension of (45)

(46) $$\begin{array}{cc} H_{0}\left(X\right) & ≜\log\;|\mathrm{supp}(P)|, \end{array}$$

(47) $$\begin{array}{cc} H_{1}\left(X\right) & ≜H(X), \end{array}$$

(48) $$\begin{array}{cc} H_{\infty }\left(X\right) & ≜-\log\max_{x}P\left(x\right), \end{array}$$

where H(X) is the Shannon entropy. With this extension to α∈{0,1,∞}, the Rényi entropy satisfies the following basic properties:

**Proposition** **3** ([5]). *Let P be a PMF. Then,*

*(i)*  For all α∈[0,∞], $H_{\alpha }\left(X\right)\leq \log\;\left|X\right|$. If α∈(0,∞], then $H_{\alpha }\left(X\right)=\log\;\left|X\right|$ if and only if X is distributed uniformly over X.
*(ii)*  The mapping $\alpha \mapsto H_{\alpha }\left(X\right)$ is nonincreasing on [0,∞].
*(iii)*  The mapping $\alpha \mapsto H_{\alpha }\left(X\right)$ is continuous on [0,∞].

The relative entropy (or Kullback–Leibler divergence) is defined as

(49) $$\begin{array}{c} D\left(P∥Q\right)≜\sum_{x}P\left(x\right)\log\frac{P(x)}{Q(x)}. \end{array}$$

The Rényi divergence of order α [21,22] is defined for positive α other than one as

(50) $$\begin{array}{c} D_{\alpha }\left(P∥Q\right)≜\frac{1}{\alpha -1}\log\sum_{x}P{\left(x\right)}^{\alpha }\;Q{\left(x\right)}^{1-\alpha }, \end{array}$$

where we read $P{\left(x\right)}^{\alpha }\;Q{\left(x\right)}^{1-\alpha }$ as $P{\left(x\right)}^{\alpha }\;/Q{\left(x\right)}^{\alpha -1}$ if α>1. For α being zero, one, or infinity, we define by continuous extension of (50)

(51) $$\begin{array}{cc} D_{0}\left(P∥Q\right) & ≜-\log\sum_{x\in \mathrm{supp}(P)}Q\left(x\right), \end{array}$$

(52) $$\begin{array}{cc} D_{1}\left(P∥Q\right) & ≜D(P∥Q), \end{array}$$

(53) $$\begin{array}{cc} D_{\infty }\left(P∥Q\right) & ≜\log\max_{x}\frac{P(x)}{Q(x)}. \end{array}$$

With this extension to α∈{0,1,∞}, the Rényi divergence satisfies the following basic properties:

**Proposition** **4.** *Let P and Q be PMFs. Then,*

*(i)*  *For all α∈[0,1), $D_{\alpha }\left(P∥Q\right)$ is finite if and only if |supp(P)∩supp(Q)|>0. For all α∈[1,∞], $D_{\alpha }\left(P∥Q\right)$ is finite if and only if supp(P)⊆supp(Q).*
*(ii)*  *For all α∈[0,∞], $D_{\alpha }\left(P∥Q\right)\geq 0$. If α∈(0,∞], then $D_{\alpha }\left(P∥Q\right)=0$ if and only if P=Q.*
*(iii)*  *For every α∈[0,∞], the mapping $Q\mapsto D_{\alpha }\left(P∥Q\right)$ is continuous.*
*(iv)*  *The mapping $\alpha \mapsto D_{\alpha }\left(P∥Q\right)$ is nondecreasing on [0,∞].*
*(v)*  *The mapping $\alpha \mapsto D_{\alpha }\left(P∥Q\right)$ is continuous on [0,∞].*

**Proof.**  Part (i) follows from the definition of $D_{\alpha }\left(P∥Q\right)$ and the conventions (44), and Parts (ii)–(v) are shown in [22]. □

The Rényi divergence for negative α is defined as

(54) $$\begin{array}{c} D_{\alpha }\left(P∥Q\right)≜\frac{1}{\alpha -1}\log\sum_{x}\frac{Q{\left(x\right)}^{1-\alpha }}{P{\left(x\right)}^{-\alpha }}. \end{array}$$

(We use negative α only in Lemma 19. More about negative orders can be found in [22] (Section V). For other applications of negative orders, see [23] (Proof of Theorem 1 and Example 1).)

The relative α-entropy [24,25] is defined for positive α other than one as

(55) $$\begin{array}{c} \Delta_{\alpha }\left(P∥Q\right)≜\frac{\alpha }{1-\alpha }\log\sum_{x}P\left(x\right)\;Q{\left(x\right)}^{\alpha -1}+\log\sum_{x}Q{\left(x\right)}^{\alpha }-\frac{1}{1-\alpha }\log\sum_{x}P{\left(x\right)}^{\alpha }, \end{array}$$

where we read $P\left(x\right)\;Q{\left(x\right)}^{\alpha -1}$ as $P\left(x\right)/Q{\left(x\right)}^{1-\alpha }$ if α<1. The relative α-entropy appears in mismatched guessing [26], mismatched source coding [26] (Theorem 8), and mismatched task encoding [19] (Section IV). It also arises in robust parameter estimation and constrained compression settings [25] (Section II). For α being zero, one, or infinity, we define by continuous extension of (55)

(56) $$\begin{array}{cc} \Delta_{0}\left(P∥Q\right) & ≜\left\{\begin{array}{cc} \log\frac{\left|\mathrm{supp}(Q)\right|}{\left|\mathrm{supp}(P)\right|} & \mathrm{if}\;\mathrm{supp}(P)\subseteq \mathrm{supp}(Q),\\ \infty & \mathrm{otherwise}, \end{array}\right. \end{array}$$

(57) $$\begin{array}{cc} \Delta_{1}\left(P∥Q\right) & ≜D(P∥Q), \end{array}$$

(58) $$\begin{array}{cc} \Delta_{\infty }\left(P∥Q\right) & ≜\log\frac{\max_{x}P\left(x\right)}{{\left|\mathrm{argmax}\left(Q\right)\right|}^{-1}\sum_{x\in \mathrm{argmax}(Q)}P\left(x\right)}, \end{array}$$

where $\mathrm{argmax}\left(Q\right)≜\{x\in X:Q\left(x\right)=\max_{x^{\prime }\in X}Q\left(x^{\prime }\right)\}$ and |argmax(Q)| is the cardinality of this set. With this extension to α∈{0,1,∞}, the relative α-entropy satisfies the following basic properties:

**Proposition** **5.** *Let P and Q be PMFs. Then,*

*(i)*  *For all α∈[0,1], $\Delta_{\alpha }\left(P∥Q\right)$ is finite if and only if supp(P)⊆supp(Q). For all α∈(1,∞), $\Delta_{\alpha }\left(P∥Q\right)$ is finite if and only if |supp(P)∩supp(Q)|>0.*
*(ii)*  *For all α∈[0,∞], $\Delta_{\alpha }\left(P∥Q\right)\geq 0$. If α∈(0,∞), then $\Delta_{\alpha }\left(P∥Q\right)=0$ if and only if P=Q.*
*(iii)*  *For every α∈(0,∞), the mapping $Q\mapsto \Delta_{\alpha }\left(P∥Q\right)$ is continuous.*
*(iv)*  *The mapping $\alpha \mapsto \Delta_{\alpha }\left(P∥Q\right)$ is continuous on [0,∞].*

(Part (i) differs from [19] (Proposition IV.1), where the conventions for α>1 differ from ours. Our conventions are compatible with [24,25], and, as stated in Part (iii), they result in the continuity of the mapping $Q\mapsto \Delta_{\alpha }\left(P∥Q\right)$.)

**Proof of** **Proposition 5.** Part (i) follows from the definition of $\Delta_{\alpha }\left(P∥Q\right)$ in (55) and the conventions (44). For α∈(0,1)∪(1,∞), Part (ii) follows from [19] (Proposition IV.1); for α=1, Part (ii) holds because $\Delta_{1}\left(P∥Q\right)=D\left(P∥Q\right)$; and for α∈{0,∞}, Part (ii) follows from the definition of $\Delta_{\alpha }\left(P∥Q\right)$. Part (iii) follows from the definition of $\Delta_{\alpha }\left(P∥Q\right)$, and Part (iv) follows from [19] (Proposition IV.1). □

In the rest of this section, we prove some auxiliary results that we need later (Propositions 6–9). We first establish the relation between $D_{\alpha }\left(P∥Q\right)$ and $\Delta_{\alpha }\left(P∥Q\right)$.

**Proposition** **6** ([26] (Section V, Property 4)). *Let P and Q be PMFs, and let α>0. Then,*

(59) $$\begin{array}{c} \Delta_{\alpha }\left(P∥Q\right)=D_{\frac{1}{\alpha }}\left(\tilde{P}∥\tilde{Q}\right), \end{array}$$

*where the PMFs $\tilde{P}$ and $\tilde{Q}$ are given by*

(60) $$\begin{array}{cc} \tilde{P}\left(x\right) & ≜\frac{P{\left(x\right)}^{\alpha }}{\sum_{x^{\prime }\in X}P{\left(x^{\prime }\right)}^{\alpha }}, \end{array}$$

(61) $$\begin{array}{cc} \tilde{Q}\left(x\right) & ≜\frac{Q{\left(x\right)}^{\alpha }}{\sum_{x^{\prime }\in X}Q{\left(x^{\prime }\right)}^{\alpha }}. \end{array}$$

**Proof.**  If α=1, then (59) holds because $\tilde{P}=P$, $\tilde{Q}=Q$, and $\Delta_{1}\left(P∥Q\right)=D_{1}\left(P∥Q\right)=D\left(P∥Q\right)$. Now let α≠1. Because $\tilde{P}\left(x\right)$ and $\tilde{Q}\left(x\right)$ are zero if and only if P(x) and Q(x) are zero, respectively, the LHS of (59) is finite if and only if its RHS is finite. If $D_{1/\alpha }\left(\tilde{P}∥\tilde{Q}\right)$ is finite, then (59) follows from a simple computation. □

In light of Proposition 6, $J_{\alpha }\left(X;Y\right)$ and $K_{\alpha }\left(X;Y\right)$ are related as follows:

**Proposition** **7.** *Let $P_{XY}$ be a joint PMF, and let α>0. Then,*

(62) $$\begin{array}{c} K_{\alpha }\left(X;Y\right)=J_{\frac{1}{\alpha }}\left(\tilde{X};\tilde{Y}\right), \end{array}$$

*where the joint PMF of $\tilde{X}$ and $\tilde{Y}$ is given by*

(63) $$\begin{array}{c} {\tilde{P}}_{XY}\left(x,y\right)≜\frac{P_{XY}{\left(x,y\right)}^{\alpha }}{\sum_{(x^{\prime },y^{\prime })\in X\times Y}P_{XY}{\left(x^{\prime },y^{\prime }\right)}^{\alpha }}. \end{array}$$

**Proof.**  Let α>0. For fixed PMFs $Q_{X}$ and $Q_{Y}$, define the transformed PMFs $\tilde{Q_{X}Q_{Y}}$, ${\tilde{Q}}_{X}$, and ${\tilde{Q}}_{Y}$ as

(64) $$\begin{array}{cc} \tilde{Q_{X}Q_{Y}}\left(x,y\right) & ≜\frac{{\left[Q_{X}\left(x\right)\;Q_{Y}\left(y\right)\right]}^{\alpha }}{\sum_{(x^{\prime },y^{\prime })\in X\times Y}{\left[Q_{X}\left(x^{\prime }\right)\;Q_{Y}\left(y^{\prime }\right)\right]}^{\alpha }}, \end{array}$$

(65) $$\begin{array}{cc} {\tilde{Q}}_{X}\left(x\right) & ≜\frac{Q_{X}{\left(x\right)}^{\alpha }}{\sum_{x^{\prime }\in X}Q_{X}{\left(x^{\prime }\right)}^{\alpha }}, \end{array}$$

(66) $$\begin{array}{cc} {\tilde{Q}}_{Y}\left(y\right) & ≜\frac{Q_{Y}{\left(y\right)}^{\alpha }}{\sum_{y^{\prime }\in Y}Q_{Y}{\left(y^{\prime }\right)}^{\alpha }}. \end{array}$$

Then,

(67) $$\begin{array}{cc} K_{\alpha }\left(X;Y\right) & =\min_{Q_{X},\;Q_{Y}}\Delta_{\alpha }\left(P_{XY}∥Q_{X}Q_{Y}\right) \end{array}$$

(68) $$\begin{array}{c} =\min_{Q_{X},\;Q_{Y}}D_{\frac{1}{\alpha }}\left({\tilde{P}}_{XY}∥\tilde{Q_{X}Q_{Y}}\right) \end{array}$$

(69) $$\begin{array}{c} =\min_{Q_{X},\;Q_{Y}}D_{\frac{1}{\alpha }}\left({\tilde{P}}_{XY}∥{\tilde{Q}}_{X}{\tilde{Q}}_{Y}\right) \end{array}$$

(70) $$\begin{array}{c} =\min_{Q_{X},\;Q_{Y}}D_{\frac{1}{\alpha }}\left({\tilde{P}}_{XY}∥Q_{X}Q_{Y}\right) \end{array}$$

(71) $$\begin{array}{c} =J_{\frac{1}{\alpha }}\left(\tilde{X};\tilde{Y}\right), \end{array}$$

where (67) holds by the definition of $K_{\alpha }\left(X;Y\right)$; (68) follows from Proposition 6; (69) holds because $\tilde{Q_{X}Q_{Y}}={\tilde{Q}}_{X}{\tilde{Q}}_{Y}$; (70) holds because the transformations (65) and (66) are bijective on the set of PMFs over X and Y, respectively; and (71) holds by the definition of $J_{\alpha }\left(X;Y\right)$. □

The next proposition provides a characterization of the mutual information that parallels the definitions of $J_{\alpha }\left(X;Y\right)$ and $K_{\alpha }\left(X;Y\right)$. Because $D_{1}\left(P∥Q\right)=\Delta_{1}\left(P∥Q\right)=D\left(P∥Q\right)$, this also shows that $J_{\alpha }\left(X;Y\right)$ and $K_{\alpha }\left(X;Y\right)$ reduce to the mutual information when α is one.

**Proposition** **8** ([27] (Theorem 3.4)). *Let $P_{XY}$ be a joint PMF. Then, for all PMFs $Q_{X}$ and $Q_{Y}$,*

(72) $$\begin{array}{c} D\left(P_{XY}∥Q_{X}Q_{Y}\right)\geq D\left(P_{XY}∥P_{X}P_{Y}\right), \end{array}$$

*with equality if and only if $Q_{X}=P_{X}$ and $Q_{Y}=P_{Y}$. Thus,*

(73) $$\begin{array}{c} I\left(X;Y\right)=\min_{Q_{X},\;Q_{Y}}D\left(P_{XY}∥Q_{X}Q_{Y}\right). \end{array}$$

**Proof.**  A simple computation reveals that

(74) $$\begin{array}{c} D\left(P_{XY}∥Q_{X}Q_{Y}\right)=D\left(P_{XY}∥P_{X}P_{Y}\right)+D\left(P_{X}∥Q_{X}\right)+D\left(P_{Y}∥Q_{Y}\right), \end{array}$$

which implies (72) because D(P∥Q)≥0 with equality if and only if P=Q. Thus, (73) holds because $I\left(X;Y\right)=D\left(P_{XY}∥P_{X}P_{Y}\right)$. □

The last proposition of this section is about a precursor to $J_{\alpha }\left(X;Y\right)$, namely, the minimization of $D_{\alpha }\left(P_{XY}∥Q_{X}Q_{Y}\right)$ with respect to $Q_{Y}$ only, which can be carried out explicitly. (This proposition extends [5] (Equation (13)) and [2] (Lemma 29).)

**Proposition** **9.** *Let $P_{XY}$ be a joint PMF and $Q_{X}$ a PMF. Then, for every α∈(0,1)∪(1,∞),*

(75) $$\begin{array}{c} \min_{Q_{Y}}D_{\alpha }\left(P_{XY}∥Q_{X}Q_{Y}\right)=\frac{\alpha }{\alpha -1}\log\sum_{y}{\left[\sum_{x}P{\left(x,y\right)}^{\alpha }\;Q_{X}{\left(x\right)}^{1-\alpha }\right]}^{\frac{1}{\alpha }}, \end{array}$$

*with the conventions of (44). If the RHS of (75) is finite, then the minimum is achieved uniquely by*

(76) $$\begin{array}{c} Q_{Y}^{*}\left(y\right)=\frac{{\left[\sum_{x}P{\left(x,y\right)}^{\alpha }\;Q_{X}{\left(x\right)}^{1-\alpha }\right]}^{\frac{1}{\alpha }}}{\sum_{y^{\prime }\in Y}{\left[\sum_{x}P{\left(x,y^{\prime }\right)}^{\alpha }\;Q_{X}{\left(x\right)}^{1-\alpha }\right]}^{\frac{1}{\alpha }}}. \end{array}$$

*For α=∞,*

(77) $$\begin{array}{c} \min_{Q_{Y}}D_{\infty }\left(P_{XY}∥Q_{X}Q_{Y}\right)=\log\sum_{y}\max_{x}\frac{P(x,y)}{Q_{X}\left(x\right)}, \end{array}$$

*with the conventions of (44). If the RHS of (77) is finite, then the minimum is achieved uniquely by*

(78) $$\begin{array}{c} Q_{Y}^{*}\left(y\right)=\frac{\max_{x}\left[P\left(x,y\right)/Q_{X}\left(x\right)\right]}{\sum_{y^{\prime }\in Y}\max_{x}\left[P\left(x,y^{\prime }\right)/Q_{X}\left(x\right)\right]}. \end{array}$$

**Proof.**  We first treat the case α∈(0,1)∪(1,∞). If the RHS of (75) is infinite, then the conventions imply that $D_{\alpha }\left(P_{XY}∥Q_{X}Q_{Y}\right)$ is infinite for every $Q_{Y}\in P\left(Y\right)$, so (75) holds. Otherwise, if the RHS of (75) is finite, then the PMF $Q_{Y}^{*}$ given by (76) is well-defined, and a simple computation shows that for every $Q_{Y}\in P\left(Y\right)$,

(79) $$\begin{array}{c} D_{\alpha }\left(P_{XY}∥Q_{X}Q_{Y}\right)=\frac{\alpha }{\alpha -1}\log\sum_{y}{\left[\sum_{x}P{\left(x,y\right)}^{\alpha }\;Q_{X}{\left(x\right)}^{1-\alpha }\right]}^{\frac{1}{\alpha }}+D_{\alpha }\left(Q_{Y}^{*}∥Q_{Y}\right). \end{array}$$

The only term on the RHS of (79) that depends on $Q_{Y}$ is $D_{\alpha }\left(Q_{Y}^{*}∥Q_{Y}\right)$. Because $D_{\alpha }\left(Q_{Y}^{*}∥Q_{Y}\right)\geq 0$ with equality if and only if $Q_{Y}=Q_{Y}^{*}$ (Proposition 4), (79) implies (75) and (76).

The case α=∞ is analogous: if the RHS of (77) is infinite, then the LHS of (77) is infinite, too; and if the RHS of (77) is finite, then the PMF $Q_{Y}^{*}$ given by (78) is well-defined, and a simple computation shows that for every $Q_{Y}\in P\left(Y\right)$,

(80) $$\begin{array}{c} D_{\infty }\left(P_{XY}∥Q_{X}Q_{Y}\right)=\log\sum_{y}\max_{x}\frac{P(x,y)}{Q_{X}\left(x\right)}+D_{\infty }\left(Q_{Y}^{*}∥Q_{Y}\right). \end{array}$$

The only term on the RHS of (80) that depends on $Q_{Y}$ is $D_{\infty }\left(Q_{Y}^{*}∥Q_{Y}\right)$. Because $D_{\infty }\left(Q_{Y}^{*}∥Q_{Y}\right)\geq 0$ with equality if and only if $Q_{Y}=Q_{Y}^{*}$ (Proposition 4), (80) implies (77) and (78). □

## 5. Two Measures of Dependence

We state the properties of $J_{\alpha }\left(X;Y\right)$ in Theorem 1 and those of $K_{\alpha }\left(X;Y\right)$ in Theorem 2. The enumeration labels in the theorems refer to the lemmas in Section 6 where the properties are proved. (The enumeration labels are not consecutive because, in order to avoid forward references in the proofs, the order of the results in Section 6 is not the same as here.)

**Theorem** **1.** *Let X, $X_{1}$, $X_{2}$, Y, $Y_{1}$, $Y_{2}$, and Z be random variables taking values in finite sets. Then:*

*(Lemma* *1)* *For every α∈[0,∞], the minimum in the definition of $J_{\alpha }\left(X;Y\right)$ exists and is finite.*

*The following properties of the mutual information I(X;Y) [28] (Chapter 2) are also satisfied by $J_{\alpha }\left(X;Y\right)$:*

*(Lemma* *2)* *For all α∈[0,∞], $J_{\alpha }\left(X;Y\right)\geq 0$. If α∈(0,∞], then $J_{\alpha }\left(X;Y\right)=0$ if and only if X and Y are independent (nonnegativity).*
*(Lemma* *3)* *For all α∈[0,∞], $J_{\alpha }\left(X;Y\right)=J_{\alpha }\left(Y;X\right)$ (symmetry).*
*(Lemma* *4)* *If X⊸−−Y⊸−−Z form a Markov chain, then $J_{\alpha }\left(X;Z\right)\leq J_{\alpha }\left(X;Y\right)$ for all α∈[0,∞] (data-processing inequality).*
*(Lemma* *12)* *If the pairs $\left(X_{1},Y_{1}\right)$ and $\left(X_{2},Y_{2}\right)$ are independent, then $J_{\alpha }\left(X_{1},X_{2};Y_{1},Y_{2}\right)=J_{\alpha }\left(X_{1};Y_{1}\right)+J_{\alpha }\left(X_{2};Y_{2}\right)$ for all α∈[0,∞] (additivity).*
*(Lemma* *13)* *For all α∈[0,∞], $J_{\alpha }\left(X;Y\right)\leq \log\;\left|X\right|$ with equality if and only if $\left(\alpha \in [\frac{1}{2},\infty \right]$, X is distributed uniformly over X, and H(X|Y)=0).*
*(Lemma* *14)* *For every α∈[1,∞], $J_{\alpha }\left(X;Y\right)$ is concave in $P_{X}$ for fixed $P_{Y|X}$.*

*Moreover:*

*(Lemma* *5)* *$J_{0}\left(X;Y\right)=0$.*
*(Lemma* *6)* *Let f:{1,…,|X|}→X and g:{1,…,|Y|}→Y be bijective functions, and let A be the |X|×|Y| matrix whose Row-i Column-j entry $A_{i,j}$ equals $\sqrt{P_{XY}\left(f\left(i\right),g\left(j\right)\right)}$. Then,*
  (81) $$\begin{array}{c} J_{\frac{1}{2}}\left(X;Y\right)=-2\log\sigma_{1}\left(A\right), \end{array}$$
  *where $\sigma_{1}\left(A\right)$ denotes the largest singular value of A. (Because the singular values of a matrix are invariant under row and column permutations, the result does not depend on f or g.)*
*(Lemma* *7)* *$J_{1}\left(X;Y\right)=I\left(X;Y\right)$.*
*(Lemma* *8)* *For all α>0,*
  (82) $$\begin{array}{c} \left(1-\alpha \right)\;J_{\alpha }\left(X;Y\right)=\min_{R_{XY}\in P\left(X\times Y\right)}\left[\left(1-\alpha \right)\;D\left(R_{XY}∥R_{X}R_{Y}\right)+\alpha \;D\left(R_{XY}∥P_{XY}\right)\right]. \end{array}$$
*Thus, being the minimum of concave functions in α, the mapping $\alpha \mapsto \left(1-\alpha \right)\;J_{\alpha }\left(X;Y\right)$ is concave on (0,∞).*
*(Lemma* *9)* *The mapping $\alpha \mapsto J_{\alpha }\left(X;Y\right)$ is nondecreasing on [0,∞].*
*(Lemma* *10)* *The mapping $\alpha \mapsto J_{\alpha }\left(X;Y\right)$ is continuous on [0,∞].*
*(Lemma* *11)* *If X=Y with probability one, then*
  (83) $$\begin{array}{c} J_{\alpha }\left(X;Y\right)=\left\{\begin{array}{cc} \frac{\alpha }{1-\alpha }\;H_{\infty }\left(X\right) & if\;\alpha \in [0,\frac{1}{2}],\\ H_{\frac{\alpha }{2\alpha -1}}\left(X\right) & if\;\alpha >\frac{1}{2},\\ H_{\frac{1}{2}}\left(X\right) & if\;\alpha =\infty . \end{array}\right. \end{array}$$

*The minimization problem in the definition of $J_{\alpha }\left(X;Y\right)$ has the following characteristics:*

*(Lemma* *15)* *For every $\alpha \in [\frac{1}{2},\infty ]$, the mapping $\left(Q_{X},Q_{Y}\right)\mapsto D_{\alpha }\left(P_{XY}∥Q_{X}Q_{Y}\right)$ is convex, i.e., for all $\lambda ,\lambda^{\prime }\in \left[0,1\right]$ with $\lambda +\lambda^{\prime }=1$, all $Q_{X},Q_{X}^{\prime }\in P\left(X\right)$, and all $Q_{Y},Q_{Y}^{\prime }\in P\left(Y\right)$,*
  (84) $$\begin{array}{c} D_{\alpha }\left(P_{XY}∥\left(\lambda Q_{X}+\lambda^{\prime }Q_{X}^{\prime }\right)\left(\lambda Q_{Y}+\lambda^{\prime }Q_{Y}^{\prime }\right)\right)\leq \lambda \;D_{\alpha }\left(P_{XY}∥Q_{X}Q_{Y}\right)+\lambda^{\prime }\;D_{\alpha }\left(P_{XY}∥Q_{X}^{\prime }Q_{Y}^{\prime }\right). \end{array}$$
*For $\alpha \in [0,\frac{1}{2})$, the mapping need not be convex.*
*(Lemma* *16)* Let α∈(0,1)∪(1,∞). If $\left(Q_{X}^{*},Q_{Y}^{*}\right)$ achieves the minimum in the definition of $J_{\alpha }\left(X;Y\right)$, then there exist positive normalization constants c and d such that
  (85) $$\begin{array}{cc} Q_{X}^{*}\left(x\right) & =c{\left[\sum_{y}P{\left(x,y\right)}^{\alpha }\;Q_{Y}^{*}{\left(y\right)}^{1-\alpha }\right]}^{\frac{1}{\alpha }}\;\forall \;x\in X, \end{array}$$
  (86) $$\begin{array}{cc} Q_{Y}^{*}\left(y\right) & =d{\left[\sum_{x}P{\left(x,y\right)}^{\alpha }\;Q_{X}^{*}{\left(x\right)}^{1-\alpha }\right]}^{\frac{1}{\alpha }}\;\forall \;y\in Y, \end{array}$$
  with the conventions of (44). The case α=∞ is similar: if $\left(Q_{X}^{*},Q_{Y}^{*}\right)$ achieves the minimum in the definition of $J_{\infty }\left(X;Y\right)$, then there exist positive normalization constants c and d such that
  (87) $$\begin{array}{cc} Q_{X}^{*}\left(x\right) & =c\max_{y}\frac{P(x,y)}{Q_{Y}^{*}\left(y\right)}\;\forall \;x\in X, \end{array}$$
  (88) $$\begin{array}{cc} Q_{Y}^{*}\left(y\right) & =d\max_{x}\frac{P(x,y)}{Q_{X}^{*}\left(x\right)}\;\forall \;y\in Y, \end{array}$$
  with the conventions of (44). (If α=1, then $Q_{X}^{*}=P_{X}$ and $Q_{Y}^{*}=P_{Y}$ by Proposition 8.) Thus, for all α∈(0,∞], both inclusions $\mathrm{supp}\left(Q_{X}^{*}\right)\subseteq \mathrm{supp}\left(P_{X}\right)$ and $\mathrm{supp}\left(Q_{Y}^{*}\right)\subseteq \mathrm{supp}\left(P_{Y}\right)$ hold. $\frac{1}{2}$
*(Lemma* *20)* *For every $\alpha \in (\frac{1}{2},\infty ]$, the mapping $\left(Q_{X},Q_{Y}\right)\mapsto D_{\alpha }\left(P_{XY}∥Q_{X}Q_{Y}\right)$ has a unique minimizer. This need not be the case when $\alpha \in [0,\frac{1}{2}]$.*

*The measure $J_{\alpha }\left(X;Y\right)$ can also be expressed as follows:*

*(Lemma* *17)* *For all α∈(0,∞],*
  (89) $$\begin{array}{c} J_{\alpha }\left(X;Y\right)=\min_{Q_{X}}\phi_{\alpha }\left(Q_{X}\right), \end{array}$$
  *where $\phi_{\alpha }\left(Q_{X}\right)$ is defined as*
  (90) $$\begin{array}{c} \phi_{\alpha }\left(Q_{X}\right)≜\min_{Q_{Y}}D_{\alpha }\left(P_{XY}∥Q_{X}Q_{Y}\right) \end{array}$$
  *and is given explicitly as follows: for α∈(0,1)∪(1,∞),*
  (91) $$\begin{array}{c} \phi_{\alpha }\left(Q_{X}\right)=\frac{\alpha }{\alpha -1}\log\sum_{y}{\left[\sum_{x}P{\left(x,y\right)}^{\alpha }\;Q_{X}{\left(x\right)}^{1-\alpha }\right]}^{\frac{1}{\alpha }}, \end{array}$$
  *with the conventions of (44); and for α∈{1,∞},*
  (92) $$\begin{array}{cc} \phi_{1}\left(Q_{X}\right) & =D(P_{XY}∥Q_{X}P_{Y}), \end{array}$$
  (93) $$\begin{array}{cc} \phi_{\infty }\left(Q_{X}\right) & =\log\sum_{y}\max_{x}\frac{P(x,y)}{Q_{X}\left(x\right)}, \end{array}$$
  *with the conventions of (44). For every $\alpha \in [\frac{1}{2},\infty ]$, the mapping $Q_{X}\mapsto \phi_{\alpha }\left(Q_{X}\right)$ is convex. For $\alpha \in (0,\frac{1}{2})$, the mapping need not be convex.*
*(Lemma* *18)* *For all α∈(0,1)∪(1,∞],*
  (94) $$\begin{array}{c} J_{\alpha }\left(X;Y\right)=\left\{\begin{array}{cc} \min_{R_{XY}\in P\left(X\times Y\right)}\psi_{\alpha }\left(R_{XY}\right) & if\;\alpha \in (0,1),\\ \max_{R_{XY}\in P\left(X\times Y\right)}\psi_{\alpha }\left(R_{XY}\right) & if\;\alpha \in (1,\infty ], \end{array}\right. \end{array}$$
  *where*
  (95) $$\begin{array}{c} \psi_{\alpha }\left(R_{XY}\right)≜\left\{\begin{array}{cc} D\left(R_{XY}∥R_{X}R_{Y}\right)+\frac{\alpha }{1-\alpha }D\left(R_{XY}∥P_{XY}\right) & if\;\alpha \in (0,1)\cup (1,\infty ),\\ D\left(R_{XY}∥R_{X}R_{Y}\right)-D\left(R_{XY}∥P_{XY}\right) & if\;\alpha =\infty . \end{array}\right. \end{array}$$
*For every α∈(1,∞], the mapping $R_{XY}\mapsto \psi_{\alpha }\left(R_{XY}\right)$ is concave. For all α∈(1,∞] and all $R_{XY}\in P\left(X\times Y\right)$, the statement $J_{\alpha }\left(X;Y\right)=\psi_{\alpha }\left(R_{XY}\right)$ is equivalent to $\psi_{\alpha }\left(R_{XY}\right)=D_{\alpha }\left(P_{XY}∥R_{X}R_{Y}\right)$.*
*(Lemma* *19)* *For all α∈(0,1)∪(1,∞),*
  (96) $$\begin{array}{c} J_{\alpha }\left(X;Y\right)=\min_{R_{X}\ll P_{X}}\frac{1}{\alpha -1}\left[D_{\frac{\alpha }{\alpha -1}}\left(P_{X}∥R_{X}\right)-\alpha \;E_{0}\left(\frac{1-\alpha }{\alpha },R_{X}\right)\right], \end{array}$$
  *where the minimization is over all PMFs $R_{X}$ satisfying $R_{X}\ll P_{X}$$\left(i.e.,\;\mathrm{supp}\left(R_{X}\right)\subseteq \mathrm{supp}\left(P_{X}\right)\right)$; $D_{\alpha }\left(P∥Q\right)$ for negative α is given by (54); and Gallager’s $E_{0}$ function [29] is defined as*
  (97) $$\begin{array}{c} E_{0}\left(\rho ,R_{X}\right)≜-\log\sum_{y}{\left[\sum_{x}R_{X}\left(x\right)\;P{\left(y|x\right)}^{\frac{1}{1+\rho }}\right]}^{1+\rho }. \end{array}$$

We now move on to the properties of $K_{\alpha }\left(X;Y\right)$. Some of these properties are derived from their counterparts of $J_{\alpha }\left(X;Y\right)$ using the relation $K_{\alpha }\left(X;Y\right)=J_{1/\alpha }\left(\tilde{X};\tilde{Y}\right)$ described in Proposition 7.

**Theorem** **2.** *Let X, $X_{1}$, $X_{2}$, Y, $Y_{1}$, $Y_{2}$, and Z be random variables taking values in finite sets. Then:*

*(Lemma* *21)* *For every α∈[0,∞], the minimum in the definition of $K_{\alpha }\left(X;Y\right)$ in (2) exists and is finite.*

*The following properties of the mutual information I(X;Y) are also satisfied by $K_{\alpha }\left(X;Y\right)$:*

*(Lemma* *22)* *For all α∈[0,∞], $K_{\alpha }\left(X;Y\right)\geq 0$. If α∈(0,∞), then $K_{\alpha }\left(X;Y\right)=0$ if and only if X and Y are independent (nonnegativity).*
*(Lemma* *23)* *For all α∈[0,∞], $K_{\alpha }\left(X;Y\right)=K_{\alpha }\left(Y;X\right)$ (symmetry).*
*(Lemma* *34)* *If the pairs $\left(X_{1},Y_{1}\right)$ and $\left(X_{2},Y_{2}\right)$ are independent, then $K_{\alpha }\left(X_{1},X_{2};Y_{1},Y_{2}\right)=K_{\alpha }\left(X_{1};Y_{1}\right)+K_{\alpha }\left(X_{2};Y_{2}\right)$ for all α∈[0,∞] (additivity).*
*(Lemma* *35)* *For all α∈[0,∞], $K_{\alpha }\left(X;Y\right)\leq \log\;\left|X\right|$.*

*Unlike the mutual information, $K_{\alpha }\left(X;Y\right)$ does not satisfy the data-processing inequality:*

*(Lemma* *36)* *There exists a Markov chain X⊸−−Y⊸−−Z for which $K_{2}\left(X;Z\right)>K_{2}\left(X;Y\right)$.*

*Moreover:*

*(Lemma* *24)* *For all α∈(0,∞),*
  (98) $$\begin{array}{c} K_{\alpha }\left(X;Y\right)+H_{\alpha }\left(X,Y\right)=\min_{Q_{X},\;Q_{Y}}-\log M_{\frac{\alpha -1}{\alpha }}\left(Q_{X},Q_{Y}\right), \end{array}$$
  *where $M_{\beta }\left(Q_{X},Q_{Y}\right)$ is the following weighted power mean [30] (Chapter III): For β∈R∖{0},*
  (99) $$\begin{array}{c} M_{\beta }\left(Q_{X},Q_{Y}\right)≜{\left[\sum_{x,y}P\left(x,y\right){\left[Q_{X}\left(x\right)\;Q_{Y}\left(y\right)\right]}^{\beta }\right]}^{\frac{1}{\beta }}, \end{array}$$
  *where for β<0, we read $P\left(x,y\right){\left[Q_{X}\left(x\right)\;Q_{Y}\left(y\right)\right]}^{\beta }$ as $P\left(x,y\right)/{\left[Q_{X}\left(x\right)\;Q_{Y}\left(y\right)\right]}^{-\beta }$ and use the conventions (44); and for β=0, using the convention $0^{0}=1$,*
  (100) $$\begin{array}{c} M_{0}\left(Q_{X},Q_{Y}\right)≜\prod_{x,y}{\left[Q_{X}\left(x\right)\;Q_{Y}\left(y\right)\right]}^{P(x,y)}. \end{array}$$
*(Lemma* *25)* *For α=0,*
  (101) $$\begin{array}{cc} K_{0}\left(X;Y\right) & =\log\frac{\left|\mathrm{supp}\left(P_{X}P_{Y}\right)\right|}{\left|\mathrm{supp}\left(P_{XY}\right)\right|} \end{array}$$
  (102) $$\begin{array}{c} \geq \min_{Q_{X},\;Q_{Y}}\log\max_{\left(x,y\right)\in \mathrm{supp}\left(P_{XY}\right)}\frac{1}{Q_{X}\left(x\right)\;Q_{Y}\left(y\right)}-\log\;\left|\mathrm{supp}\left(P_{XY}\right)\right| \end{array}$$
  (103) $$\begin{array}{c} =\lim_{\alpha ↓0}K_{\alpha }\left(X;Y\right), \end{array}$$
  *where in the RHS of (102), we use the conventions (44). The inequality can be strict, so $\alpha \mapsto K_{\alpha }\left(X;Y\right)$ need not be continuous at α=0.*
*(Lemma* *26)* *$K_{1}\left(X;Y\right)=I\left(X;Y\right)$.*
*(Lemma* *27)* *Let f:{1,…,|X|}→X and g:{1,…,|Y|}→Y be bijective functions, and let B be the |X|×|Y| matrix whose Row-i Column-j entry $B_{i,j}$ equals $P_{XY}\left(f\left(i\right),g\left(j\right)\right)$. Then,*
  (104) $$\begin{array}{c} K_{2}\left(X;Y\right)=-2\log\sigma_{1}\left(B\right)-H_{2}\left(X,Y\right), \end{array}$$
  *where $\sigma_{1}\left(B\right)$ denotes the largest singular value of B. (Because the singular values of a matrix are invariant under row and column permutations, the result does not depend on f or g.)*
*(Lemma* *28)* *$K_{\infty }\left(X;Y\right)=0$.*
*(Lemma* *29)* *The mapping $\alpha \mapsto K_{\alpha }\left(X;Y\right)$ need not be monotonic on [0,∞].*
*(Lemma* *30)* *The mapping $\alpha \mapsto K_{\alpha }\left(X;Y\right)+H_{\alpha }\left(X,Y\right)$ is nonincreasing on [0,∞].*
*(Lemma* *31)* *The mapping $\alpha \mapsto K_{\alpha }\left(X;Y\right)$ is continuous on (0,∞]. (See Lemma 25 for the behavior at α=0.)*
*(Lemma* *32)* *If X=Y with probability one, then*
  (105) $$\begin{array}{c} K_{\alpha }\left(X;Y\right)=\left\{\begin{array}{cc} 2H_{\frac{\alpha }{2-\alpha }}\left(X\right)-H_{\alpha }\left(X\right) & if\;\alpha \in [0,2),\\ \frac{\alpha }{\alpha -1}\;H_{\infty }\left(X\right)-H_{\alpha }\left(X\right) & if\;\alpha \geq 2,\\ 0 & if\;\alpha =\infty . \end{array}\right. \end{array}$$
*(Lemma* *33)* *For every α∈(0,2), the mapping $\left(Q_{X},Q_{Y}\right)\mapsto \Delta_{\alpha }\left(P_{XY}∥Q_{X}Q_{Y}\right)$ in the definition of $K_{\alpha }\left(X;Y\right)$ in (2) has a unique minimizer. This need not be the case when α∈{0}∪[2,∞].*

## 6. Proofs

In this section, we prove the properties of $J_{\alpha }\left(X;Y\right)$ and $K_{\alpha }\left(X;Y\right)$ stated in Section 5.

**Lemma** **1.** *For every α∈[0,∞], the minimum in the definition of $J_{\alpha }\left(X;Y\right)$ exists and is finite.*

**Proof.**  Let α∈[0,∞]. Then $\inf_{Q_{X},\;Q_{Y}}D_{\alpha }\left(P_{XY}∥Q_{X}Q_{Y}\right)$ is finite because $D_{\alpha }\left(P_{XY}∥P_{X}P_{Y}\right)$ is finite and because the Rényi divergence is nonnegative. The minimum exists because the set P(X)×P(Y) is compact and the mapping $\left(Q_{X},Q_{Y}\right)\mapsto D_{\alpha }\left(P_{XY}∥Q_{X}Q_{Y}\right)$ is continuous. □

**Lemma** **2.** *For all α∈[0,∞], $J_{\alpha }\left(X;Y\right)\geq 0$. If α∈(0,∞], then $J_{\alpha }\left(X;Y\right)=0$ if and only if X and Y are independent (nonnegativity).*

**Proof.**  The nonnegativity follows from the definition of $J_{\alpha }\left(X;Y\right)$ because the Rényi divergence is nonnegative for α∈[0,∞]. If *X* and *Y* are independent, then $P_{XY}=P_{X}P_{Y}$, and the choice $Q_{X}=P_{X}$ and $Q_{Y}=P_{Y}$ in the definition of $J_{\alpha }\left(X;Y\right)$ achieves $J_{\alpha }\left(X;Y\right)=0$. Conversely, if $J_{\alpha }\left(X;Y\right)=0$, then there exist PMFs $Q_{X}^{*}$ and $Q_{Y}^{*}$ satisfying $D_{\alpha }\left(P_{XY}∥Q_{X}^{*}Q_{Y}^{*}\right)=0$. If, in addition, α∈(0,∞], then $P_{XY}=Q_{X}^{*}Q_{Y}^{*}$ by Proposition 4, and hence *X* and *Y* are independent. □

**Lemma** **3.** *For all α∈[0,∞], $J_{\alpha }\left(X;Y\right)=J_{\alpha }\left(Y;X\right)$ (symmetry).*

**Proof.**  The definition of $J_{\alpha }\left(X;Y\right)$ is symmetric in *X* and *Y*. □

**Lemma** **4.** *If X⊸−−Y⊸−−Z form a Markov chain, then $J_{\alpha }\left(X;Z\right)\leq J_{\alpha }\left(X;Y\right)$ for all α∈[0,∞] (data-processing inequality).*

**Proof.**  Let X⊸−−Y⊸−−Z form a Markov chain, and let α∈[0,∞]. Let ${\hat{Q}}_{X}$ and ${\hat{Q}}_{Y}$ be PMFs that achieve the minimum in the definition of $J_{\alpha }\left(X;Y\right)$, so

(106) $$\begin{array}{c} J_{\alpha }\left(X;Y\right)=D_{\alpha }\left(P_{XY}∥{\hat{Q}}_{X}{\hat{Q}}_{Y}\right). \end{array}$$

Define the PMF ${\hat{Q}}_{Z}$ as

(107) $$\begin{array}{c} {\hat{Q}}_{Z}\left(z\right)≜\sum_{y}{\hat{Q}}_{Y}\left(y\right)\;P_{Z|Y}\left(z|y\right). \end{array}$$

(As noted in the preliminaries, we define $P_{Z|Y}\left(z|y\right)≜1/\left|Z\right|$ when $P_{Y}\left(y\right)=0$.) We show below that

(108) $$\begin{array}{c} D_{\alpha }\left(P_{XZ}∥{\hat{Q}}_{X}{\hat{Q}}_{Z}\right)\leq D_{\alpha }\left(P_{XY}∥{\hat{Q}}_{X}{\hat{Q}}_{Y}\right), \end{array}$$

which implies the data-processing inequality because

(109) $$\begin{array}{cc} J_{\alpha }\left(X;Z\right) & \leq D_{\alpha }\left(P_{XZ}∥{\hat{Q}}_{X}{\hat{Q}}_{Z}\right) \end{array}$$

(110) $$\begin{array}{c} \leq D_{\alpha }\left(P_{XY}∥{\hat{Q}}_{X}{\hat{Q}}_{Y}\right) \end{array}$$

(111) $$\begin{array}{c} =J_{\alpha }\left(X;Y\right), \end{array}$$

where (109) holds by the definition of $J_{\alpha }\left(X;Z\right)$; (110) follows from (108); and (111) follows from (106).

The proof of (108) is based on the data-processing inequality for the Rényi divergence. Define the conditional PMF $A_{X^{\prime }Z^{\prime }|XY}$ as

(112) $$\begin{array}{c} A_{X^{\prime }Z^{\prime }|XY}\left(x^{\prime },z^{\prime }|x,y\right)≜𝟙\left\{x^{\prime }=x\right\}\;P_{Z|Y}\left(z^{\prime }|y\right). \end{array}$$

If $\left(X,Y\right)∼P_{XY}$, then the marginal distribution of $X^{\prime }$ and $Z^{\prime }$ is

(113) $$\begin{array}{cc} \left(P_{XY}A_{X^{\prime }Z^{\prime }|XY}\right)\left(x^{\prime },z^{\prime }\right) & =\sum_{x,y}P_{XY}\left(x,y\right)\;A_{X^{\prime }Z^{\prime }|XY}\left(x^{\prime },z^{\prime }|x,y\right) \end{array}$$

(114) $$\begin{array}{c} =\sum_{y}P_{XY}\left(x^{\prime },y\right)\;P_{Z|Y}\left(z^{\prime }|y\right) \end{array}$$

(115) $$\begin{array}{c} =\sum_{y}P_{XY}\left(x^{\prime },y\right)\;P_{Z|XY}\left(z^{\prime }|x^{\prime },y\right) \end{array}$$

(116) $$\begin{array}{c} =P_{XZ}\left(x^{\prime },z^{\prime }\right), \end{array}$$

where (114) follows from (112); and (115) holds because *X*, *Y*, and *Z* form a Markov chain. If $\left(X,Y\right)∼{\hat{Q}}_{X}{\hat{Q}}_{Y}$, then the marginal distribution of $X^{\prime }$ and $Z^{\prime }$ is

(117) $$\begin{array}{cc} \left({\hat{Q}}_{X}{\hat{Q}}_{Y}A_{X^{\prime }Z^{\prime }|XY}\right)\left(x^{\prime },z^{\prime }\right) & =\sum_{x,y}{\hat{Q}}_{X}\left(x\right)\;{\hat{Q}}_{Y}\left(y\right)\;A_{X^{\prime }Z^{\prime }|XY}\left(x^{\prime },z^{\prime }|x,y\right) \end{array}$$

(118) $$\begin{array}{c} =\sum_{y}{\hat{Q}}_{X}\left(x^{\prime }\right)\;{\hat{Q}}_{Y}\left(y\right)\;P_{Z|Y}\left(z^{\prime }|y\right) \end{array}$$

(119) $$\begin{array}{c} ={\hat{Q}}_{X}\left(x^{\prime }\right)\;{\hat{Q}}_{Z}\left(z^{\prime }\right), \end{array}$$

where (118) follows from (112), and (119) follows from (107). Finally, we are ready to prove (108):

(120) $$\begin{array}{cc} D_{\alpha }\left(P_{XZ}∥{\hat{Q}}_{X}{\hat{Q}}_{Z}\right) & =D_{\alpha }\left(\left(P_{XY}A_{X^{\prime }Z^{\prime }|XY}\right)∥\left({\hat{Q}}_{X}{\hat{Q}}_{Y}A_{X^{\prime }Z^{\prime }|XY}\right)\right) \end{array}$$

(121) $$\begin{array}{c} \leq D_{\alpha }\left(P_{XY}∥{\hat{Q}}_{X}{\hat{Q}}_{Y}\right), \end{array}$$

where (120) follows from (116) and (119), and where (121) follows from the data-processing inequality for the Rényi divergence [22] (Theorem 9). □

**Lemma** **5.** *$J_{0}\left(X;Y\right)=0$.*

**Proof.**  By Lemma 2, $J_{0}\left(X;Y\right)\geq 0$, so it suffices to show that $J_{0}\left(X;Y\right)\leq 0$. Let $(\hat{x},\hat{y})\in X\times Y$ satisfy $P_{XY}\left(\hat{x},\hat{y}\right)>0$. Define the PMF ${\hat{Q}}_{X}$ as ${\hat{Q}}_{X}\left(x\right)≜𝟙\left\{x=\hat{x}\right\}$ and the PMF ${\hat{Q}}_{Y}$ as ${\hat{Q}}_{Y}\left(y\right)≜𝟙\left\{y=\hat{y}\right\}$. Then, $D_{0}\left(P_{XY}∥{\hat{Q}}_{X}{\hat{Q}}_{Y}\right)=0$, so $J_{0}\left(X;Y\right)\leq 0$ by the definition of $J_{0}\left(X;Y\right)$. □

**Lemma** **6.** *Let f:{1,…,|X|}→X and g:{1,…,|Y|}→Y be bijective functions, and let A be the |X|×|Y| matrix whose Row-i Column-j entry $A_{i,j}$ equals $\sqrt{P_{XY}\left(f\left(i\right),g\left(j\right)\right)}$. Then,*

(122) $$\begin{array}{c} J_{\frac{1}{2}}\left(X;Y\right)=-2\log\sigma_{1}\left(A\right), \end{array}$$

*where $\sigma_{1}\left(A\right)$ denotes the largest singular value of A. (Because the singular values of a matrix are invariant under row and column permutations, the result does not depend on f or g.)*

**Proof.**  By the definitions of $J_{\alpha }\left(X;Y\right)$ and the Rényi divergence,

(123) $$\begin{array}{c} J_{\frac{1}{2}}\left(X;Y\right)=-2\log\max_{Q_{X},\;Q_{Y}}\sum_{x,y}\sqrt{Q_{X}\left(x\right)}\;\sqrt{P(x,y)}\;\sqrt{Q_{Y}\left(y\right)}. \end{array}$$

The claim follows from (123) because

(124) $$\begin{array}{cc} \max_{Q_{X},\;Q_{Y}}\sum_{x,y}\sqrt{Q_{X}\left(x\right)}\;\sqrt{P(x,y)}\;\sqrt{Q_{Y}\left(y\right)} & =\max_{{∥u∥}_{2}={∥v∥}_{2}=1}u^{T}\;A\;v \end{array}$$

(125) $$\begin{array}{c} =\max_{{∥v∥}_{2}=1}{∥A\;v∥}_{2} \end{array}$$

(126) $$\begin{array}{c} =\sigma_{1}\left(A\right), \end{array}$$

where u and v are column vectors with |X| and |Y| elements, respectively; (124) is shown below; (125) follows from the Cauchy–Schwarz inequality $|u^{T}\;A\;v{|\leq ∥u∥}_{2}\;{∥A\;v∥}_{2}$, which holds with equality if u and Av are linearly dependent; and (126) holds because the spectral norm of a matrix is equal to its largest singular value [31] (Example 5.6.6).

We now prove (124). Let u and v be vectors that satisfy ${∥u∥}_{2}={∥v∥}_{2}=1$, and define the PMFs ${\hat{Q}}_{X}$ and ${\hat{Q}}_{Y}$ as ${\hat{Q}}_{X}\left(x\right)≜u_{f^{-1}\left(x\right)}^{2}$ and ${\hat{Q}}_{Y}\left(y\right)≜v_{g^{-1}\left(y\right)}^{2}$, where $f^{-1}$ and $g^{-1}$ denote the inverse functions of *f* and *g*, respectively. Then,

(127) $$\begin{array}{cc} u^{T}\;A\;v & =\sum_{i,j}u_{i}\;A_{i,j}\;v_{j} \end{array}$$

(128) $$\begin{array}{c} \leq \sum_{i,j}|u_{i}|\;A_{i,j}\;\left|v_{j}\right| \end{array}$$

(129) $$\begin{array}{c} =\sum_{x,y}\sqrt{{\hat{Q}}_{X}\left(x\right)}\;\sqrt{P(x,y)}\;\sqrt{{\hat{Q}}_{Y}\left(y\right)} \end{array}$$

(130) $$\begin{array}{c} \leq \max_{Q_{X},\;Q_{Y}}\sum_{x,y}\sqrt{Q_{X}\left(x\right)}\;\sqrt{P(x,y)}\;\sqrt{Q_{Y}\left(y\right)}, \end{array}$$

where (128) holds because all the entries of A are nonnegative, and in (129), we changed the summation variables to x≜f(i) and y≜g(j). It remains to show that equality can be achieved in (128) and (130). To that end, let $Q_{X}^{*}$ and $Q_{Y}^{*}$ be PMFs that achieve the maximum on the RHS of (130), and define the vectors u and v as $u_{i}≜Q_{X}^{*}{\left(f\left(i\right)\right)}^{1/2}$ and $v_{j}≜Q_{Y}^{*}{\left(g\left(j\right)\right)}^{1/2}$. Then, ${∥u∥}_{2}={∥v∥}_{2}=1$, and (128) and (130) hold with equality, which proves (124). □

**Lemma** **7.** *$J_{1}\left(X;Y\right)=I\left(X;Y\right)$.*

**Proof.**  This follows from Proposition 8 because $D_{1}\left(P_{XY}∥Q_{X}Q_{Y}\right)$ in the definition of $J_{1}\left(X;Y\right)$ is equal to $D(P_{XY}∥Q_{X}Q_{Y})$. □

**Lemma** **8.** *For all α>0,*

(131) $$\begin{array}{c} \left(1-\alpha \right)\;J_{\alpha }\left(X;Y\right)=\min_{R_{XY}\in P\left(X\times Y\right)}\left[\left(1-\alpha \right)\;D\left(R_{XY}∥R_{X}R_{Y}\right)+\alpha \;D\left(R_{XY}∥P_{XY}\right)\right]. \end{array}$$

*Thus, being the minimum of concave functions in α, the mapping $\alpha \mapsto \left(1-\alpha \right)\;J_{\alpha }\left(X;Y\right)$ is concave on (0,∞).*

**Proof.**  For α=1, (131) holds because $D(R_{XY}∥P_{XY})\geq 0$ with equality if $R_{XY}=P_{XY}$. For α∈(0,1),

(132) $$\begin{array}{cc} \left(1-\alpha \right)\;J_{\alpha }\left(X;Y\right) & =\min_{Q_{X},\;Q_{Y}}\left(1-\alpha \right)\;D_{\alpha }\left(P_{XY}∥Q_{X}Q_{Y}\right) \end{array}$$

(133) $$\begin{array}{c} =\min_{Q_{X},\;Q_{Y}}\min_{R_{XY}}\;\left[\left(1-\alpha \right)\;D\left(R_{XY}∥Q_{X}Q_{Y}\right)+\alpha \;D\left(R_{XY}∥P_{XY}\right)\right] \end{array}$$

(134) $$\begin{array}{c} =\min_{R_{XY}}\;\left[\left(1-\alpha \right)\;D\left(R_{XY}∥R_{X}R_{Y}\right)+\alpha \;D\left(R_{XY}∥P_{XY}\right)\right], \end{array}$$

where (132) holds by the definition of $J_{\alpha }\left(X;Y\right)$; (133) follows from [22] (Theorem 30); and (134) follows from Proposition 8 after swapping the minima.

For α>1, define the sets

(135) $$\begin{array}{cc} Q & ≜\{\left(Q_{X},Q_{Y}\right)\in P\left(X\right)\times P\left(Y\right):\mathrm{supp}\left(Q_{X}Q_{Y}\right)=X\times Y\}, \end{array}$$

(136) $$\begin{array}{cc} R & ≜\{R_{XY}\in P\left(X\times Y\right):\mathrm{supp}\left(R_{XY}\right)\subseteq \mathrm{supp}\left(P_{XY}\right)\}. \end{array}$$

Then,

(137) $$\begin{array}{cc} \left(1-\alpha \right)\;J_{\alpha }\left(X;Y\right) & =\underset{(Q_{X},\;Q_{Y})\in Q}{\sup}\;\left(1-\alpha \right)\;D_{\alpha }\left(P_{XY}∥Q_{X}Q_{Y}\right) \end{array}$$

(138) =sup(QX,QY)∈QminRXY∈R(QX,QY)∈Q(1−α)D(RXY∥QXQY)+αD(RXY∥PXY)

(139) =minRXY∈R(QX,QY)∈Qsup(QX,QY)∈Q(1−α)D(RXY∥QXQY)+αD(RXY∥PXY)

(140) $$\begin{array}{c} =\min_{R_{XY}\in P\left(X\times Y\right)}\;\left[\left(1-\alpha \right)\;D\left(R_{XY}∥R_{X}R_{Y}\right)+\alpha \;D\left(R_{XY}∥P_{XY}\right)\right], \end{array}$$

where (137) follows from the definition of $J_{\alpha }\left(X;Y\right)$ because 1−α<0 and because the mapping $\left(Q_{X},Q_{Y}\right)\mapsto D_{\alpha }\left(P_{XY}∥Q_{X}Q_{Y}\right)$ is continuous; (138) follows from [22] (Theorem 30); (139) follows from a minimax theorem and is justified below; and (140) follows from Proposition 8, a continuity argument, and the observation that $D(R_{XY}∥P_{XY})$ is infinite if $R_{XY}\notin R$.

We now verify the conditions of Ky Fan’s minimax theorem [32] (Theorem 2), which will establish (139). (We use Ky Fan’s minimax theorem because it does not require that the set Q be compact, and having a noncompact set Q helps to guarantee that the function *f* defined next takes on finite values only. A brief proof of Ky Fan’s minimax theorem appears in [33].) Let the function f:R×Q→R be defined by the expression in square brackets in (139), i.e.,

(141) $$\begin{array}{c} f\left(R_{XY},Q_{X},Q_{Y}\right)≜\left(1-\alpha \right)\;D\left(R_{XY}∥Q_{X}Q_{Y}\right)+\alpha \;D\left(R_{XY}∥P_{XY}\right). \end{array}$$

We check that

(i) the sets Q and R are convex;
(ii) the set R is compact;
(iii) the function *f* is real-valued;
(iv) for every $(Q_{X},Q_{Y})\in Q$, the function *f* is continuous in $R_{XY}$;
(v) for every $(Q_{X},Q_{Y})\in Q$, the function *f* is convex in $R_{XY}$; and
(vi) for every $R_{XY}\in R$, the function *f* is concave in the pair $\left(Q_{X},Q_{Y}\right)$.

Indeed, Parts (i) and (ii) are easy to see; Part (iii) holds because both relative entropies on the RHS of (141) are finite by our definitions of Q and R; and to show Parts (iv)–(vi), we rewrite *f* as:

(142) f(RXY,QX,QY)=−H(RXY)−α∑x,yRXY(x,y)logP(x,y)=+(α−1)∑xRX(x)logQX(x)+(α−1)∑yRY(y)logQY(y).

From (142), we see that Part (iv) holds by our definitions of Q and R; Part (v) holds because the entropy is a concave function (so $-H(R_{XY})$ is convex), because linear functionals of $R_{XY}$ are convex, and because the sum of convex functions is convex; and Part (vi) holds because the logarithm is a concave function and because a nonnegative weighted sum of concave functions is concave. (In Ky Fan’s theorem, weaker conditions than Parts (i)–(vi) are required, but it is not difficult to see that Parts (i)–(vi) are sufficient.)

The last claim, namely, that the mapping $\alpha \mapsto \left(1-\alpha \right)\;J_{\alpha }\left(X;Y\right)$ is concave on (0,∞), is true because the expression in square brackets on the RHS of (131) is concave in α for every $R_{XY}$ and because the pointwise minimum preserves the concavity. □

**Lemma** **9.** *The mapping $\alpha \mapsto J_{\alpha }\left(X;Y\right)$ is nondecreasing on [0,∞].*

**Proof.**  This is true because for every $\alpha ,\alpha^{\prime }\in \left[0,\infty \right]$ with $\alpha \leq \alpha^{\prime }$,

(143) $$\begin{array}{c} \min_{Q_{X},\;Q_{Y}}D_{\alpha }\left(P_{XY}∥Q_{X}Q_{Y}\right)\leq \min_{Q_{X},\;Q_{Y}}D_{\alpha^{\prime }}\left(P_{XY}∥Q_{X}Q_{Y}\right), \end{array}$$

which holds because the Rényi divergence is nondecreasing in α (Proposition 4). □

**Lemma** **10.** *The mapping $\alpha \mapsto J_{\alpha }\left(X;Y\right)$ is continuous on [0,∞].*

**Proof.**  By Lemma 8, the mapping $\alpha \mapsto \left(1-\alpha \right)\;J_{\alpha }\left(X;Y\right)$ is concave on (0,∞), thus it is continuous on (0,∞), which implies that $\alpha \mapsto J_{\alpha }\left(X;Y\right)$ is continuous on (0,1)∪(1,∞).

We next prove the continuity at α=0. Let $Q_{X}^{*}$ and $Q_{Y}^{*}$ be PMFs that achieve the minimum in the definition of $J_{0}\left(X;Y\right)$. Then, for all α≥0,

(144) $$\begin{array}{cc} D_{0}\left(P_{XY}∥Q_{X}^{*}Q_{Y}^{*}\right) & =J_{0}\left(X;Y\right) \end{array}$$

(145) $$\begin{array}{c} \leq J_{\alpha }\left(X;Y\right) \end{array}$$

(146) $$\begin{array}{c} \leq D_{\alpha }\left(P_{XY}∥Q_{X}^{*}Q_{Y}^{*}\right), \end{array}$$

where (145) holds because $\alpha \mapsto J_{\alpha }\left(X;Y\right)$ is nondecreasing (Lemma 9), and (146) holds by the definition of $J_{\alpha }\left(X;Y\right)$. The Rényi divergence is continuous in α (Proposition 4), so (144)–(146) and the sandwich theorem imply that $J_{\alpha }\left(X;Y\right)$ is continuous at α=0.

We continue with the continuity at α=∞. Define

(147) $$\begin{array}{c} \tau ≜\min_{\left(x,y\right)\in \mathrm{supp}\left(P_{XY}\right)}P\left(x,y\right). \end{array}$$

Then, for all α>1,

(148) $$\begin{array}{cc} J_{\infty }\left(X;Y\right) & \geq J_{\alpha }\left(X;Y\right) \end{array}$$

(149) $$\begin{array}{c} =\min_{Q_{X},\;Q_{Y}}\frac{1}{\alpha -1}\log\sum_{x,y}P\left(x,y\right)\;\frac{P{\left(x,y\right)}^{\alpha -1}}{{\left[Q_{X}\left(x\right)\;Q_{Y}\left(y\right)\right]}^{\alpha -1}} \end{array}$$

(150) $$\begin{array}{c} \geq \min_{Q_{X},\;Q_{Y}}\frac{1}{\alpha -1}\log\max_{x,y}\frac{\tau \;P{\left(x,y\right)}^{\alpha -1}}{{\left[Q_{X}\left(x\right)\;Q_{Y}\left(y\right)\right]}^{\alpha -1}} \end{array}$$

(151) $$\begin{array}{c} =\frac{1}{\alpha -1}\log\tau +\min_{Q_{X},\;Q_{Y}}\log\max_{x,y}\frac{P(x,y)}{Q_{X}\left(x\right)\;Q_{Y}\left(y\right)} \end{array}$$

(152) $$\begin{array}{c} =\frac{1}{\alpha -1}\log\tau +J_{\infty }\left(X;Y\right), \end{array}$$

where (148) holds because $\alpha \mapsto J_{\alpha }\left(X;Y\right)$ is nondecreasing (Lemma 9), and (149) and (152) hold by the definitions of $J_{\alpha }\left(X;Y\right)$ and the Rényi divergence. The RHS of (152) tends to $J_{\infty }\left(X;Y\right)$ as α tends to infinity, so $J_{\alpha }\left(X;Y\right)$ is continuous at α=∞ by the sandwich theorem.

It remains to show the continuity at α=1. Let $\alpha \in \left(\frac{3}{4},1\right)\cup \left(1,\frac{5}{4}\right)$, and let $\delta ≜|1-\alpha |\in (0,\frac{1}{4})$. Then, for all PMFs $Q_{X}$ and $Q_{Y}$,

(153) $$\begin{array}{cc} 2^{-\delta D_{\alpha }\left(P_{XY}∥Q_{X}Q_{Y}\right)} & \leq 2^{-\delta D_{1-\delta }\left(P_{XY}∥Q_{X}Q_{Y}\right)} \end{array}$$

(154) $$\begin{array}{c} =\sum_{x,y}P\left(x,y\right){\left[\frac{Q_{X}\left(x\right)\;Q_{Y}\left(y\right)}{P(x,y)}\right]}^{\delta } \end{array}$$

(155) $$\begin{array}{c} =\sum_{x,y}P\left(x,y\right){\left[\frac{P_{X}\left(x\right)\;P_{Y}\left(y\right)}{P(x,y)}\right]}^{\delta }\;{\left[\frac{Q_{X}\left(x\right)\;Q_{Y}\left(y\right)}{P_{X}\left(x\right)\;P_{Y}\left(y\right)}\right]}^{\delta } \end{array}$$

(156) $$\begin{array}{c} \leq {\left\{\sum_{x,y}P\left(x,y\right){\left[\frac{P_{X}\left(x\right)\;P_{Y}\left(y\right)}{P(x,y)}\right]}^{2\delta }\right\}}^{\frac{1}{2}}\cdot {\left\{\sum_{x,y}P\left(x,y\right){\left[\frac{Q_{X}\left(x\right)\;Q_{Y}\left(y\right)}{P_{X}\left(x\right)\;P_{Y}\left(y\right)}\right]}^{2\delta }\right\}}^{\frac{1}{2}} \end{array}$$

(157) $$\begin{array}{c} \leq {\left\{\sum_{x,y}P\left(x,y\right){\left[\frac{P_{X}\left(x\right)\;P_{Y}\left(y\right)}{P(x,y)}\right]}^{2\delta }\right\}}^{\frac{1}{2}} \end{array}$$

(158) $$\begin{array}{c} =2^{-\delta D_{1-2\delta }\left(P_{XY}∥P_{X}P_{Y}\right)}, \end{array}$$

where (153) holds because 1−δ≤α and because the Rényi divergence is nondecreasing in α (Proposition 4); (156) follows from the Cauchy–Schwarz inequality; and (157) holds because

(159) $$\begin{array}{cc} {\left\{\sum_{x,y}P\left(x,y\right){\left[\frac{Q_{X}\left(x\right)\;Q_{Y}\left(y\right)}{P_{X}\left(x\right)\;P_{Y}\left(y\right)}\right]}^{2\delta }\right\}}^{\frac{1}{2}} & \leq {\left\{\sum_{x}P_{X}\left(x\right){\left[\frac{Q_{X}\left(x\right)}{P_{X}\left(x\right)}\right]}^{4\delta }\right\}}^{\frac{1}{4}}\cdot {\left\{\sum_{y}P_{Y}\left(y\right){\left[\frac{Q_{Y}\left(y\right)}{P_{Y}\left(y\right)}\right]}^{4\delta }\right\}}^{\frac{1}{4}} \end{array}$$

(160) $$\begin{array}{c} =2^{-\delta D_{1-4\delta }\left(P_{X}∥Q_{X}\right)}\cdot 2^{-\delta D_{1-4\delta }\left(P_{Y}∥Q_{Y}\right)} \end{array}$$

(161) $$\begin{array}{c} \leq 1, \end{array}$$

where (159) follows from the Cauchy–Schwarz inequality, and (161) holds because 1−4δ>0 and because the Rényi divergence is nonnegative for positive orders (Proposition 4). Thus, for all $\alpha \in (\frac{3}{4},\frac{5}{4})$,

(162) $$\begin{array}{cc} D_{1-2|1-\alpha |}\left(P_{XY}∥P_{X}P_{Y}\right) & \leq \min_{Q_{X},\;Q_{Y}}D_{\alpha }\left(P_{XY}∥Q_{X}Q_{Y}\right) \end{array}$$

(163) $$\begin{array}{c} =J_{\alpha }\left(X;Y\right) \end{array}$$

(164) $$\begin{array}{c} \leq D_{\alpha }\left(P_{XY}∥P_{X}P_{Y}\right), \end{array}$$

where (162) follows from (158) if α≠1 and from Proposition 8 if α=1; and (164) holds by the definition of $J_{\alpha }\left(X;Y\right)$. The Rényi divergence is continuous in α (Proposition 4), thus (162)–(164) and the sandwich theorem imply that $J_{\alpha }\left(X;Y\right)$ is continuous at α=1. □

**Lemma** **11.** *If X=Y with probability one, then*

(165) $$\begin{array}{c} J_{\alpha }\left(X;Y\right)=\left\{\begin{array}{cc} \frac{\alpha }{1-\alpha }\;H_{\infty }\left(X\right) & if\;\alpha \in [0,\frac{1}{2}],\\ H_{\frac{\alpha }{2\alpha -1}}\left(X\right) & if\;\alpha >\frac{1}{2},\\ H_{\frac{1}{2}}\left(X\right) & if\;\alpha =\infty . \end{array}\right. \end{array}$$

**Proof.**  We show below that (165) holds for α∈(0,1)∪(1,∞). Thus, (165) holds also for α∈{0,1,∞} because both its sides are continuous in α: its LHS by Lemma 10, and its RHS by the continuity of the Rényi entropy (Proposition 3).

Fix α∈(0,1)∪(1,∞). Then,

(166) $$\begin{array}{cc} J_{\alpha }\left(X;Y\right) & =\min_{Q_{X}}\min_{Q_{Y}}D_{\alpha }\left(P_{XY}∥Q_{X}Q_{Y}\right) \end{array}$$

(167) $$\begin{array}{c} =\min_{Q_{X}}\frac{\alpha }{\alpha -1}\log\sum_{y}{\left[\sum_{x}P{\left(x,y\right)}^{\alpha }\;Q_{X}{\left(x\right)}^{1-\alpha }\right]}^{\frac{1}{\alpha }} \end{array}$$

(168) $$\begin{array}{c} =\min_{Q_{X}}\frac{\alpha }{\alpha -1}\log\sum_{x}P_{X}\left(x\right)\;Q_{X}{\left(x\right)}^{\frac{1-\alpha }{\alpha }}, \end{array}$$

where (166) follows from Proposition 9, and (168) holds because

(169) $$\begin{array}{c} P_{XY}\left(x,y\right)=\left\{\begin{array}{cc} P_{X}\left(x\right) & \mathrm{if}\;x=y,\\ 0 & \mathrm{otherwise}. \end{array}\right. \end{array}$$

First consider the case $\alpha >\frac{1}{2}$. Define $\gamma ≜\sum_{x}P_{X}{\left(x\right)}^{\frac{\alpha }{2\alpha -1}}$. Then, for all $Q_{X}\in P\left(X\right)$,

(170) $$\begin{array}{cc} \frac{\alpha }{\alpha -1}\log\sum_{x}P_{X}\left(x\right)\;Q_{X}{\left(x\right)}^{\frac{1-\alpha }{\alpha }} & =\frac{\alpha }{\alpha -1}\log\sum_{x}{\left[\gamma \;\gamma^{-1}\;P_{X}{\left(x\right)}^{\frac{\alpha }{2\alpha -1}}\right]}^{\frac{2\alpha -1}{\alpha }}Q_{X}{\left(x\right)}^{\frac{1-\alpha }{\alpha }} \end{array}$$

(171) $$\begin{array}{c} =\frac{2\alpha -1}{\alpha -1}\log\gamma +D_{\frac{2\alpha -1}{\alpha }}\left(\gamma^{-1}\;P_{X}{}^{\frac{\alpha }{2\alpha -1}}∥Q_{X}\right) \end{array}$$

(172) $$\begin{array}{c} =H_{\frac{\alpha }{2\alpha -1}}\left(X\right)+D_{\frac{2\alpha -1}{\alpha }}\left(\gamma^{-1}\;P_{X}{}^{\frac{\alpha }{2\alpha -1}}∥Q_{X}\right), \end{array}$$

where (171) holds because $x\mapsto \gamma^{-1}\;P_{X}{\left(x\right)}^{\frac{\alpha }{2\alpha -1}}$ is a PMF. Because $\frac{2\alpha -1}{\alpha }>0$, Proposition 4 implies that $D_{(2\alpha -1)/\alpha }\left(P∥Q\right)\geq 0$ with equality if Q=P. This together with (168) and (172) establishes (165).

Now consider the case $\alpha \in (0,\frac{1}{2}]$. For all $Q_{X}\in P\left(X\right)$,

(173) $$\begin{array}{cc} \sum_{x}P_{X}\left(x\right)\;Q_{X}{\left(x\right)}^{\frac{1-\alpha }{\alpha }} & \leq \sum_{x}P_{X}\left(x\right)\;Q_{X}\left(x\right) \end{array}$$

(174) $$\begin{array}{c} \leq \sum_{x}\left[\max_{x^{\prime }}P_{X}\left(x^{\prime }\right)\right]Q_{X}\left(x\right) \end{array}$$

(175) $$\begin{array}{c} =\max_{x}P_{X}\left(x\right), \end{array}$$

where (173) holds because $Q_{X}\left(x\right)\in \left[0,1\right]$ for all x∈X and because $\frac{1-\alpha }{\alpha }\geq 1$. The inequalities (173) and (174) both hold with equality when $Q_{X}\left(x\right)=𝟙\left\{x=x^{*}\right\}$, where $x^{*}\in X$ is such that $P_{X}\left(x^{*}\right)=\max_{x}P_{X}\left(x\right)$. Thus,

(176) $$\begin{array}{c} \max_{Q_{X}}\sum_{x}P_{X}\left(x\right)\;Q_{X}{\left(x\right)}^{\frac{1-\alpha }{\alpha }}=\max_{x}P_{X}\left(x\right). \end{array}$$

Now (165) follows:

(177) $$\begin{array}{cc} J_{\alpha }\left(X;Y\right) & =\min_{Q_{X}}\frac{\alpha }{\alpha -1}\log\sum_{x}P_{X}\left(x\right)\;Q_{X}{\left(x\right)}^{\frac{1-\alpha }{\alpha }} \end{array}$$

(178) $$\begin{array}{c} =\frac{\alpha }{\alpha -1}\log\max_{Q_{X}}\sum_{x}P_{X}\left(x\right)\;Q_{X}{\left(x\right)}^{\frac{1-\alpha }{\alpha }} \end{array}$$

(179) $$\begin{array}{c} =\frac{\alpha }{\alpha -1}\log\max_{x}P_{X}\left(x\right) \end{array}$$

(180) $$\begin{array}{c} =\frac{\alpha }{1-\alpha }\;H_{\infty }\left(X\right), \end{array}$$

where (177) follows from (168); (178) holds because $\frac{\alpha }{\alpha -1}<0$; (179) follows from (176); and (180) follows from the definition of $H_{\infty }\left(X\right)$. □

**Lemma** **12.** *If the pairs $\left(X_{1},Y_{1}\right)$ and $\left(X_{2},Y_{2}\right)$ are independent, then $J_{\alpha }\left(X_{1},X_{2};Y_{1},Y_{2}\right)=J_{\alpha }\left(X_{1};Y_{1}\right)+J_{\alpha }\left(X_{2};Y_{2}\right)$ for all α∈[0,∞] (additivity).*

**Proof.**  Let the pairs $\left(X_{1},Y_{1}\right)$ and $\left(X_{2},Y_{2}\right)$ be independent. For α∈(0,1)∪(1,∞), we establish the lemma by showing the following two inequalities:

(181) $$\begin{array}{cc} J_{\alpha }\left(X_{1},X_{2};Y_{1},Y_{2}\right) & \leq J_{\alpha }\left(X_{1};Y_{1}\right)+J_{\alpha }\left(X_{2};Y_{2}\right), \end{array}$$

(182) $$\begin{array}{cc} J_{\alpha }\left(X_{1},X_{2};Y_{1},Y_{2}\right) & \geq J_{\alpha }\left(X_{1};Y_{1}\right)+J_{\alpha }\left(X_{2};Y_{2}\right). \end{array}$$

Because $J_{\alpha }\left(X;Y\right)$ is continuous in α (Lemma 10), this will also establish the lemma for α∈{0,1,∞}.

To show (181), let $Q_{X_{1}}^{*}$ and $Q_{Y_{1}}^{*}$ be PMFs that achieve the minimum in the definition of $J_{\alpha }\left(X_{1};Y_{1}\right)$, and let $Q_{X_{2}}^{*}$ and $Q_{Y_{2}}^{*}$ be PMFs that achieve the minimum in the definition of $J_{\alpha }\left(X_{2};Y_{2}\right)$, so

(183) $$\begin{array}{cc} J_{\alpha }\left(X_{1};Y_{1}\right) & =D_{\alpha }\left(P_{X_{1}Y_{1}}∥Q_{X_{1}}^{*}Q_{Y_{1}}^{*}\right), \end{array}$$

(184) $$\begin{array}{cc} J_{\alpha }\left(X_{2};Y_{2}\right) & =D_{\alpha }\left(P_{X_{2}Y_{2}}∥Q_{X_{2}}^{*}Q_{Y_{2}}^{*}\right). \end{array}$$

Then, (181) holds because

(185) $$\begin{array}{cc} J_{\alpha }\left(X_{1},X_{2};Y_{1},Y_{2}\right) & \leq D_{\alpha }\left(P_{X_{1}X_{2}Y_{1}Y_{2}}∥Q_{X_{1}}^{*}Q_{X_{2}}^{*}Q_{Y_{1}}^{*}Q_{Y_{2}}^{*}\right) \end{array}$$

(186) $$\begin{array}{c} =D_{\alpha }\left(P_{X_{1}Y_{1}}∥Q_{X_{1}}^{*}Q_{Y_{1}}^{*}\right)+D_{\alpha }\left(P_{X_{2}Y_{2}}∥Q_{X_{2}}^{*}Q_{Y_{2}}^{*}\right) \end{array}$$

(187) $$\begin{array}{c} =J_{\alpha }\left(X_{1};Y_{1}\right)+J_{\alpha }\left(X_{2};Y_{2}\right), \end{array}$$

where (185) holds by the definition of $J_{\alpha }\left(X_{1},X_{2};Y_{1},Y_{2}\right)$ as a minimum; (186) follows from a simple computation using the independence hypothesis $P_{X_{1}X_{2}Y_{1}Y_{2}}=P_{X_{1}Y_{1}}P_{X_{2}Y_{2}}$; and (187) follows from (183) and (184).

To establish (182), we consider the cases α>1 and α<1 separately, starting with α>1. Let ${\hat{Q}}_{X_{1}X_{2}}$ and ${\hat{Q}}_{Y_{1}Y_{2}}$ be PMFs that achieve the minimum in the definition of $J_{\alpha }\left(X_{1},X_{2};Y_{1},Y_{2}\right)$, so

(188) $$\begin{array}{c} J_{\alpha }\left(X_{1},X_{2};Y_{1},Y_{2}\right)=D_{\alpha }\left(P_{X_{1}X_{2}Y_{1}Y_{2}}∥{\hat{Q}}_{X_{1}X_{2}}{\hat{Q}}_{Y_{1}Y_{2}}\right). \end{array}$$

Define the function $f:X_{1}\times Y_{1}\to R\cup \left\{\infty \right\}$ as

(189) $$\begin{array}{c} f\left(x_{1},y_{1}\right)≜\sum_{x_{2},y_{2}}P_{X_{2}Y_{2}}{\left(x_{2},y_{2}\right)}^{\alpha }{\left[{\hat{Q}}_{X_{2}|X_{1}}\left(x_{2}|x_{1}\right)\;{\hat{Q}}_{Y_{2}|Y_{1}}\left(y_{2}|y_{1}\right)\right]}^{1-\alpha }, \end{array}$$

and let $\left(x_{1}^{\prime },y_{1}^{\prime }\right)\in X_{1}\times Y_{1}$ be such that

(190) $$\begin{array}{c} f\left(x_{1}^{\prime },y_{1}^{\prime }\right)=\min_{x_{1},y_{1}}f\left(x_{1},y_{1}\right). \end{array}$$

Define the PMFs $Q_{X_{2}}^{\prime }$ and $Q_{Y_{2}}^{\prime }$ as

(191) $$\begin{array}{cc} Q_{X_{2}}^{\prime }\left(x_{2}\right) & ≜{\hat{Q}}_{X_{2}|X_{1}}\left(x_{2}|x_{1}^{\prime }\right), \end{array}$$

(192) $$\begin{array}{cc} Q_{Y_{2}}^{\prime }\left(y_{2}\right) & ≜{\hat{Q}}_{Y_{2}|Y_{1}}\left(y_{2}|y_{1}^{\prime }\right). \end{array}$$

Then,

(193) $$\begin{array}{cc} 2^{\left(\alpha -1\right)J_{\alpha }\left(X_{1},X_{2};Y_{1},Y_{2}\right)} & =2^{\left(\alpha -1\right)D_{\alpha }\left(P_{X_{1}X_{2}Y_{1}Y_{2}}∥{\hat{Q}}_{X_{1}X_{2}}{\hat{Q}}_{Y_{1}Y_{2}}\right)} \end{array}$$

(194) $$\begin{array}{c} =\sum_{x_{1},x_{2},y_{1},y_{2}}{\left[P_{X_{1}Y_{1}}\left(x_{1},y_{1}\right)\;P_{X_{2}Y_{2}}\left(x_{2},y_{2}\right)\right]}^{\alpha }{\left[{\hat{Q}}_{X_{1}X_{2}}\left(x_{1},x_{2}\right)\;{\hat{Q}}_{Y_{1}Y_{2}}\left(y_{1},y_{2}\right)\right]}^{1-\alpha } \end{array}$$

(195) $$\begin{array}{c} =\sum_{x_{1},y_{1}}P_{X_{1}Y_{1}}{\left(x_{1},y_{1}\right)}^{\alpha }{\left[{\hat{Q}}_{X_{1}}\left(x_{1}\right)\;{\hat{Q}}_{Y_{1}}\left(y_{1}\right)\right]}^{1-\alpha }\;f\left(x_{1},y_{1}\right) \end{array}$$

(196) $$\begin{array}{c} \geq \sum_{x_{1},y_{1}}P_{X_{1}Y_{1}}{\left(x_{1},y_{1}\right)}^{\alpha }{\left[{\hat{Q}}_{X_{1}}\left(x_{1}\right)\;{\hat{Q}}_{Y_{1}}\left(y_{1}\right)\right]}^{1-\alpha }\;f\left(x_{1}^{\prime },y_{1}^{\prime }\right) \end{array}$$

(197) $$\begin{array}{c} =2^{\left(\alpha -1\right)D_{\alpha }\left(P_{X_{1}Y_{1}}∥{\hat{Q}}_{X_{1}}{\hat{Q}}_{Y_{1}}\right)+\left(\alpha -1\right)D_{\alpha }\left(P_{X_{2}Y_{2}}∥Q_{X_{2}}^{\prime }Q_{Y_{2}}^{\prime }\right)}, \end{array}$$

where (193) follows from (188); (194) holds by the independence hypothesis $P_{X_{1}X_{2}Y_{1}Y_{2}}=P_{X_{1}Y_{1}}P_{X_{2}Y_{2}}$; (195) follows from (189); (196) follows from (190); and (197) follows from (191) and (192). Taking the logarithm and multiplying by $\frac{1}{\alpha -1}>0$ establishes (182):

(198) $$\begin{array}{cc} J_{\alpha }\left(X_{1},X_{2};Y_{1},Y_{2}\right) & \geq D_{\alpha }\left(P_{X_{1}Y_{1}}∥{\hat{Q}}_{X_{1}}{\hat{Q}}_{Y_{1}}\right)+D_{\alpha }\left(P_{X_{2}Y_{2}}∥Q_{X_{2}}^{\prime }Q_{Y_{2}}^{\prime }\right) \end{array}$$

(199) $$\begin{array}{c} \geq J_{\alpha }\left(X_{1};Y_{1}\right)+J_{\alpha }\left(X_{2};Y_{2}\right), \end{array}$$

where (199) holds by the definition of $J_{\alpha }\left(X_{1};Y_{1}\right)$ and $J_{\alpha }\left(X_{2};Y_{2}\right)$.

The proof of (182) for α∈(0,1) is essentially the same as for α>1: Replace the minimum in (190) by a maximum. Inequality (196) is then reversed, but (198) continues to hold because $\frac{1}{\alpha -1}<0$. Inequality (199) also continues to hold, and (198) and (199) together imply (182).

**Lemma** **13.** *For all α∈[0,∞], $J_{\alpha }\left(X;Y\right)\leq \log\;\left|X\right|$ with equality if and only if $\left(\alpha \in [\frac{1}{2},\infty \right]$, X is distributed uniformly over X, and H(X|Y)=0).*

**Proof.**  Throughout the proof, define $X^{\prime }≜X$. We first show that $J_{\alpha }\left(X;Y\right)\leq \log\;\left|X\right|$ for all α∈[0,∞]:

(200) $$\begin{array}{cc} J_{\alpha }\left(X;Y\right) & \leq J_{\alpha }\left(X;X^{\prime }\right) \end{array}$$

(201) $$\begin{array}{c} \leq J_{\infty }\left(X;X^{\prime }\right) \end{array}$$

(202) $$\begin{array}{c} =H_{\frac{1}{2}}\left(X\right) \end{array}$$

(203) $$\begin{array}{c} \leq \log\;|X|, \end{array}$$

where (200) follows from the data-processing inequality (Lemma 4) because $X⊸-\;-X^{\prime }⊸-\;-Y$ form a Markov chain; (201) holds because $J_{\alpha }\left(X;X^{\prime }\right)$ is nondecreasing in α (Lemma 9); (202) follows from Lemma 11; and (203) follows from Proposition 3.

We now show that (200)–(203) can hold with equality only if the following conditions all hold:

(1) $\alpha \in [\frac{1}{2},\infty ]$;
(ii) *X* is distributed uniformly over X; and
(iii) H(X|Y)=0, i.e., for every $y\in \mathrm{supp}(P_{Y})$, there exists an x∈X for which P(x|y)=1.

Indeed, if $\alpha <\frac{1}{2}$, then Lemma 11 implies that

(204) $$\begin{array}{c} J_{\alpha }\left(X;X^{\prime }\right)=\frac{\alpha }{1-\alpha }\;H_{\infty }\left(X\right). \end{array}$$

Because $\frac{\alpha }{1-\alpha }<1$ for such α’s and because $H_{\infty }\left(X\right)\leq \log\;\left|X\right|$ (Proposition 3), the RHS of (204) is strictly smaller than log|X|. This, together with (200), shows that Part (i) is a necessary condition. The necessity of Part (ii) follows from (203): if *X* is not distributed uniformly over X, then (203) holds with strict inequality (Proposition 3). As to the necessity of Part (iii),

(205) $$\begin{array}{cc} J_{\alpha }\left(X;Y\right) & \leq J_{\infty }\left(X;Y\right) \end{array}$$

(206) $$\begin{array}{c} =\min_{Q_{X}}\min_{Q_{Y}}D_{\infty }\left(P_{XY}∥Q_{X}Q_{Y}\right) \end{array}$$

(207) $$\begin{array}{c} =\min_{Q_{X}}\log\sum_{y}\max_{x}\frac{P(x,y)}{Q_{X}\left(x\right)} \end{array}$$

(208) $$\begin{array}{c} \leq \log\sum_{y}\max_{x}\frac{P(y)\;P(x|y)}{1/|X|} \end{array}$$

(209) $$\begin{array}{c} =\log\;\left|X\right|+\log\sum_{y}P\left(y\right)\max_{x}P\left(x|y\right) \end{array}$$

(210) $$\begin{array}{c} \leq \log\;|X|, \end{array}$$

where (205) holds because $J_{\alpha }\left(X;Y\right)$ is nondecreasing in α (Lemma 9); (207) follows from Proposition 9; and (208) follows from choosing $Q_{X}$ to be the uniform distribution. The inequality (210) is strict when Part (iii) does not hold, so Part (iii) is a necessary condition.

It remains to show that when Parts (i)–(iii) all hold, $J_{\alpha }\left(X;Y\right)=\log\;\left|X\right|$. By (203), $J_{\alpha }\left(X;Y\right)\leq \log\;\left|X\right|$ always holds, so it suffices to show that Parts (i)–(iii) together imply $J_{\alpha }\left(X;Y\right)\geq \log\;\left|X\right|$. Indeed,

(211) $$\begin{array}{cc} J_{\alpha }\left(X;Y\right) & \geq J_{\frac{1}{2}}\left(X;Y\right) \end{array}$$

(212) $$\begin{array}{c} \geq J_{\frac{1}{2}}\left(X;X^{\prime }\right) \end{array}$$

(213) $$\begin{array}{c} =H_{\infty }\left(X\right) \end{array}$$

(214) $$\begin{array}{c} =\log\;|X|, \end{array}$$

where (211) holds because Part (i) implies that $\alpha \geq \frac{1}{2}$ and because $J_{\alpha }\left(X;Y\right)$ is nondecreasing in α (Lemma 9); (212) follows from the data-processing inequality (Lemma 4) because Part (iii) implies that $X⊸-\;-Y⊸-\;-X^{\prime }$ form a Markov chain; (213) follows from Lemma 11; and (214) follows from Part (ii). □

**Lemma** **14.** *For every α∈[1,∞], $J_{\alpha }\left(X;Y\right)$ is concave in $P_{X}$ for fixed $P_{Y|X}$.*

**Proof.**  We prove the claim for α∈(1,∞); for α∈{1,∞} the claim will then hold because $J_{\alpha }\left(X;Y\right)$ is continuous in α (Lemma 10).

Fix α∈(1,∞). Let $\lambda ,\lambda^{\prime }\in \left[0,1\right]$ with $\lambda +\lambda^{\prime }=1$, let $P_{X}$ and $P_{X}^{\prime }$ be PMFs, let $P_{Y|X}$ be a conditional PMF, and define f:X×P(Y)→R∪{∞} as

(215) $$\begin{array}{c} f\left(x,Q_{Y}\right)≜{\left[\sum_{y}P_{Y|X}{\left(y|x\right)}^{\alpha }\;Q_{Y}{\left(y\right)}^{1-\alpha }\right]}^{\frac{1}{\alpha }}. \end{array}$$

Denoting $J_{\alpha }\left(X;Y\right)$ by $J_{\alpha }\left(P_{X}P_{Y|X}\right)$,

$$\begin{array}{c} J_{\alpha }\left(\left(\lambda \;P_{X}+\lambda^{\prime }\;P_{X}^{\prime }\right)P_{Y|X}\right) \end{array}$$

(216) $$\begin{array}{c} \;=\min_{Q_{Y}}\min_{Q_{X}}D_{\alpha }\left(\left(\lambda \;P_{X}+\lambda^{\prime }\;P_{X}^{\prime }\right)P_{Y|X}∥Q_{X}Q_{Y}\right) \end{array}$$

(217) $$\begin{array}{c} \;=\min_{Q_{Y}}\frac{\alpha }{\alpha -1}\log\sum_{x}{\left[\sum_{y}\;{\left[\lambda \;P_{X}\left(x\right)+\lambda^{\prime }\;P_{X}^{\prime }\left(x\right)\right]}^{\alpha }\;P_{Y|X}{\left(y|x\right)}^{\alpha }\;Q_{Y}{\left(y\right)}^{1-\alpha }\right]}^{\frac{1}{\alpha }} \end{array}$$

(218) $$\begin{array}{c} \;=\min_{Q_{Y}}\frac{\alpha }{\alpha -1}\log\sum_{x}\;\left[\lambda \;P_{X}\left(x\right)+\lambda^{\prime }\;P_{X}^{\prime }\left(x\right)\right]{\left[\sum_{y}P_{Y|X}{\left(y|x\right)}^{\alpha }\;Q_{Y}{\left(y\right)}^{1-\alpha }\right]}^{\frac{1}{\alpha }} \end{array}$$

(219) $$\begin{array}{c} \;=\min_{Q_{Y}}\frac{\alpha }{\alpha -1}\log\left[\lambda \sum_{x}P_{X}\left(x\right)\;f\left(x,Q_{Y}\right)+\lambda^{\prime }\sum_{x}P_{X}^{\prime }\left(x\right)\;f\left(x,Q_{Y}\right)\right] \end{array}$$

(220) $$\begin{array}{c} \;\geq \min_{Q_{Y}}\frac{\alpha }{\alpha -1}\left[\lambda \log\sum_{x}P_{X}\left(x\right)\;f\left(x,Q_{Y}\right)+\lambda^{\prime }\log\sum_{x}P_{X}^{\prime }\left(x\right)\;f\left(x,Q_{Y}\right)\right] \end{array}$$

(221) $$\begin{array}{c} \;\geq \lambda \min_{Q_{Y}}\frac{\alpha }{\alpha -1}\log\sum_{x}P_{X}\left(x\right)\;f\left(x,Q_{Y}\right)+\lambda^{\prime }\min_{Q_{Y}}\frac{\alpha }{\alpha -1}\log\sum_{x}P_{X}^{\prime }\left(x\right)\;f\left(x,Q_{Y}\right) \end{array}$$

(222) $$\begin{array}{c} \;=\lambda \;J_{\alpha }\left(P_{X}P_{Y|X}\right)+\lambda^{\prime }\;J_{\alpha }\left(P_{X}^{\prime }P_{Y|X}\right), \end{array}$$

where (217) follows from Proposition 9 with the roles of $Q_{X}$ and $Q_{Y}$ swapped; (220) holds because log(·) is concave; (221) holds because optimizing $Q_{Y}$ separately cannot be worse than optimizing a common $Q_{Y}$; and (222) can be established using steps similar to (216)–(218). □

**Lemma** **15.** *For every $\alpha \in [\frac{1}{2},\infty ]$, the mapping $\left(Q_{X},Q_{Y}\right)\mapsto D_{\alpha }\left(P_{XY}∥Q_{X}Q_{Y}\right)$ is convex, i.e., for all $\lambda ,\lambda^{\prime }\in \left[0,1\right]$ with $\lambda +\lambda^{\prime }=1$, all $Q_{X},Q_{X}^{\prime }\in P\left(X\right)$, and all $Q_{Y},Q_{Y}^{\prime }\in P\left(Y\right)$,*

(223) $$\begin{array}{c} D_{\alpha }\left(P_{XY}∥\left(\lambda Q_{X}+\lambda^{\prime }Q_{X}^{\prime }\right)\left(\lambda Q_{Y}+\lambda^{\prime }Q_{Y}^{\prime }\right)\right)\leq \lambda \;D_{\alpha }\left(P_{XY}∥Q_{X}Q_{Y}\right)+\lambda^{\prime }\;D_{\alpha }\left(P_{XY}∥Q_{X}^{\prime }Q_{Y}^{\prime }\right). \end{array}$$

*For $\alpha \in [0,\frac{1}{2})$, the mapping need not be convex.*

**Proof.**  We establish (223) for $\alpha \in [\frac{1}{2},1)$ and for α∈(1,∞), which also establishes (223) for α∈{1,∞} because the Rényi divergence is continuous in α (Proposition 4). Afterwards, we provide an example where (223) is violated for all $\alpha \in [0,\frac{1}{2})$.

We begin with the case where $\alpha \in [\frac{1}{2},1)$:

(224) $$\begin{array}{c} 2^{\left(\alpha -1\right)\lambda D_{\alpha }\left(P_{XY}∥Q_{X}Q_{Y}\right)+\left(\alpha -1\right)\lambda^{\prime }D_{\alpha }\left(P_{XY}∥Q_{X}^{\prime }Q_{Y}^{\prime }\right)}\\ \;={\left[\sum_{x,y}P{\left(x,y\right)}^{\alpha }\;{\left[Q_{X}\left(x\right)\;Q_{Y}\left(y\right)\right]}^{1-\alpha }\right]}^{\lambda }\cdot {\left[\sum_{x,y}P{\left(x,y\right)}^{\alpha }\;{\left[Q_{X}^{\prime }\left(x\right)\;Q_{Y}^{\prime }\left(y\right)\right]}^{1-\alpha }\right]}^{\lambda^{\prime }} \end{array}$$

(225) $$\begin{array}{c} \;\leq \lambda \sum_{x,y}P{\left(x,y\right)}^{\alpha }\;{\left[Q_{X}\left(x\right)\;Q_{Y}\left(y\right)\right]}^{1-\alpha }+\lambda^{\prime }\sum_{x,y}P{\left(x,y\right)}^{\alpha }\;{\left[Q_{X}^{\prime }\left(x\right)\;Q_{Y}^{\prime }\left(y\right)\right]}^{1-\alpha } \end{array}$$

(226) $$\begin{array}{c} \;=\sum_{x,y}P{\left(x,y\right)}^{\alpha }\left[\sqrt{\lambda }\;Q_{X}{\left(x\right)}^{1-\alpha }\;\sqrt{\lambda }\;Q_{Y}{\left(y\right)}^{1-\alpha }+\sqrt{\lambda^{\prime }}\;Q_{X}^{\prime }{\left(x\right)}^{1-\alpha }\;\sqrt{\lambda^{\prime }}\;Q_{Y}^{\prime }{\left(y\right)}^{1-\alpha }\right] \end{array}$$

(227) $$\begin{array}{c} \;\leq \sum_{x,y}P{\left(x,y\right)}^{\alpha }\sqrt{\lambda \;Q_{X}{\left(x\right)}^{2(1-\alpha )}+\lambda^{\prime }\;Q_{X}^{\prime }{\left(x\right)}^{2(1-\alpha )}}\;\sqrt{\lambda \;Q_{Y}{\left(y\right)}^{2(1-\alpha )}+\lambda^{\prime }\;Q_{Y}^{\prime }{\left(y\right)}^{2(1-\alpha )}} \end{array}$$

(228) $$\begin{array}{c} \;\leq \sum_{x,y}P{\left(x,y\right)}^{\alpha }{\left[\lambda \;Q_{X}\left(x\right)+\lambda^{\prime }\;Q_{X}^{\prime }\left(x\right)\right]}^{1-\alpha }\;\sqrt{\lambda \;Q_{Y}{\left(y\right)}^{2(1-\alpha )}+\lambda^{\prime }\;Q_{Y}^{\prime }{\left(y\right)}^{2(1-\alpha )}} \end{array}$$

(229) $$\begin{array}{c} \;\leq \sum_{x,y}P{\left(x,y\right)}^{\alpha }{\left[\lambda \;Q_{X}\left(x\right)+\lambda^{\prime }\;Q_{X}^{\prime }\left(x\right)\right]}^{1-\alpha }\;{\left[\lambda \;Q_{Y}\left(y\right)+\lambda^{\prime }\;Q_{Y}^{\prime }\left(y\right)\right]}^{1-\alpha } \end{array}$$

(230) $$\begin{array}{c} \;=2^{\left(\alpha -1\right)D_{\alpha }\left(P_{XY}∥\left(\lambda Q_{X}+\lambda^{\prime }Q_{X}^{\prime }\right)\left(\lambda Q_{Y}+\lambda^{\prime }Q_{Y}^{\prime }\right)\right)}, \end{array}$$

where (225) follows from the arithmetic mean-geometric mean inequality; (227) follows from the Cauchy–Schwarz inequality; and (228) and (229) hold because the mapping $z\mapsto z^{2(1-\alpha )}$ is concave on $R_{\geq 0}$ for $\alpha \in [\frac{1}{2},1)$. Taking the logarithm and multiplying by $\frac{1}{\alpha -1}<0$ establishes (223).

Now, consider α∈(1,∞). Then,

(231) $$\begin{array}{c} 2^{\left(\alpha -1\right)D_{\alpha }\left(P_{XY}∥\left(\lambda Q_{X}+\lambda^{\prime }Q_{X}^{\prime }\right)\left(\lambda Q_{Y}+\lambda^{\prime }Q_{Y}^{\prime }\right)\right)}\\ \;=\sum_{x,y}P{\left(x,y\right)}^{\alpha }{\left[\lambda \;Q_{X}\left(x\right)+\lambda^{\prime }\;Q_{X}^{\prime }\left(x\right)\right]}^{1-\alpha }{\left[\lambda \;Q_{Y}\left(y\right)+\lambda^{\prime }\;Q_{Y}^{\prime }\left(y\right)\right]}^{1-\alpha } \end{array}$$

(232) $$\begin{array}{c} \;\leq \sum_{x,y}P{\left(x,y\right)}^{\alpha }{\left[Q_{X}{\left(x\right)}^{\lambda }\;Q_{X}^{\prime }{\left(x\right)}^{\lambda^{\prime }}\right]}^{1-\alpha }{\left[Q_{Y}{\left(y\right)}^{\lambda }\;Q_{Y}^{\prime }{\left(y\right)}^{\lambda^{\prime }}\right]}^{1-\alpha } \end{array}$$

(233) $$\begin{array}{c} \;=\sum_{x,y}P{\left(x,y\right)}^{\alpha }{\left[Q_{X}\left(x\right)\;Q_{Y}\left(y\right)\right]}^{(1-\alpha )\lambda }{\left[Q_{X}^{\prime }\left(x\right)\;Q_{Y}^{\prime }\left(y\right)\right]}^{\left(1-\alpha \right)\lambda^{\prime }} \end{array}$$

(234) $$\begin{array}{c} \;\leq {\left[\sum_{x,y}P{\left(x,y\right)}^{\alpha }{\left[Q_{X}\left(x\right)\;Q_{Y}\left(y\right)\right]}^{1-\alpha }\right]}^{\lambda }\cdot {\left[\sum_{x,y}P{\left(x,y\right)}^{\alpha }{\left[Q_{X}^{\prime }\left(x\right)\;Q_{Y}^{\prime }\left(y\right)\right]}^{1-\alpha }\right]}^{\lambda^{\prime }} \end{array}$$

(235) $$\begin{array}{c} \;=2^{\left(\alpha -1\right)\lambda D_{\alpha }\left(P_{XY}∥Q_{X}Q_{Y}\right)+\left(\alpha -1\right)\lambda^{\prime }D_{\alpha }\left(P_{XY}∥Q_{X}^{\prime }Q_{Y}^{\prime }\right)}, \end{array}$$

where (232) follows from the arithmetic mean-geometric mean inequality and the fact that the mapping $z\mapsto z^{1-\alpha }$ is decreasing on $R_{>0}$ for α>1, and (233) follows from Hölder’s inequality. Taking the logarithm and multiplying by $\frac{1}{\alpha -1}>0$ establishes (223).

Finally, we show that the mapping $\left(Q_{X},Q_{Y}\right)\mapsto D_{\alpha }\left(P_{XY}∥Q_{X}Q_{Y}\right)$ does not need to be convex for $\alpha \in [0,\frac{1}{2})$. Let *X* be uniformly distributed over {0,1}, and let Y=X. Then, for all $\alpha \in [0,\frac{1}{2})$,

(236) $$\begin{array}{c} D_{\alpha }\left(P_{XY}∥\left(0.5,0.5\right)\left(0.5,0.5\right)\right)>0.5\;D_{\alpha }\left(P_{XY}∥\left(1,0\right)\left(1,0\right)\right)+0.5\;D_{\alpha }\left(P_{XY}∥\left(0,1\right)\left(0,1\right)\right), \end{array}$$

because the LHS of (236) is equal to log2, and the RHS of (236) is equal to $\frac{\alpha }{1-\alpha }\log2$. □

**Lemma** **16.** Let α∈(0,1)∪(1,∞). If $\left(Q_{X}^{*},Q_{Y}^{*}\right)$ achieves the minimum in the definition of $J_{\alpha }\left(X;Y\right)$, then there exist positive normalization constants c and d such that

(237) $$\begin{array}{cc} Q_{X}^{*}\left(x\right) & =c{\left[\sum_{y}P{\left(x,y\right)}^{\alpha }\;Q_{Y}^{*}{\left(y\right)}^{1-\alpha }\right]}^{\frac{1}{\alpha }}\;\forall \;x\in X, \end{array}$$

(238) $$\begin{array}{cc} Q_{Y}^{*}\left(y\right) & =d{\left[\sum_{x}P{\left(x,y\right)}^{\alpha }\;Q_{X}^{*}{\left(x\right)}^{1-\alpha }\right]}^{\frac{1}{\alpha }}\;\forall \;y\in Y, \end{array}$$

with the conventions of (44). The case α=∞ is similar: if $\left(Q_{X}^{*},Q_{Y}^{*}\right)$ achieves the minimum in the definition of $J_{\infty }\left(X;Y\right)$, then there exist positive normalization constants c and d such that

(239) $$\begin{array}{cc} Q_{X}^{*}\left(x\right) & =c\max_{y}\frac{P(x,y)}{Q_{Y}^{*}\left(y\right)}\;\forall \;x\in X, \end{array}$$

(240) $$\begin{array}{cc} Q_{Y}^{*}\left(y\right) & =d\max_{x}\frac{P(x,y)}{Q_{X}^{*}\left(x\right)}\;\forall \;y\in Y, \end{array}$$

with the conventions of (44). (If α=1, then $Q_{X}^{*}=P_{X}$ and $Q_{Y}^{*}=P_{Y}$ by Proposition 8.) Thus, for all α∈(0,∞], both inclusions $\mathrm{supp}\left(Q_{X}^{*}\right)\subseteq \mathrm{supp}\left(P_{X}\right)$ and $\mathrm{supp}\left(Q_{Y}^{*}\right)\subseteq \mathrm{supp}\left(P_{Y}\right)$ hold.

**Proof.**  If $\left(Q_{X}^{*},Q_{Y}^{*}\right)$ achieves the minimum in the definition of $J_{\alpha }\left(X;Y\right)$, then

(241) $$\begin{array}{c} \min_{Q_{Y}}D_{\alpha }\left(P_{XY}∥Q_{X}^{*}Q_{Y}\right)=D_{\alpha }\left(P_{XY}∥Q_{X}^{*}Q_{Y}^{*}\right). \end{array}$$

Hence, (238) and (240) follow from (76) and (78) of Proposition 9 because $D_{\alpha }\left(P_{XY}∥Q_{X}^{*}Q_{Y}^{*}\right)=J_{\alpha }\left(X;Y\right)$ is finite. Swapping the roles of $Q_{X}$ and $Q_{Y}$ establishes (237) and (239). For α∈(0,1)∪(1,∞) the claimed inclusions follow from (237) and (238); for α=∞ from (239) and (240); and for α=1 from Proposition 8. □

**Lemma** **17.** *For all α∈(0,∞],*

(242) $$\begin{array}{c} J_{\alpha }\left(X;Y\right)=\min_{Q_{X}}\phi_{\alpha }\left(Q_{X}\right), \end{array}$$

*where $\phi_{\alpha }\left(Q_{X}\right)$ is defined as*

(243) $$\begin{array}{c} \phi_{\alpha }\left(Q_{X}\right)≜\min_{Q_{Y}}D_{\alpha }\left(P_{XY}∥Q_{X}Q_{Y}\right) \end{array}$$

*and is given explicitly as follows: for α∈(0,1)∪(1,∞),*

(244) $$\begin{array}{c} \phi_{\alpha }\left(Q_{X}\right)=\frac{\alpha }{\alpha -1}\log\sum_{y}{\left[\sum_{x}P{\left(x,y\right)}^{\alpha }\;Q_{X}{\left(x\right)}^{1-\alpha }\right]}^{\frac{1}{\alpha }}, \end{array}$$

*with the conventions of (44); and for α∈{1,∞},*

(245) $$\begin{array}{cc} \phi_{1}\left(Q_{X}\right) & =D(P_{XY}∥Q_{X}P_{Y}), \end{array}$$

(246) $$\begin{array}{cc} \phi_{\infty }\left(Q_{X}\right) & =\log\sum_{y}\max_{x}\frac{P(x,y)}{Q_{X}\left(x\right)}, \end{array}$$

*with the conventions of (44). For every $\alpha \in [\frac{1}{2},\infty ]$, the mapping $Q_{X}\mapsto \phi_{\alpha }\left(Q_{X}\right)$ is convex. For $\alpha \in (0,\frac{1}{2})$, the mapping need not be convex.*

**Proof.**  We first establish (242) and (244)–(246): (242) follows from the definition of $J_{\alpha }\left(X;Y\right)$; (244) and (246) follow from Proposition 9; and (245) holds because

(247) $$\begin{array}{cc} \min_{Q_{Y}}D\left(P_{XY}∥Q_{X}Q_{Y}\right) & =\min_{Q_{Y}}\;\left[D\left(P_{XY}∥Q_{X}P_{Y}\right)+D\left(P_{Y}∥Q_{Y}\right)\right] \end{array}$$

(248) $$\begin{array}{c} =D(P_{XY}∥Q_{X}P_{Y}), \end{array}$$

where (247) follows from a simple computation, and (248) holds because $D(P_{Y}∥Q_{Y})\geq 0$ with equality if $Q_{Y}=P_{Y}$.

We now show that the mapping $Q_{X}\mapsto \phi_{\alpha }\left(Q_{X}\right)$ is convex for every $\alpha \in [\frac{1}{2},\infty ]$. To that end, let $\alpha \in [\frac{1}{2},\infty ]$, let $\lambda ,\lambda^{\prime }\in \left[0,1\right]$ with $\lambda +\lambda^{\prime }=1$, and let $Q_{X},Q_{X}^{\prime }\in P\left(X\right)$. Let ${\hat{Q}}_{Y}$ and ${\hat{Q}}_{Y}^{\prime }$ be PMFs that achieve the minimum in the definitions of $\phi_{\alpha }\left(Q_{X}\right)$ and $\phi_{\alpha }\left(Q_{X}^{\prime }\right)$, respectively. Then,

(249) $$\begin{array}{cc} \phi_{\alpha }\left(\lambda \;Q_{X}+\lambda^{\prime }\;Q_{X}^{\prime }\right) & \leq D_{\alpha }\left(P_{XY}∥\left(\lambda Q_{X}+\lambda^{\prime }Q_{X}^{\prime }\right)\left(\lambda {\hat{Q}}_{Y}+\lambda^{\prime }{\hat{Q}}_{Y}^{\prime }\right)\right) \end{array}$$

(250) $$\begin{array}{c} \leq \lambda \;D_{\alpha }\left(P_{XY}∥Q_{X}{\hat{Q}}_{Y}\right)+\lambda^{\prime }\;D_{\alpha }\left(P_{XY}∥Q_{X}^{\prime }{\hat{Q}}_{Y}^{\prime }\right) \end{array}$$

(251) $$\begin{array}{c} =\lambda \;\phi_{\alpha }\left(Q_{X}\right)+\lambda^{\prime }\;\phi_{\alpha }\left(Q_{X}^{\prime }\right), \end{array}$$

where (249) holds by the definition of $\phi_{\alpha }\left(\cdot \right)$; (250) holds because $D_{\alpha }\left(P_{XY}∥Q_{X}Q_{Y}\right)$ is convex in the pair $\left(Q_{X},Q_{Y}\right)$ for $\alpha \in [\frac{1}{2},\infty ]$ (Lemma 15); and (251) follows from our choice of ${\hat{Q}}_{Y}$ and ${\hat{Q}}_{Y}^{\prime }$.

Finally, we show that the mapping $Q_{X}\mapsto \phi_{\alpha }\left(Q_{X}\right)$ need not be convex for $\alpha \in (0,\frac{1}{2})$. Let *X* be uniformly distributed over {0,1}, and let Y=X. Then, for all $\alpha \in (0,\frac{1}{2})$,

(252) $$\begin{array}{c} \phi_{\alpha }\left((0.5,0.5)\right)>0.5\;\phi_{\alpha }\left((1,0)\right)+0.5\;\phi_{\alpha }\left((0,1)\right), \end{array}$$

because the LHS of (252) is equal to log2, and the RHS of (252) is equal to $\frac{\alpha }{1-\alpha }\log2$. □

**Lemma** **18.** *For all α∈(0,1)∪(1,∞],*

(253) $$\begin{array}{c} J_{\alpha }\left(X;Y\right)=\left\{\begin{array}{cc} \min_{R_{XY}\in P\left(X\times Y\right)}\psi_{\alpha }\left(R_{XY}\right) & if\;\alpha \in (0,1),\\ \max_{R_{XY}\in P\left(X\times Y\right)}\psi_{\alpha }\left(R_{XY}\right) & if\;\alpha \in (1,\infty ], \end{array}\right. \end{array}$$

*where*

(254) $$\begin{array}{c} \psi_{\alpha }\left(R_{XY}\right)≜\left\{\begin{array}{cc} D\left(R_{XY}∥R_{X}R_{Y}\right)+\frac{\alpha }{1-\alpha }D\left(R_{XY}∥P_{XY}\right) & if\;\alpha \in (0,1)\cup (1,\infty ),\\ D\left(R_{XY}∥R_{X}R_{Y}\right)-D\left(R_{XY}∥P_{XY}\right) & if\;\alpha =\infty . \end{array}\right. \end{array}$$

*For every α∈(1,∞], the mapping $R_{XY}\mapsto \psi_{\alpha }\left(R_{XY}\right)$ is concave. For all α∈(1,∞] and all $R_{XY}\in P\left(X\times Y\right)$, the statement $J_{\alpha }\left(X;Y\right)=\psi_{\alpha }\left(R_{XY}\right)$ is equivalent to $\psi_{\alpha }\left(R_{XY}\right)=D_{\alpha }\left(P_{XY}∥R_{X}R_{Y}\right)$.*

**Proof.**  For α∈(0,1)∪(1,∞), (253) follows from Lemma 8 by dividing by 1−α, which is positive or negative depending on whether α is smaller than or greater than one. For α=∞, we establish (253) as follows: By Lemma 10, its LHS is continuous at α=∞. We argue below that its RHS is continuous at α=∞, i.e., that

(255) $$\begin{array}{c} \lim_{\alpha \to \infty }\max_{R_{XY}}\psi_{\alpha }\left(R_{XY}\right)=\max_{R_{XY}}\psi_{\infty }\left(R_{XY}\right). \end{array}$$

Because (253) holds for α∈(1,∞) and because both its sides are continuous at α=∞, it must also hold for α=∞.

We now establish (255). Let $R_{XY}^{*}$ be a PMF that achieves the maximum on the RHS of (255). Then, for all α>1,

(256) $$\begin{array}{cc} \psi_{\infty }\left(R_{XY}^{*}\right) & =\max_{R_{XY}}\psi_{\infty }\left(R_{XY}\right) \end{array}$$

(257) $$\begin{array}{c} \geq \max_{R_{XY}}\psi_{\alpha }\left(R_{XY}\right) \end{array}$$

(258) $$\begin{array}{c} \geq \psi_{\alpha }\left(R_{XY}^{*}\right), \end{array}$$

where (257) holds because, by (254), $\psi_{\infty }\left(R_{XY}\right)=\psi_{\alpha }\left(R_{XY}\right)+\frac{1}{\alpha -1}\;D\left(R_{XY}∥P_{XY}\right)\geq \psi_{\alpha }\left(R_{XY}\right)$ for all $R_{XY}\in P\left(X\times Y\right)$. By (254), $\alpha \mapsto \psi_{\alpha }\left(R_{XY}^{*}\right)$ is continuous at α=∞, so the RHS of (258) approaches $\psi_{\infty }\left(R_{XY}^{*}\right)$ as α tends to infinity, and (255) follows from the sandwich theorem.

We now show that $R_{XY}\mapsto \psi_{\alpha }\left(R_{XY}\right)$ is concave for α∈(1,∞]. A simple computation reveals that for all α∈(1,∞),

(259) $$\begin{array}{c} \psi_{\alpha }\left(R_{XY}\right)=H\left(R_{X}\right)+H\left(R_{Y}\right)+\frac{1}{\alpha -1}\;H\left(R_{XY}\right)+\frac{\alpha }{\alpha -1}\sum_{x,y}R_{XY}\left(x,y\right)\log P\left(x,y\right). \end{array}$$

Because the entropy is a concave function and because a nonnegative weighted sum of concave functions is concave, this implies that $\psi_{\alpha }\left(R_{XY}\right)$ is concave in $R_{XY}$ for α∈(1,∞). By (254), $\alpha \mapsto \psi_{\alpha }\left(R_{XY}\right)$ is continuous at α=∞, so $\psi_{\alpha }\left(R_{XY}\right)$ is concave in $R_{XY}$ also for α=∞.

We next show that if α∈(1,∞] and $\psi_{\alpha }\left(R_{XY}\right)=D_{\alpha }\left(P_{XY}∥R_{X}R_{Y}\right)$, then $J_{\alpha }\left(X;Y\right)=\psi_{\alpha }\left(R_{XY}\right)$. Let α∈(1,∞], and let $R_{XY}$ be a PMF that satisfies $\psi_{\alpha }\left(R_{XY}\right)=D_{\alpha }\left(P_{XY}∥R_{X}R_{Y}\right)$. Then,

(260) $$\begin{array}{cc} \psi_{\alpha }\left(R_{XY}\right) & \leq J_{\alpha }\left(X;Y\right) \end{array}$$

(261) $$\begin{array}{c} \leq D_{\alpha }\left(P_{XY}∥R_{X}R_{Y}\right), \end{array}$$

where (260) follows from (253), and (261) holds by the definition of $J_{\alpha }\left(X;Y\right)$. Because $\psi_{\alpha }\left(R_{XY}\right)$ is equal to $D_{\alpha }\left(P_{XY}∥R_{X}R_{Y}\right)$, both inequalities hold with equality, which implies the claim.

Finally, we show that if α∈(1,∞] and $J_{\alpha }\left(X;Y\right)=\psi_{\alpha }\left(R_{XY}\right)$, then $\psi_{\alpha }\left(R_{XY}\right)=D_{\alpha }\left(P_{XY}∥R_{X}R_{Y}\right)$. We first consider α∈(1,∞). Let $R_{XY}$ be a PMF that satisfies $J_{\alpha }\left(X;Y\right)=\psi_{\alpha }\left(R_{XY}\right)$, and let $Q_{X}^{*}$ and $Q_{Y}^{*}$ be PMFs that achieve the minimum in the definition of $J_{\alpha }\left(X;Y\right)$. Then,

(262) $$\begin{array}{cc} J_{\alpha }\left(X;Y\right) & =\psi_{\alpha }\left(R_{XY}\right) \end{array}$$

(263) $$\begin{array}{c} =D\left(R_{XY}∥R_{X}R_{Y}\right)+\frac{\alpha }{1-\alpha }\;D\left(R_{XY}∥P_{XY}\right) \end{array}$$

(264) $$\begin{array}{c} \leq D\left(R_{XY}∥Q_{X}^{*}Q_{Y}^{*}\right)+\frac{\alpha }{1-\alpha }\;D\left(R_{XY}∥P_{XY}\right) \end{array}$$

(265) $$\begin{array}{c} \leq D_{\alpha }\left(P_{XY}∥Q_{X}^{*}Q_{Y}^{*}\right) \end{array}$$

(266) $$\begin{array}{c} =J_{\alpha }\left(X;Y\right), \end{array}$$

where (264) follows from Proposition 8, and (265) follows from [22] (Theorem 30). Thus, all inequalities hold with equality. Because (264) holds with equality, $Q_{X}^{*}=R_{X}$ and $Q_{Y}^{*}=R_{Y}$ by Proposition 8. Hence, $\psi_{\alpha }\left(R_{XY}\right)=D_{\alpha }\left(P_{XY}∥Q_{X}^{*}Q_{Y}^{*}\right)=D_{\alpha }\left(P_{XY}∥R_{X}R_{Y}\right)$ as desired. We now consider α=∞. Here, (262)–(266) remain valid after replacing $\frac{\alpha }{1-\alpha }$ by −1. (Now, (265) follows from a short computation.) Consequently, $\psi_{\alpha }\left(R_{XY}\right)=D_{\alpha }\left(P_{XY}∥R_{X}R_{Y}\right)$ holds also for α=∞.

**Lemma** **19.** *For all α∈(0,1)∪(1,∞),*

(267) $$\begin{array}{c} J_{\alpha }\left(X;Y\right)=\min_{R_{X}\ll P_{X}}\frac{1}{\alpha -1}\left[D_{\frac{\alpha }{\alpha -1}}\left(P_{X}∥R_{X}\right)-\alpha \;E_{0}\left(\frac{1-\alpha }{\alpha },R_{X}\right)\right], \end{array}$$

*where the minimization is over all PMFs $R_{X}$ satisfying $R_{X}\ll P_{X}$$\left(i.e.,\;\mathrm{supp}\left(R_{X}\right)\subseteq \mathrm{supp}\left(P_{X}\right)\right)$; $D_{\alpha }\left(P∥Q\right)$ for negative α is given by (54); and Gallager’s $E_{0}$ function [29] is defined as*

(268) $$\begin{array}{c} E_{0}\left(\rho ,R_{X}\right)≜-\log\sum_{y}{\left[\sum_{x}R_{X}\left(x\right)\;P{\left(y|x\right)}^{\frac{1}{1+\rho }}\right]}^{1+\rho }. \end{array}$$

**Proof.**  Let α∈(0,1)∪(1,∞), and define the set $R≜\{R_{X}\in P\left(X\right):\mathrm{supp}\left(R_{X}\right)\subseteq \mathrm{supp}\left(P_{X}\right)\}$. We establish (267) by showing that for all $R_{X}\in R$,

(269) $$\begin{array}{c} \frac{1}{\alpha -1}\left[D_{\frac{\alpha }{\alpha -1}}\left(P_{X}∥R_{X}\right)-\alpha \;E_{0}\left(\frac{1-\alpha }{\alpha },R_{X}\right)\right]\geq J_{\alpha }\left(X;Y\right), \end{array}$$

with equality for some $R_{X}\in R$.

Fix $R_{X}\in R$. If the LHS of (269) is infinite, then (269) holds trivially. Otherwise, define the PMF ${\hat{Q}}_{X}$ as

(270) $$\begin{array}{c} {\hat{Q}}_{X}\left(x\right)≜\frac{P_{X}{\left(x\right)}^{\frac{\alpha }{\alpha -1}}R_{X}{\left(x\right)}^{\frac{1}{1-\alpha }}}{\sum_{x^{\prime }\in X}P_{X}{\left(x^{\prime }\right)}^{\frac{\alpha }{\alpha -1}}R_{X}{\left(x^{\prime }\right)}^{\frac{1}{1-\alpha }}}, \end{array}$$

where we use the convention that $0^{\frac{\alpha }{\alpha -1}}\cdot 0^{\frac{1}{1-\alpha }}=0$. (The RHS of (270) is finite whenever the LHS of (269) is finite.) Then, (269) holds because

(271) $$\begin{array}{cc} J_{\alpha }\left(X;Y\right) & =\min_{Q_{X}}\frac{\alpha }{\alpha -1}\log\sum_{y}{\left[\sum_{x}P{\left(x,y\right)}^{\alpha }\;Q_{X}{\left(x\right)}^{1-\alpha }\right]}^{\frac{1}{\alpha }} \end{array}$$

(272) $$\begin{array}{c} \leq \frac{\alpha }{\alpha -1}\log\sum_{y}{\left[\sum_{x}P{\left(x,y\right)}^{\alpha }\;{\hat{Q}}_{X}{\left(x\right)}^{1-\alpha }\right]}^{\frac{1}{\alpha }} \end{array}$$

(273) $$\begin{array}{c} =\log\sum_{x}P_{X}{\left(x\right)}^{\frac{\alpha }{\alpha -1}}R_{X}{\left(x\right)}^{\frac{1}{1-\alpha }}+\frac{\alpha }{\alpha -1}\log\sum_{y}{\left[\sum_{x}R_{X}\left(x\right)\;P{\left(y|x\right)}^{\alpha }\right]}^{\frac{1}{\alpha }} \end{array}$$

(274) $$\begin{array}{c} =\frac{1}{\alpha -1}\left[D_{\frac{\alpha }{\alpha -1}}\left(P_{X}∥R_{X}\right)-\alpha \;E_{0}\left(\frac{1-\alpha }{\alpha },R_{X}\right)\right], \end{array}$$

where (271) follows from Lemma 17, and (273) follows from (270) using some algebra. It remains to show that there exists an $R_{X}\in R$ for which (272) holds with equality. To that end, let $Q_{X}^{*}$ be a PMF that achieves the minimum on the RHS of (271), and define the PMF $R_{X}$ as

(275) $$\begin{array}{c} R_{X}\left(x\right)≜\frac{P_{X}{\left(x\right)}^{\alpha }\;Q_{X}^{*}{\left(x\right)}^{1-\alpha }}{\sum_{x^{\prime }\in X}P_{X}{\left(x^{\prime }\right)}^{\alpha }\;Q_{X}^{*}{\left(x^{\prime }\right)}^{1-\alpha }}, \end{array}$$

where we use the convention that $0^{\alpha }\cdot 0^{1-\alpha }=0$. Because $\mathrm{supp}\left(Q_{X}^{*}\right)\subseteq \mathrm{supp}\left(P_{X}\right)$ (Lemma 16), the definitions (275) and (270) imply that ${\hat{Q}}_{X}=Q_{X}^{*}$. Hence, (272) holds with equality for this $R_{X}\in R$.

**Lemma** **20.** *For every $\alpha \in (\frac{1}{2},\infty ]$, the mapping $\left(Q_{X},Q_{Y}\right)\mapsto D_{\alpha }\left(P_{XY}∥Q_{X}Q_{Y}\right)$ has a unique minimizer. This need not be the case when $\alpha \in [0,\frac{1}{2}]$.*

**Proof.**  First consider $\alpha \in (\frac{1}{2},1)$. Let $\left(Q_{X}^{*},Q_{Y}^{*}\right)$ and $\left({\hat{Q}}_{X},{\hat{Q}}_{Y}\right)$ be pairs of PMFs that both minimize $\left(Q_{X},Q_{Y}\right)\mapsto D_{\alpha }\left(P_{XY}∥Q_{X}Q_{Y}\right)$. We establish uniqueness by arguing that $\left(Q_{X}^{*},Q_{Y}^{*}\right)$ and $\left({\hat{Q}}_{X},{\hat{Q}}_{Y}\right)$ must be identical. Observe that

(276) $$\begin{array}{cc} J_{\alpha }\left(X;Y\right) & \leq D_{\alpha }\left(P_{XY}∥\left(0.5\;Q_{X}^{*}+0.5\;{\hat{Q}}_{X}\right)\left(0.5\;Q_{Y}^{*}+0.5\;{\hat{Q}}_{Y}\right)\right) \end{array}$$

(277) $$\begin{array}{c} \leq 0.5\;D_{\alpha }\left(P_{XY}∥Q_{X}^{*}Q_{Y}^{*}\right)+0.5\;D_{\alpha }\left(P_{XY}∥{\hat{Q}}_{X}{\hat{Q}}_{Y}\right) \end{array}$$

(278) $$\begin{array}{c} =J_{\alpha }\left(X;Y\right), \end{array}$$

where (276) holds by the definition of $J_{\alpha }\left(X;Y\right)$, and (277) follows from Lemma 15. Hence, (277) holds with equality, which implies that (228) in the proof of Lemma 15 holds with equality, i.e.,

(279) $$\begin{array}{c} \sum_{x,y}P{\left(x,y\right)}^{\alpha }\sqrt{0.5\;Q_{X}^{*}{\left(x\right)}^{2(1-\alpha )}+0.5\;{\hat{Q}}_{X}{\left(x\right)}^{2(1-\alpha )}}\;\sqrt{0.5\;Q_{Y}^{*}{\left(y\right)}^{2(1-\alpha )}+0.5\;{\hat{Q}}_{Y}{\left(y\right)}^{2(1-\alpha )}}\\ \;=\sum_{x,y}P{\left(x,y\right)}^{\alpha }{\left[0.5\;Q_{X}^{*}\left(x\right)+0.5\;{\hat{Q}}_{X}\left(x\right)\right]}^{1-\alpha }\;\sqrt{0.5\;Q_{Y}^{*}{\left(y\right)}^{2(1-\alpha )}+0.5\;{\hat{Q}}_{Y}{\left(y\right)}^{2(1-\alpha )}}. \end{array}$$

We first argue that $Q_{X}^{*}={\hat{Q}}_{X}$. Since $Q_{X}^{*}$ and ${\hat{Q}}_{X}$ are PMFs, it suffices to show that $Q_{X}^{*}\left(x\right)={\hat{Q}}_{X}\left(x\right)$ for every $x\in \mathrm{supp}({\hat{Q}}_{X})$. Let $\hat{x}\in \mathrm{supp}\left({\hat{Q}}_{X}\right)$. Because $\mathrm{supp}\left({\hat{Q}}_{X}\right)\subseteq \mathrm{supp}\left(P_{X}\right)$ (Lemma 16), there exists a $\hat{y}\in Y$ such that $P(\hat{x},\hat{y})>0$. Again by Lemma 16, this implies that ${\hat{Q}}_{Y}\left(\hat{y}\right)>0$. Because the mapping $z\mapsto z^{2(1-\alpha )}$ is strictly concave on $R_{\geq 0}$ for $\alpha \in (\frac{1}{2},1)$, it follows from (279) that $Q_{X}^{*}\left(\hat{x}\right)={\hat{Q}}_{X}\left(\hat{x}\right)$. Swapping the roles of $Q_{X}$ and $Q_{Y}$, we obtain that $Q_{Y}^{*}={\hat{Q}}_{Y}$.

For α=1, the minimizer is unique by Proposition 8 because $D_{1}\left(P_{XY}∥Q_{X}Q_{Y}\right)=D\left(P_{XY}∥Q_{X}Q_{Y}\right)$.

Now consider α∈(1,∞]. Here, we establish uniqueness via the characterization of $J_{\alpha }\left(X;Y\right)$ provided by Lemma 18. Let $\psi_{\alpha }\left(R_{XY}\right)$ be defined as in Lemma 18. Let $R_{XY}$ be a PMF that satisfies $J_{\alpha }\left(X;Y\right)=\psi_{\alpha }\left(R_{XY}\right)$, and let $\left(Q_{X}^{*},Q_{Y}^{*}\right)$ be a pair of PMFs that minimizes $\left(Q_{X},Q_{Y}\right)\mapsto D_{\alpha }\left(P_{XY}∥Q_{X}Q_{Y}\right)$. If α∈(1,∞), then (264) in the proof of Lemma 18 holds with equality, i.e.,

(280) $$\begin{array}{c} D\left(R_{XY}∥R_{X}R_{Y}\right)+\frac{\alpha }{1-\alpha }\;D\left(R_{XY}∥P_{XY}\right)=D\left(R_{XY}∥Q_{X}^{*}Q_{Y}^{*}\right)+\frac{\alpha }{1-\alpha }\;D\left(R_{XY}∥P_{XY}\right). \end{array}$$

Because the LHS of (280) is finite, Proposition 8 implies that $Q_{X}^{*}=R_{X}$ and $Q_{Y}^{*}=R_{Y}$, thus the minimizer is unique. As shown in the proof of Lemma 18, (280) remains valid for α=∞ after replacing $\frac{\alpha }{1-\alpha }$ by −1, thus the same argument establishes the uniqueness for α=∞.

Finally, we show that, for $\alpha \in [0,\frac{1}{2}]$, the mapping $\left(Q_{X},Q_{Y}\right)\mapsto D_{\alpha }\left(P_{XY}∥Q_{X}Q_{Y}\right)$ can have more than one minimizer. Let *X* be uniformly distributed over {0,1}, and let Y=X. Then, for all $\alpha \in [0,\frac{1}{2}]$,

(281) $$\begin{array}{cc} J_{\alpha }\left(X;Y\right) & =\frac{\alpha }{1-\alpha }\log2 \end{array}$$

(282) $$\begin{array}{c} =D_{\alpha }\left(P_{XY}∥\left(1,0\right)\left(1,0\right)\right) \end{array}$$

(283) $$\begin{array}{c} =D_{\alpha }\left(P_{XY}∥\left(0,1\right)\left(0,1\right)\right), \end{array}$$

where (281) follows from Lemma 11. □

**Lemma** **21.** *For every α∈[0,∞], the minimum in the definition of $K_{\alpha }\left(X;Y\right)$ in (2) exists and is finite.*

**Proof.**  Let α∈[0,∞], and denote by $U_{X}$ and $U_{Y}$ the uniform distribution over X and Y, respectively. Then $\inf_{Q_{X},\;Q_{Y}}\Delta_{\alpha }\left(P_{XY}∥Q_{X}Q_{Y}\right)$ is finite because $\Delta_{\alpha }\left(P_{XY}∥U_{X}U_{Y}\right)$ is finite and because the relative α-entropy is nonnegative (Proposition 5). For α∈(0,∞), the minimum exists because the set P(X)×P(Y) is compact and the mapping $\left(Q_{X},Q_{Y}\right)\mapsto \Delta_{\alpha }\left(P_{XY}∥Q_{X}Q_{Y}\right)$ is continuous. For α∈{0,∞}, the minimum exists because $\left(Q_{X},Q_{Y}\right)\mapsto \Delta_{\alpha }\left(P_{XY}∥Q_{X}Q_{Y}\right)$ takes on only a finite number of values: if α=0, then $\Delta_{\alpha }\left(P_{XY}∥Q_{X}Q_{Y}\right)$ depends on $Q_{X}Q_{Y}$ only via $\mathrm{supp}(Q_{X}Q_{Y})\subseteq X\times Y$; and if α=∞, then $\Delta_{\alpha }\left(P_{XY}∥Q_{X}Q_{Y}\right)$ depends on $Q_{X}Q_{Y}$ only via $\mathrm{argmax}(Q_{X}Q_{Y})\subseteq X\times Y$. □

**Lemma** **22.** *For all α∈[0,∞], $K_{\alpha }\left(X;Y\right)\geq 0$. If α∈(0,∞), then $K_{\alpha }\left(X;Y\right)=0$ if and only if X and Y are independent (nonnegativity).*

**Proof.**  The nonnegativity follows from the definition of $K_{\alpha }\left(X;Y\right)$ because the relative α-entropy is nonnegative for α∈[0,∞] (Proposition 5). If *X* and *Y* are independent, then $P_{XY}=P_{X}P_{Y}$, and the choice $Q_{X}=P_{X}$ and $Q_{Y}=P_{Y}$ in the definition of $K_{\alpha }\left(X;Y\right)$ achieves $K_{\alpha }\left(X;Y\right)=0$. Conversely, if $K_{\alpha }\left(X;Y\right)=0$, then there exist PMFs $Q_{X}^{*}$ and $Q_{Y}^{*}$ satisfying $\Delta_{\alpha }\left(P_{XY}∥Q_{X}^{*}Q_{Y}^{*}\right)=0$. If, in addition, α∈(0,∞), then $P_{XY}=Q_{X}^{*}Q_{Y}^{*}$ by Proposition 5, and hence *X* and *Y* are independent. □

**Lemma** **23.** *For all α∈[0,∞], $K_{\alpha }\left(X;Y\right)=K_{\alpha }\left(Y;X\right)$ (symmetry).*

**Proof.**  The definition of $K_{\alpha }\left(X;Y\right)$ is symmetric in *X* and *Y*. □

**Lemma** **24.** *For all α∈(0,∞),*

(284) $$\begin{array}{c} K_{\alpha }\left(X;Y\right)+H_{\alpha }\left(X,Y\right)=\min_{Q_{X},\;Q_{Y}}-\log M_{\frac{\alpha -1}{\alpha }}\left(Q_{X},Q_{Y}\right), \end{array}$$

*where $M_{\beta }\left(Q_{X},Q_{Y}\right)$ is the following weighted power mean [30] (Chapter III): For β∈R∖{0},*

(285) $$\begin{array}{c} M_{\beta }\left(Q_{X},Q_{Y}\right)≜{\left[\sum_{x,y}P\left(x,y\right){\left[Q_{X}\left(x\right)\;Q_{Y}\left(y\right)\right]}^{\beta }\right]}^{\frac{1}{\beta }}, \end{array}$$

*where for β<0, we read $P\left(x,y\right){\left[Q_{X}\left(x\right)\;Q_{Y}\left(y\right)\right]}^{\beta }$ as $P\left(x,y\right)/{\left[Q_{X}\left(x\right)\;Q_{Y}\left(y\right)\right]}^{-\beta }$ and use the conventions (44); and for β=0, using the convention $0^{0}=1$,*

(286) $$\begin{array}{c} M_{0}\left(Q_{X},Q_{Y}\right)≜\prod_{x,y}{\left[Q_{X}\left(x\right)\;Q_{Y}\left(y\right)\right]}^{P(x,y)}. \end{array}$$

**Proof.**  Let α∈(0,∞), and define the PMF ${\tilde{P}}_{XY}$ as

(287) $$\begin{array}{c} {\tilde{P}}_{XY}\left(x,y\right)≜\frac{P_{XY}{\left(x,y\right)}^{\alpha }}{\sum_{(x^{\prime },y^{\prime })\in X\times Y}P_{XY}{\left(x^{\prime },y^{\prime }\right)}^{\alpha }}. \end{array}$$

Then,

(288) $$\begin{array}{cc} K_{\alpha }\left(X;Y\right) & =J_{\frac{1}{\alpha }}\left(\tilde{X};\tilde{Y}\right) \end{array}$$

(289) $$\begin{array}{c} =\min_{Q_{X},\;Q_{Y}}D_{\frac{1}{\alpha }}\left({\tilde{P}}_{XY}∥Q_{X}Q_{Y}\right), \end{array}$$

where (288) follows from Proposition 7, and (289) follows from the definition of $J_{1/\alpha }\left(\tilde{X};\tilde{Y}\right)$. A simple computation reveals that for all PMFs $Q_{X}$ and $Q_{Y}$,

(290) $$\begin{array}{c} D_{\frac{1}{\alpha }}\left({\tilde{P}}_{XY}∥Q_{X}Q_{Y}\right)=-\log M_{\frac{\alpha -1}{\alpha }}\left(Q_{X},Q_{Y}\right)-H_{\alpha }\left(X,Y\right). \end{array}$$

Hence, (284) follows from (289) and (290). □

**Lemma** **25.** *For α=0,*

(291) $$\begin{array}{cc} K_{0}\left(X;Y\right) & =\log\frac{\left|\mathrm{supp}\left(P_{X}P_{Y}\right)\right|}{\left|\mathrm{supp}\left(P_{XY}\right)\right|} \end{array}$$

(292) $$\begin{array}{c} \geq \min_{Q_{X},\;Q_{Y}}\log\max_{\left(x,y\right)\in \mathrm{supp}\left(P_{XY}\right)}\frac{1}{Q_{X}\left(x\right)\;Q_{Y}\left(y\right)}-\log\;\left|\mathrm{supp}\left(P_{XY}\right)\right| \end{array}$$

(293) $$\begin{array}{c} =\lim_{\alpha ↓0}K_{\alpha }\left(X;Y\right), \end{array}$$

*where in the RHS of (292), we use the conventions (44). The inequality can be strict, so $\alpha \mapsto K_{\alpha }\left(X;Y\right)$ need not be continuous at α=0.*

**Proof.**  We first prove (291). Recall that

(294) $$\begin{array}{c} \Delta_{0}\left(P_{XY}∥Q_{X}Q_{Y}\right)=\left\{\begin{array}{cc} \log\frac{\left|\mathrm{supp}\left(Q_{X}Q_{Y}\right)\right|}{\left|\mathrm{supp}\left(P_{XY}\right)\right|} & \mathrm{if}\;\mathrm{supp}\left(P_{XY}\right)\subseteq \mathrm{supp}\left(Q_{X}Q_{Y}\right),\\ \infty & \mathrm{otherwise}. \end{array}\right. \end{array}$$

Observe that $\Delta_{0}\left(P_{XY}∥Q_{X}Q_{Y}\right)$ is finite only if $\mathrm{supp}\left(P_{X}\right)\subseteq \mathrm{supp}\left(Q_{X}\right)$ and $\mathrm{supp}\left(P_{Y}\right)\subseteq \mathrm{supp}\left(Q_{Y}\right)$. For such PMFs $Q_{X}$ and $Q_{Y}$, we have $\left|\mathrm{supp}\left(Q_{X}Q_{Y}\right)\right|\geq \left|\mathrm{supp}\left(P_{X}P_{Y}\right)\right|$. Thus, for all PMFs $Q_{X}$ and $Q_{Y}$,

(295) $$\begin{array}{c} \Delta_{0}\left(P_{XY}∥Q_{X}Q_{Y}\right)\geq \log\frac{\left|\mathrm{supp}\left(P_{X}P_{Y}\right)\right|}{\left|\mathrm{supp}\left(P_{XY}\right)\right|}. \end{array}$$

Choosing $Q_{X}=P_{X}$ and $Q_{Y}=P_{Y}$ achieves equality in (295), which establishes (291).

We now show (292). Let $Q_{X}$ and $Q_{Y}$ be the uniform distributions over $\mathrm{supp}(P_{X})$ and $\mathrm{supp}(P_{Y})$, respectively. Then,

(296) $$\begin{array}{c} \log\max_{\left(x,y\right)\in \mathrm{supp}\left(P_{XY}\right)}\frac{1}{Q_{X}\left(x\right)\;Q_{Y}\left(y\right)}-\log\;\left|\mathrm{supp}\left(P_{XY}\right)\right|=\log\frac{\left|\mathrm{supp}\left(P_{X}P_{Y}\right)\right|}{\left|\mathrm{supp}\left(P_{XY}\right)\right|}, \end{array}$$

and hence (292) holds.

We next establish (293). To that end, define

(297) $$\begin{array}{c} \tau ≜\min_{\left(x,y\right)\in \mathrm{supp}\left(P_{XY}\right)}P\left(x,y\right). \end{array}$$

We bound $K_{\alpha }\left(X;Y\right)+H_{\alpha }\left(X,Y\right)$ as follows: For all α∈(0,1),

(298) $$\begin{array}{cc} K_{\alpha }\left(X;Y\right)+H_{\alpha }\left(X,Y\right) & =\min_{Q_{X},\;Q_{Y}}\frac{\alpha }{1-\alpha }\log\sum_{x,y}P\left(x,y\right){\left[Q_{X}\left(x\right)\;Q_{Y}\left(y\right)\right]}^{\frac{\alpha -1}{\alpha }} \end{array}$$

(299) $$\begin{array}{c} \geq \min_{Q_{X},\;Q_{Y}}\frac{\alpha }{1-\alpha }\log\sum_{\left(x,y\right)\in \mathrm{supp}\left(P_{XY}\right)}\tau \;{\left[Q_{X}\left(x\right)\;Q_{Y}\left(y\right)\right]}^{\frac{\alpha -1}{\alpha }} \end{array}$$

(300) $$\begin{array}{c} \geq \min_{Q_{X},\;Q_{Y}}\frac{\alpha }{1-\alpha }\log\max_{\left(x,y\right)\in \mathrm{supp}\left(P_{XY}\right)}\tau \;{\left[Q_{X}\left(x\right)\;Q_{Y}\left(y\right)\right]}^{\frac{\alpha -1}{\alpha }} \end{array}$$

(301) $$\begin{array}{c} =\min_{Q_{X},\;Q_{Y}}\log\max_{\left(x,y\right)\in \mathrm{supp}\left(P_{XY}\right)}\frac{1}{Q_{X}\left(x\right)\;Q_{Y}\left(y\right)}-\frac{\alpha }{1-\alpha }\log\frac{1}{\tau }, \end{array}$$

where (298) follows from Lemma 24. Similarly, for all α∈(0,1),

(302) $$\begin{array}{cc} K_{\alpha }\left(X;Y\right)+H_{\alpha }\left(X,Y\right) & =\min_{Q_{X},\;Q_{Y}}\frac{\alpha }{1-\alpha }\log\sum_{x,y}P\left(x,y\right){\left[Q_{X}\left(x\right)\;Q_{Y}\left(y\right)\right]}^{\frac{\alpha -1}{\alpha }} \end{array}$$

(303) $$\begin{array}{c} \leq \min_{Q_{X},\;Q_{Y}}\frac{\alpha }{1-\alpha }\log\max_{\left(x,y\right)\in \mathrm{supp}\left(P_{XY}\right)}{\left[Q_{X}\left(x\right)\;Q_{Y}\left(y\right)\right]}^{\frac{\alpha -1}{\alpha }} \end{array}$$

(304) $$\begin{array}{c} =\min_{Q_{X},\;Q_{Y}}\log\max_{\left(x,y\right)\in \mathrm{supp}\left(P_{XY}\right)}\frac{1}{Q_{X}\left(x\right)\;Q_{Y}\left(y\right)}, \end{array}$$

where (302) is the same as (298). Now (293) follows from (301), (304), and the sandwich theorem because $\lim_{\alpha ↓0}\frac{\alpha }{1-\alpha }\log\frac{1}{\tau }=0$ and because $\lim_{\alpha ↓0}H_{\alpha }\left(X,Y\right)=\log\;\left|\mathrm{supp}\left(P_{XY}\right)\right|$ (Proposition 3).

Finally, we provide an example for which (292) holds with strict inequality. Let X={1,2,3}, let Y={1,2}, and let (X,Y) be uniformly distributed over {(1,1),(2,2),(3,1)}. The LHS of (292) then equals log2. Using

(305) $$\begin{array}{cc} Q_{X}\left(x\right) & ≜\left\{\begin{array}{cc} 0.28 & \mathrm{if}\;x\in \{1,3\},\\ 0.44 & \mathrm{if}\;x=2, \end{array}\right. \end{array}$$

(306) $$\begin{array}{cc} Q_{Y}\left(y\right) & ≜\left\{\begin{array}{cc} 0.60 & \mathrm{if}\;y=1,\\ 0.40 & \mathrm{if}\;y=2, \end{array}\right. \end{array}$$

we see that the RHS of (292) is upper bounded by $\log\frac{5.952\ldots }{3}$, which is smaller than log2. □

**Lemma** **26.** *$K_{1}\left(X;Y\right)=I\left(X;Y\right)$.*

**Proof.**  The claim follows from Proposition 8 because $\Delta_{1}\left(P_{XY}∥Q_{X}Q_{Y}\right)$ in the definition of $K_{1}\left(X;Y\right)$ is equal to $D(P_{XY}∥Q_{X}Q_{Y})$. □

**Lemma** **27.** *Let f:{1,…,|X|}→X and g:{1,…,|Y|}→Y be bijective functions, and let B be the |X|×|Y| matrix whose Row-i Column-j entry $B_{i,j}$ equals $P_{XY}\left(f\left(i\right),g\left(j\right)\right)$. Then,*

(307) $$\begin{array}{c} K_{2}\left(X;Y\right)=-2\log\sigma_{1}\left(B\right)-H_{2}\left(X,Y\right), \end{array}$$

*where $\sigma_{1}\left(B\right)$ denotes the largest singular value of B. (Because the singular values of a matrix are invariant under row and column permutations, the result does not depend on f or g.)*

**Proof.**  Let $\left(\tilde{X},\tilde{Y}\right)$ be distributed according to the joint PMF

(308) $$\begin{array}{c} {\tilde{P}}_{XY}\left(x,y\right)≜{\left[\beta \;P_{XY}\left(x,y\right)\right]}^{2}, \end{array}$$

where

(309) $$\begin{array}{c} \beta ≜{\left[\sum_{x,y}P_{XY}{\left(x,y\right)}^{2}\right]}^{-\frac{1}{2}}. \end{array}$$

Then,

(310) $$\begin{array}{cc} K_{2}\left(X;Y\right) & =J_{\frac{1}{2}}\left(\tilde{X};\tilde{Y}\right) \end{array}$$

(311) $$\begin{array}{c} =-2\log\sigma_{1}\left(\beta \;B\right) \end{array}$$

(312) $$\begin{array}{c} =-2\log\left[\beta \;\sigma_{1}\left(B\right)\right] \end{array}$$

(313) $$\begin{array}{c} =-2\log\sigma_{1}\left(B\right)-H_{2}\left(X,Y\right), \end{array}$$

where (310) follows from Proposition 7; (311) follows from Lemma 6 and (308); (312) holds because β>0; and (313) follows from the definition of $H_{2}\left(X,Y\right)$. □

**Lemma** **28.** *$K_{\infty }\left(X;Y\right)=0$.*

**Proof.**  Let the pair $\left(\hat{x},\hat{y}\right)$ be such that $P\left(\hat{x},\hat{y}\right)=\max_{x,y}P\left(x,y\right)$, and define the PMFs ${\hat{Q}}_{X}$ and ${\hat{Q}}_{Y}$ as ${\hat{Q}}_{X}\left(x\right)=𝟙\left\{x=\hat{x}\right\}$ and ${\hat{Q}}_{Y}\left(y\right)=𝟙\left\{y=\hat{y}\right\}$. Then, $\Delta_{\infty }\left(P_{XY}∥{\hat{Q}}_{X}{\hat{Q}}_{Y}\right)=0$, so $K_{\infty }\left(X;Y\right)\leq 0$. Because $K_{\infty }\left(X;Y\right)\geq 0$ (Lemma 22), this implies $K_{\infty }\left(X;Y\right)=0$. □

**Lemma** **29.** *The mapping $\alpha \mapsto K_{\alpha }\left(X;Y\right)$ need not be monotonic on [0,∞].*

**Proof.**  Let $P_{XY}$ be such that $\mathrm{supp}(P_{XY})=X\times Y$ and I(X;Y)>0. Then,

(314) $$\begin{array}{cc} K_{0}\left(X;Y\right) & =0, \end{array}$$

(315) $$\begin{array}{cc} K_{1}\left(X;Y\right) & >0, \end{array}$$

(316) $$\begin{array}{cc} K_{\infty }\left(X;Y\right) & =0, \end{array}$$

which follow from Lemmas 25, 26, and 28, respectively. Thus, $\alpha \mapsto K_{\alpha }\left(X;Y\right)$ is not monotonic on [0,∞]. □

**Lemma** **30.** *The mapping $\alpha \mapsto K_{\alpha }\left(X;Y\right)+H_{\alpha }\left(X,Y\right)$ is nonincreasing on [0,∞].*

**Proof.**  We first show the monotonicity for α∈(0,∞). To that end, let $\alpha ,\alpha^{\prime }\in \left(0,\infty \right)$ with $\alpha \leq \alpha^{\prime }$, and let $M_{\beta }\left(Q_{X},Q_{Y}\right)$ be defined as in (285) and (286). Then, for all PMFs $Q_{X}$ and $Q_{Y}$,

(317) $$\begin{array}{c} M_{\frac{\alpha -1}{\alpha }}\left(Q_{X},Q_{Y}\right)\leq M_{\frac{\alpha^{\prime }-1}{\alpha^{\prime }}}\left(Q_{X},Q_{Y}\right), \end{array}$$

which follows from the power mean inequality [30] (III 3.1.1 Theorem 1) because $\frac{\alpha -1}{\alpha }\leq \frac{\alpha^{\prime }-1}{\alpha^{\prime }}$. Hence,

(318) $$\begin{array}{cc} K_{\alpha }\left(X;Y\right)+H_{\alpha }\left(X,Y\right) & =\min_{Q_{X},\;Q_{Y}}-\log M_{\frac{\alpha -1}{\alpha }}\left(Q_{X},Q_{Y}\right) \end{array}$$

(319) $$\begin{array}{c} \geq \min_{Q_{X},\;Q_{Y}}-\log M_{\frac{\alpha^{\prime }-1}{\alpha^{\prime }}}\left(Q_{X},Q_{Y}\right) \end{array}$$

(320) $$\begin{array}{c} =K_{\alpha^{\prime }}\left(X;Y\right)+H_{\alpha^{\prime }}\left(X,Y\right), \end{array}$$

where (318) and (320) follow from Lemma 24, and (319) follows from (317).

The monotonicity extends to α=0 because

(321) $$\begin{array}{cc} K_{0}\left(X;Y\right)+H_{0}\left(X,Y\right) & \geq \lim_{\alpha ↓0}K_{\alpha }\left(X;Y\right)+H_{0}\left(X,Y\right) \end{array}$$

(322) $$\begin{array}{c} =\lim_{\alpha ↓0}\left[K_{\alpha }\left(X;Y\right)+H_{\alpha }\left(X,Y\right)\right], \end{array}$$

where (321) follows from Lemma 25, and (322) holds because $\alpha \mapsto H_{\alpha }\left(X,Y\right)$ is continuous at α=0 (Proposition 3).

The monotonicity extends to α=∞ because for all α∈(0,∞),

(323) $$\begin{array}{cc} K_{\alpha }\left(X;Y\right)+H_{\alpha }\left(X,Y\right) & \geq H_{\alpha }\left(X,Y\right) \end{array}$$

(324) $$\begin{array}{c} \geq H_{\infty }\left(X,Y\right) \end{array}$$

(325) $$\begin{array}{c} =K_{\infty }\left(X;Y\right)+H_{\infty }\left(X,Y\right), \end{array}$$

where (323) holds because $K_{\alpha }\left(X;Y\right)\geq 0$ (Lemma 22); (324) holds because $H_{\alpha }\left(X,Y\right)$ is nonincreasing in α (Proposition 3); and (325) holds because $K_{\infty }\left(X;Y\right)=0$ (Lemma 28). □

**Lemma** **31.** *The mapping $\alpha \mapsto K_{\alpha }\left(X;Y\right)$ is continuous on (0,∞]. (See Lemma 25 for the behavior at α=0.)*

**Proof.**  Because $\alpha \mapsto H_{\alpha }\left(X,Y\right)$ is continuous on [0,∞] (Proposition 3), it suffices to show that the mapping $\alpha \mapsto K_{\alpha }\left(X;Y\right)+H_{\alpha }\left(X,Y\right)$ is continuous on (0,∞]. We first show that it is continuous on (0,1)∪(1,∞) by showing that $\alpha \mapsto \left(1-\frac{1}{\alpha }\right)\left[K_{\alpha }\left(X;Y\right)+H_{\alpha }\left(X,Y\right)\right]$ is concave and hence continuous on (0,∞). For a fixed α∈(0,∞), let $\left(\tilde{X},\tilde{Y}\right)$ be distributed according to the joint PMF

(326) $$\begin{array}{c} {\tilde{P}}_{XY}\left(x,y\right)≜\frac{P_{XY}{\left(x,y\right)}^{\alpha }}{\sum_{(x^{\prime },y^{\prime })\in X\times Y}P_{XY}{\left(x^{\prime },y^{\prime }\right)}^{\alpha }}. \end{array}$$

Then, for all α∈(0,∞),

(327) $$\begin{array}{c} \left(1-\frac{1}{\alpha }\right)\left[K_{\alpha }\left(X;Y\right)+H_{\alpha }\left(X,Y\right)\right]\\ \;=\left(1-\frac{1}{\alpha }\right)J_{\frac{1}{\alpha }}\left(\tilde{X};\tilde{Y}\right)+\left(1-\frac{1}{\alpha }\right)H_{\alpha }\left(X,Y\right) \end{array}$$

(328) $$\begin{array}{c} \;=\min_{R_{XY}}\left[\left(1-\frac{1}{\alpha }\right)D\left(R_{XY}∥R_{X}R_{Y}\right)+\frac{1}{\alpha }\;D\left(R_{XY}∥{\tilde{P}}_{XY}\right)+\left(1-\frac{1}{\alpha }\right)H_{\alpha }\left(X,Y\right)\right] \end{array}$$

(329) $$\begin{array}{c} \;=\min_{R_{XY}}\left[\left(1-\frac{1}{\alpha }\right)D\left(R_{XY}∥R_{X}R_{Y}\right)+\left(1-\frac{1}{\alpha }\right)H\left(R_{XY}\right)+D\left(R_{XY}∥P_{XY}\right)\right], \end{array}$$

where (327) follows from Proposition 7; (328) follows from Lemma 8; and (329) follows from a short computation. For every $R_{XY}\in P\left(X\times Y\right)$, the expression in square brackets on the RHS of (329) is concave in α because the mapping $\alpha \mapsto 1-\frac{1}{\alpha }$ is concave on (0,∞) and because $D(R_{XY}∥R_{X}R_{Y})$ and $H(R_{XY})$ are nonnegative. The pointwise minimum preserves the concavity, thus the LHS of (327) is concave in α and hence continuous in α∈(0,∞). This implies that $\alpha \mapsto K_{\alpha }\left(X;Y\right)+H_{\alpha }\left(X,Y\right)$ and hence $\alpha \mapsto K_{\alpha }\left(X;Y\right)$ is continuous on (0,1)∪(1,∞).

We now establish continuity at α=∞. Let $\left(\hat{x},\hat{y}\right)$ be such that $P\left(\hat{x},\hat{y}\right)=\max_{x,y}P\left(x,y\right)$; define the PMFs ${\hat{Q}}_{X}$ and ${\hat{Q}}_{Y}$ as ${\hat{Q}}_{X}\left(x\right)≜𝟙\left\{x=\hat{x}\right\}$ and ${\hat{Q}}_{Y}\left(y\right)≜𝟙\left\{y=\hat{y}\right\}$; and let $M_{\beta }\left(Q_{X},Q_{Y}\right)$ be defined as in (285). Then, for all α∈(1,∞),

(330) $$\begin{array}{cc} K_{\infty }\left(X;Y\right)+H_{\infty }\left(X,Y\right) & \leq K_{\alpha }\left(X;Y\right)+H_{\alpha }\left(X,Y\right) \end{array}$$

(331) $$\begin{array}{c} \leq -\log M_{\frac{\alpha -1}{\alpha }}\left({\hat{Q}}_{X},{\hat{Q}}_{Y}\right) \end{array}$$

(332) $$\begin{array}{c} =\frac{\alpha }{\alpha -1}\;H_{\infty }\left(X,Y\right) \end{array}$$

(333) $$\begin{array}{c} =K_{\infty }\left(X;Y\right)+\frac{\alpha }{\alpha -1}\;H_{\infty }\left(X,Y\right), \end{array}$$

where (330) holds because $K_{\alpha }\left(X;Y\right)+H_{\alpha }\left(X,Y\right)$ is nonincreasing in α (Lemma 30); (331) follows from Lemma 24; (332) follows from the definitions of $M_{\beta }\left(Q_{X},Q_{Y}\right)$ in (285) and $H_{\infty }\left(X,Y\right)$ in (46); and (333) holds because $K_{\infty }\left(X;Y\right)=0$ (Lemma 28). Because $\lim_{\alpha \to \infty }\frac{\alpha }{\alpha -1}=1$, (330)–(333) and the sandwich theorem imply that $\alpha \mapsto K_{\alpha }\left(X;Y\right)+H_{\alpha }\left(X,Y\right)$ is continuous at α=∞. This and the continuity of $\alpha \mapsto H_{\alpha }\left(X,Y\right)$ at α=∞ (Proposition 3) establish the continuity of $\alpha \mapsto K_{\alpha }\left(X;Y\right)$ at α=∞.

It remains to show the continuity at α=1. Let $\alpha \in \left(\frac{4}{5},1\right)\cup \left(1,\frac{4}{3}\right)$, and define $\delta ≜\frac{\left|\alpha -1\right|}{\alpha }\in \left(0,\frac{1}{4}\right)$. (These definitions ensure that on the RHS of (340) ahead, 1−4δ will be positive.) Let $M_{\beta }\left(Q_{X},Q_{Y}\right)$ be defined as in (285) and (286). Then, for all PMFs $Q_{X}$ and $Q_{Y}$,

(334) $$\begin{array}{cc} M_{\frac{\alpha -1}{\alpha }}\left(Q_{X},Q_{Y}\right) & \leq M_{\delta }\left(Q_{X},Q_{Y}\right) \end{array}$$

(335) $$\begin{array}{c} ={\left[\sum_{x,y}P\left(x,y\right)\;{\left[P_{X}\left(x\right)\;P_{Y}\left(y\right)\right]}^{\delta }{\left[\frac{Q_{X}\left(x\right)\;Q_{Y}\left(y\right)}{P_{X}\left(x\right)\;P_{Y}\left(y\right)}\right]}^{\delta }\;\right]}^{\frac{1}{\delta }} \end{array}$$

(336) $$\begin{array}{c} \leq {\left[\sum_{x,y}P\left(x,y\right)\;{\left[P_{X}\left(x\right)\;P_{Y}\left(y\right)\right]}^{2\delta }\right]}^{\frac{1}{2\delta }}\cdot {\left[\sum_{x,y}P\left(x,y\right){\left[\frac{Q_{X}\left(x\right)\;Q_{Y}\left(y\right)}{P_{X}\left(x\right)\;P_{Y}\left(y\right)}\right]}^{2\delta }\;\right]}^{\frac{1}{2\delta }} \end{array}$$

(337) $$\begin{array}{c} \leq {\left[\sum_{x,y}P\left(x,y\right)\;{\left[P_{X}\left(x\right)\;P_{Y}\left(y\right)\right]}^{2\delta }\right]}^{\frac{1}{2\delta }} \end{array}$$

(338) $$\begin{array}{c} =M_{2\delta }\left(P_{X},P_{Y}\right), \end{array}$$

where (334) follows from the power mean inequality [30] (III 3.1.1 Theorem 1) because $\frac{\alpha -1}{\alpha }\leq \delta$; (336) follows from the Cauchy–Schwarz inequality; and (337) holds because

(339) $$\begin{array}{c} {\left[\sum_{x,y}P\left(x,y\right){\left[\frac{Q_{X}\left(x\right)}{P_{X}\left(x\right)}\right]}^{2\delta }{\left[\frac{Q_{Y}\left(y\right)}{P_{Y}\left(y\right)}\right]}^{2\delta }\;\right]}^{\frac{1}{2\delta }}\\ \;\leq {\left[\sum_{x}P_{X}\left(x\right){\left[\frac{Q_{X}\left(x\right)}{P_{X}\left(x\right)}\right]}^{4\delta }\;\right]}^{\frac{1}{4\delta }}\cdot {\left[\sum_{y}P_{Y}\left(y\right){\left[\frac{Q_{Y}\left(y\right)}{P_{Y}\left(y\right)}\right]}^{4\delta }\;\right]}^{\frac{1}{4\delta }} \end{array}$$

(340) $$\begin{array}{c} \;=2^{-D_{1-4\delta }\left(P_{X}∥Q_{X}\right)}\cdot 2^{-D_{1-4\delta }\left(P_{Y}∥Q_{Y}\right)} \end{array}$$

(341) $$\begin{array}{c} \;\leq 1, \end{array}$$

where (339) follows from the Cauchy–Schwarz inequality, and (341) holds because 1−4δ>0 and because the Rényi divergence is nonnegative for positive orders (Proposition 4). Thus, for all $\alpha \in (\frac{4}{5},\frac{4}{3})$,

(342) $$\begin{array}{cc} -\log M_{\frac{2|\alpha -1|}{\alpha }}\left(P_{X},P_{Y}\right) & \leq \min_{Q_{X},\;Q_{Y}}-\log M_{\frac{\alpha -1}{\alpha }}\left(Q_{X},Q_{Y}\right) \end{array}$$

(343) $$\begin{array}{c} \leq -\log M_{\frac{\alpha -1}{\alpha }}\left(P_{X},P_{Y}\right), \end{array}$$

where (342) follows from (338) if α≠1 and from Proposition 8 and a simple computation if α=1. By Lemma 24, this implies that for all $\alpha \in (\frac{4}{5},\frac{4}{3})$,

(344) $$\begin{array}{cc} -\log M_{\frac{2|\alpha -1|}{\alpha }}\left(P_{X},P_{Y}\right) & \leq K_{\alpha }\left(X;Y\right)+H_{\alpha }\left(X,Y\right) \end{array}$$

(345) $$\begin{array}{c} \leq -\log M_{\frac{\alpha -1}{\alpha }}\left(P_{X},P_{Y}\right). \end{array}$$

Because $\beta \mapsto M_{\beta }\left(P_{X},P_{Y}\right)$ is continuous at β=0 [30] (III 1 Theorem 2(b)), (344)–(345) and the sandwich theorem imply that $\alpha \mapsto K_{\alpha }\left(X;Y\right)+H_{\alpha }\left(X,Y\right)$ is continuous at α=1. This and the continuity of $\alpha \mapsto H_{\alpha }\left(X,Y\right)$ at α=1 (Proposition 3) establish the continuity of $\alpha \mapsto K_{\alpha }\left(X;Y\right)$ at α=1. □

**Lemma** **32.** *If X=Y with probability one, then*

(346) $$\begin{array}{c} K_{\alpha }\left(X;Y\right)=\left\{\begin{array}{cc} 2H_{\frac{\alpha }{2-\alpha }}\left(X\right)-H_{\alpha }\left(X\right) & if\;\alpha \in [0,2),\\ \frac{\alpha }{\alpha -1}\;H_{\infty }\left(X\right)-H_{\alpha }\left(X\right) & if\;\alpha \geq 2,\\ 0 & if\;\alpha =\infty . \end{array}\right. \end{array}$$

**Proof.**  We first treat the cases α=0, α=1, and α=∞. For α=0, (346) holds because

(347) $$\begin{array}{cc} K_{0}\left(X;Y\right) & =\log\frac{\left|\mathrm{supp}\left(P_{X}P_{Y}\right)\right|}{\left|\mathrm{supp}\left(P_{XY}\right)\right|} \end{array}$$

(348) $$\begin{array}{c} =\log\;|\mathrm{supp}\left(P_{X}\right)| \end{array}$$

(349) $$\begin{array}{c} =H_{0}\left(X\right), \end{array}$$

where (347) follows from Lemma 25, and (348) holds because the hypothesis Pr[X=Y]=1 implies that $\left|\mathrm{supp}\left(P_{X}P_{Y}\right)\right|={\left|\mathrm{supp}\left(P_{X}\right)\right|}^{2}$ and $\left|\mathrm{supp}\left(P_{XY}\right)\right|=\left|\mathrm{supp}\left(P_{X}\right)\right|$. For α=1, (346) holds because $K_{1}\left(X;Y\right)=I\left(X;Y\right)$ (Lemma 26) and because Pr[X=Y]=1 implies that $I\left(X;Y\right)=H\left(X\right)=H_{1}\left(X\right)$. For α=∞, (346) holds because $K_{\infty }\left(X;Y\right)=0$ (Lemma 28).

Now let α∈(0,1)∪(1,∞), and let $\left(\tilde{X},\tilde{Y}\right)$ be distributed according to the joint PMF

(350) $$\begin{array}{cc} {\tilde{P}}_{XY}\left(x,y\right) & ≜\frac{P_{XY}{\left(x,y\right)}^{\alpha }}{\sum_{(x^{\prime },y^{\prime })\in X\times Y}P_{XY}{\left(x^{\prime },y^{\prime }\right)}^{\alpha }} \end{array}$$

(351) $$\begin{array}{c} =\frac{P_{X}{\left(x\right)}^{\alpha }}{\sum_{x^{\prime }\in X}P_{X}{\left(x^{\prime }\right)}^{\alpha }}\;𝟙\left\{x=y\right\}, \end{array}$$

where (351) holds because $P_{XY}\left(x,y\right)=P_{X}\left(x\right)\;𝟙\left\{x=y\right\}$ for all x∈X and all y∈Y. If α<2, then (346) holds because

(352) $$\begin{array}{cc} K_{\alpha }\left(X;Y\right) & =J_{\frac{1}{\alpha }}\left(\tilde{X};\tilde{Y}\right) \end{array}$$

(353) $$\begin{array}{c} =H_{\frac{1}{2-\alpha }}\left(\tilde{X}\right) \end{array}$$

(354) $$\begin{array}{c} =\frac{2-\alpha }{1-\alpha }\log\sum_{x}{\left[\frac{P_{X}{\left(x\right)}^{\alpha }}{\sum_{x^{\prime }\in X}P_{X}{\left(x^{\prime }\right)}^{\alpha }}\right]}^{\frac{1}{2-\alpha }} \end{array}$$

(355) $$\begin{array}{c} =2H_{\frac{\alpha }{2-\alpha }}\left(X\right)-H_{\alpha }\left(X\right), \end{array}$$

where (352) follows from Proposition 7; (353) follows from Lemma 11 because $\mathrm{Pr}[\tilde{X}=\tilde{Y}]=1$ and because $\frac{1}{\alpha }>\frac{1}{2}$; and (355) follows from a simple computation. If α≥2, then (346) holds because

(356) $$\begin{array}{cc} K_{\alpha }\left(X;Y\right) & =J_{\frac{1}{\alpha }}\left(\tilde{X};\tilde{Y}\right) \end{array}$$

(357) $$\begin{array}{c} =\frac{1}{\alpha -1}\;H_{\infty }\left(\tilde{X}\right) \end{array}$$

(358) $$\begin{array}{c} =\frac{-1}{\alpha -1}\log\max_{x}\frac{P_{X}{\left(x\right)}^{\alpha }}{\sum_{x^{\prime }\in X}P_{X}{\left(x^{\prime }\right)}^{\alpha }} \end{array}$$

(359) $$\begin{array}{c} =\frac{\alpha }{\alpha -1}\;H_{\infty }\left(X\right)-H_{\alpha }\left(X\right), \end{array}$$

where (356) follows from Proposition 7; (357) follows from Lemma 11 because $\mathrm{Pr}[\tilde{X}=\tilde{Y}]=1$ and because $\frac{1}{\alpha }\leq \frac{1}{2}$; and (359) follows from a simple computation. □

**Lemma** **33.** *For every α∈(0,2), the mapping $\left(Q_{X},Q_{Y}\right)\mapsto \Delta_{\alpha }\left(P_{XY}∥Q_{X}Q_{Y}\right)$ in the definition of $K_{\alpha }\left(X;Y\right)$ in (2) has a unique minimizer. This need not be the case when α∈{0}∪[2,∞].*

**Proof.**  Let α∈(0,2). By Proposition 7, $K_{\alpha }\left(X;Y\right)=J_{1/\alpha }\left(\tilde{X};\tilde{Y}\right)$, where the pair $\left(\tilde{X},\tilde{Y}\right)$ is distributed according to the joint PMF ${\tilde{P}}_{XY}$ defined in Proposition 7. The mapping $\left(Q_{X},Q_{Y}\right)\mapsto D_{1/\alpha }\left({\tilde{P}}_{XY}∥Q_{X}Q_{Y}\right)$ in the definition of $J_{1/\alpha }\left(\tilde{X};\tilde{Y}\right)$ has a unique minimizer by Lemma 20 because $\frac{1}{\alpha }>\frac{1}{2}$. By Proposition 6, there is a bijection between the minimizers of $D_{1/\alpha }\left({\tilde{P}}_{XY}∥Q_{X}Q_{Y}\right)$ and $\Delta_{\alpha }\left(P_{XY}∥Q_{X}Q_{Y}\right)$, so the mapping $\left(Q_{X},Q_{Y}\right)\mapsto \Delta_{\alpha }\left(P_{XY}∥Q_{X}Q_{Y}\right)$ also has a unique minimizer.

We next show that for α∈{0}∪[2,∞], the mapping $\left(Q_{X},Q_{Y}\right)\mapsto \Delta_{\alpha }\left(P_{XY}∥Q_{X}Q_{Y}\right)$ can have more than one minimizer. Let *X* be uniformly distributed over {0,1}, and let Y=X. Then, by Lemma 32,

(360) $$\begin{array}{c} K_{\alpha }\left(X;Y\right)=\left\{\begin{array}{cc} \log2 & \mathrm{if}\;\alpha =0,\\ \frac{1}{\alpha -1}\log2 & \mathrm{if}\;\alpha \geq 2,\\ 0 & \mathrm{if}\;\alpha =\infty . \end{array}\right. \end{array}$$

If α=0, then it follows from the definition of $\Delta_{0}\left(P∥Q\right)$ in (56) that $\Delta_{0}\left(P_{XY}∥Q_{X}Q_{Y}\right)=\log2$ whenever $\mathrm{supp}\left(Q_{X}\right)=\mathrm{supp}\left(Q_{Y}\right)=\left\{0,1\right\}$, so the minimizer is not unique. Otherwise, if α∈[2,∞], it can be verified that

(361) $$\begin{array}{cc} \Delta_{\alpha }\left(P_{XY}∥\left(1,0\right)\left(1,0\right)\right) & =\Delta_{\alpha }\left(P_{XY}∥\left(0,1\right)\left(0,1\right)\right) \end{array}$$

(362) $$\begin{array}{c} =\left\{\begin{array}{cc} \frac{1}{\alpha -1}\log2 & \mathrm{if}\;\alpha \geq 2,\\ 0 & \mathrm{if}\;\alpha =\infty , \end{array}\right. \end{array}$$

so the minimizer is not unique in this case either. □

**Lemma** **34.** *If the pairs $\left(X_{1},Y_{1}\right)$ and $\left(X_{2},Y_{2}\right)$ are independent, then $K_{\alpha }\left(X_{1},X_{2};Y_{1},Y_{2}\right)=K_{\alpha }\left(X_{1};Y_{1}\right)+K_{\alpha }\left(X_{2};Y_{2}\right)$ for all α∈[0,∞] (additivity).*

**Proof.**  We first treat the cases α=0 and α=∞. For α=0, the claim is true because

(363) $$\begin{array}{cc} K_{0}\left(X_{1},X_{2};Y_{1},Y_{2}\right) & =\log\frac{\left|\mathrm{supp}\left(P_{X_{1}X_{2}}P_{Y_{1}Y_{2}}\right)\right|}{\left|\mathrm{supp}\left(P_{X_{1}X_{2}Y_{1}Y_{2}}\right)\right|} \end{array}$$

(364) $$\begin{array}{c} =\log\frac{\left|\mathrm{supp}\left(P_{X_{1}}P_{Y_{1}}\right)\right|\cdot \left|\mathrm{supp}\left(P_{X_{2}}P_{Y_{2}}\right)\right|}{\left|\mathrm{supp}\left(P_{X_{1}Y_{1}}\right)\right|\cdot \left|\mathrm{supp}\left(P_{X_{2}Y_{2}}\right)\right|} \end{array}$$

(365) $$\begin{array}{c} =K_{0}\left(X_{1};Y_{1}\right)+K_{0}\left(X_{2};Y_{2}\right), \end{array}$$

where (363) and (365) follow from Lemma 25, and (364) follows from the independence hypothesis $P_{X_{1}X_{2}Y_{1}Y_{2}}=P_{X_{1}Y_{1}}P_{X_{2}Y_{2}}$. For α=∞, the claim is true because $K_{\infty }\left(X;Y\right)=0$ (Lemma 28).

Now let α∈(0,∞), and let $\left({\tilde{X}}_{1},{\tilde{X}}_{2},{\tilde{Y}}_{1},{\tilde{Y}}_{2}\right)$ be distributed according to the joint PMF

(366) $$\begin{array}{cc} {\tilde{P}}_{X_{1}X_{2}Y_{1}Y_{2}}\left(x_{1},x_{2},y_{1},y_{2}\right) & ≜\frac{P_{X_{1}X_{2}Y_{1}Y_{2}}{\left(x_{1},x_{2},y_{1},y_{2}\right)}^{\alpha }}{\sum_{x_{1}^{\prime },x_{2}^{\prime },y_{1}^{\prime },y_{2}^{\prime }}P_{X_{1}X_{2}Y_{1}Y_{2}}{\left(x_{1}^{\prime },x_{2}^{\prime },y_{1}^{\prime },y_{2}^{\prime }\right)}^{\alpha }} \end{array}$$

(367) $$\begin{array}{c} =\frac{P_{X_{1}Y_{1}}{\left(x_{1},y_{1}\right)}^{\alpha }}{\sum_{x_{1}^{\prime },y_{1}^{\prime }}P_{X_{1}Y_{1}}{\left(x_{1}^{\prime },y_{1}^{\prime }\right)}^{\alpha }}\cdot \frac{P_{X_{2}Y_{2}}{\left(x_{2},y_{2}\right)}^{\alpha }}{\sum_{x_{2}^{\prime },y_{2}^{\prime }}P_{X_{2}Y_{2}}{\left(x_{2}^{\prime },y_{2}^{\prime }\right)}^{\alpha }}, \end{array}$$

where (366) follows from the independence hypothesis $P_{X_{1}X_{2}Y_{1}Y_{2}}=P_{X_{1}Y_{1}}P_{X_{2}Y_{2}}$. Then,

(368) $$\begin{array}{cc} K_{\alpha }\left(X_{1},X_{2};Y_{1},Y_{2}\right) & =J_{\frac{1}{\alpha }}\left({\tilde{X}}_{1},{\tilde{X}}_{2};{\tilde{Y}}_{1},{\tilde{Y}}_{2}\right) \end{array}$$

(369) $$\begin{array}{c} =J_{\frac{1}{\alpha }}\left({\tilde{X}}_{1};{\tilde{Y}}_{1}\right)+J_{\frac{1}{\alpha }}\left({\tilde{X}}_{2};{\tilde{Y}}_{2}\right) \end{array}$$

(370) $$\begin{array}{c} =K_{\alpha }\left(X_{1};Y_{1}\right)+K_{\alpha }\left(X_{2};Y_{2}\right), \end{array}$$

where (368) and (370) follow from Proposition 7, and (369) follows from Lemma 12 because the pairs $\left({\tilde{X}}_{1},{\tilde{Y}}_{1}\right)$ and $\left({\tilde{X}}_{2},{\tilde{Y}}_{2}\right)$ are independent by (367). □

**Lemma** **35.** *For all α∈[0,∞], $K_{\alpha }\left(X;Y\right)\leq \log\;\left|X\right|$.*

**Proof.**  For α=0, this is true because

(371) $$\begin{array}{cc} K_{0}\left(X;Y\right) & =\log\frac{\left|\mathrm{supp}\left(P_{X}P_{Y}\right)\right|}{\left|\mathrm{supp}\left(P_{XY}\right)\right|} \end{array}$$

(372) $$\begin{array}{c} \leq \log\frac{\left|X\right|\cdot |\mathrm{supp}\left(P_{Y}\right)|}{\left|\mathrm{supp}\left(P_{XY}\right)\right|} \end{array}$$

(373) $$\begin{array}{c} \leq \log\;|X|, \end{array}$$

where (371) follows from Lemma 25. For α∈(0,∞), the claim is true because

(374) $$\begin{array}{cc} K_{\alpha }\left(X;Y\right) & =J_{\frac{1}{\alpha }}\left(\tilde{X};\tilde{Y}\right) \end{array}$$

(375) $$\begin{array}{c} \leq \log\;|X|, \end{array}$$

where (374) follows from Proposition 7, and (375) follows from Lemma 13. For α=∞, the claim is true because $K_{\infty }\left(X;Y\right)=0$ (Lemma 28). □

**Lemma** **36.** *There exists a Markov chain X⊸−−Y⊸−−Z for which $K_{2}\left(X;Z\right)>K_{2}\left(X;Y\right)$.*

**Proof.**  Let the Markov chain X⊸−−Y⊸−−Z be given by
$P_{Z|Y}$$P_{XY}\left(x,y\right)$y=0y=1x=00.60x=100.4
$P_{Z|Y}$$P_{Z|Y}\left(z|y\right)$z=0z=1y=00.90.1y=101
Using Lemma 27, we see that $K_{2}\left(X;Z\right)\approx 0.605$ bits, which is larger than $K_{2}\left(X;Y\right)\approx 0.531$ bits. □
